# Exploring the Satellitome of the Pest Aphid *Acyrthosiphon pisum* (Hemiptera, Aphididae): Insights Into Genome Organization and Intraspecies Evolution

**DOI:** 10.1093/gbe/evaf104

**Published:** 2025-07-10

**Authors:** Lucas Albuquerque, Diogo Milani, Emiliano Martí, Ana B S M Ferretti, José Manuel Rico-Porras, Pablo Mora, Pedro Lorite, Omid S Ziabari, Jennifer A Brisson, Octavio M Palacios-Gimenez, Diogo C Cabral-de-Mello

**Affiliations:** Departamento de Biologia Geral e Aplicada, UNESP—Univ Estadual Paulista, Instituto de Biociências/IB, Rio Claro, São Paulo, Brazil; Departamento de Biologia Geral e Aplicada, UNESP—Univ Estadual Paulista, Instituto de Biociências/IB, Rio Claro, São Paulo, Brazil; Departamento de Biologia Geral e Aplicada, UNESP—Univ Estadual Paulista, Instituto de Biociências/IB, Rio Claro, São Paulo, Brazil; Department of Biology, University of Rochester, Rochester, NY 14627, USA; Departamento de Biologia Geral e Aplicada, UNESP—Univ Estadual Paulista, Instituto de Biociências/IB, Rio Claro, São Paulo, Brazil; Department of Experimental Biology, Genetics Area, University of Jaén, Jaén 23071, Spain; Departamento de Biologia Geral e Aplicada, UNESP—Univ Estadual Paulista, Instituto de Biociências/IB, Rio Claro, São Paulo, Brazil; Department of Experimental Biology, Genetics Area, University of Jaén, Jaén 23071, Spain; Department of Experimental Biology, Genetics Area, University of Jaén, Jaén 23071, Spain; Department of Biology, University of Rochester, Rochester, NY 14627, USA; Department of Biological Sciences, University of Pittsburgh, Pittsburgh, PA 15260, USA; Department of Biology, University of Rochester, Rochester, NY 14627, USA; Department of Organismal Biology—Systematic Biology, Evolutionary Biology Centre, Uppsala University, Uppsala SE-752 36, Sweden; Institute of Ecology and Evolution, Friedrich Schiller University Jena, Jena 07743, Germany; German Centre for Integrative Biodiversity Research (iDiv) Halle-Jena-Leipzig, Leipzig 04103, Germany; Departamento de Biologia Geral e Aplicada, UNESP—Univ Estadual Paulista, Instituto de Biociências/IB, Rio Claro, São Paulo, Brazil

**Keywords:** biotype, heterochromatin, genome organization, satellite DNA, speciation

## Abstract

Satellite DNAs (satDNAs), ubiquitous sequences in eukaryotic genomes, play a crucial role in genome organization, function, and evolution. The pea aphid *Acyrthosiphon pisum* is a major crop pest, and an emerging model for ecological, developmental, and evolutionary studies. This study characterizes the satellitome of the *A. pisum* to better understand its genomic organization and the evolutionary dynamics of satDNAs at the intraspecies level. The analysis of satDNAs across various host-plant adapted biotypes reveals a general sharing of the satellitome, with significative quantitative differences. We observe the amplification or contraction of specific satDNA families, even in the same biotype, consistent with the fast-evolutionary changes characteristic of these sequences. Rapid differential accumulation of repetitive elements among biotypes together with other factors may contribute to the origin of genetic incompatibilities. At the interspecific level, fluctuations in satDNA abundances, including the loss of certain families, are consistent with the library hypothesis, long-term conservation, and differential amplification of an ancestral satellitome. SatDNAs are considerably enriched in the heterochromatin of the chromosome X (Chr-X) and to a less extent, dispersed across the euchromatic region of all chromosomes. The enrichment of these sequences on Chr-X also features the elements that are most recently amplified and homogenized. This suggests that Chr-X could serve as a fountain of youth for satDNAs, contributing to the “fast-X” effect. Our findings provide valuable insights into genome structure, assembly, and the potential impact of satDNAs on species diversification, as well as the evolution of autosomes and Chr-X.

Significance This study on the pea aphid, *Acyrthosiphon pisum*, sheds light on the impact of satellite DNAs (satDNAs) on genomic structure and evolution. Our analysis of satDNAs across different biotypes highlights the rapid evolutionary dynamics and potential differentiation driven by the amplification and contraction of specific satDNA families. Our findings also underscore the significance of Chr-X as a hub for recent satDNA activity, contributing to our understanding of genome organization and species diversification.

## Introduction

Eukaryotic genomes are teeming with DNA sequences that occur in a repetitive fashion, ranging from tens to several thousands of copies. Repetitive DNAs are found either dispersed across various loci such as DNA transposons and retrotransposons, or clustered in head-to-tail tandem arrays, like satellite DNA (satDNA) (Garrido-Ramos 2017; Louzada et al. 2020; Thakur et al. 2021). Together, these sequences can encompass more than 50% of the genome of an organism (Charlesworth et al. 1994; López-Flores and Garrido-Ramos 2012; Garrido-Ramos 2017; Thakur et al. 2021). In certain instances, satDNAs are the predominant portion of the genome, constituting nearly half of the total DNA content, as found in the beetle *Tenebrio molitor* (Petitpierre et al. 1988) and the blood-sucking bug *Triatoma delpontei* (Mora et al. 2023). SatDNAs are often associated with heterochromatic regions, such as (peri-)centromeres and (sub)telomeres, suggesting a structural and functional role in the establishment and maintenance of this type of chromatin (Plohl et al. 2012; Garrido-Ramos 2017; Thakur et al. 2021). In addition, recent works also reveal satDNAs dispersion within euchromatic regions (Pavlek et al. 2015; Garrido-Ramos 2017; Palacios-Gimenez et al. 2017; Pita et al. 2017; Sproul et al. 2020; Cabral-de-Mello et al. 2023; Rico-Porras et al. 2024).

SatDNAs are among the fastest-evolving sequences in genomes, exhibiting variation in nucleotide sequence composition, sequence complexity (i.e. the emergence of higher-order structures), repeat unit length, and abundance. This dynamic turnover is observed even among closely related species (Garrido-Ramos 2017). The pronounced variation observed in satDNAs may be attributed to the lack of functional constraints, resulting in neutral-like variation encompassing both nucleotide composition and copy number (Lower et al. 2018; McCann et al 2020). Some phylogenetic groups maintain a more similar composition of satDNAs (e.g. Plohl et al. 2010; Escudeiro et al. 2019; Smalec et al. 2019; Palacios-Gimenez et al. 2020), potentially due to biological roles (Fry and Salser 1977) or stochastic events. It is noteworthy that distinct satDNA families populate a genome, collectively forming the satDNA library or satellitome. According to the library hypothesis, the set of satDNAs shared between species is inherited from their common ancestor. Over the course of evolutionary time, this library primarily undergoes differential amplification or contraction processes, but the satDNA families in each species have a common origin (Salser et al. 1976; Fry and Salser 1977; Mestrovic et al. 1998).

The primary evolutionary model proposed to explain some of the dynamics of satDNAs is known as concerted evolution, which leads to the homogenization of satDNA repeats (Dover 1986; Elder and Turner 1995; Ugarković and Plohl 2002; Plohl et al. 2008; Garrido-Ramos 2017). The observed high divergence among satDNA libraries in different lineages can be attributed to the mutational processes associated with concerted evolution. Such divergence can result in the fixation of novel variants within reproductive groups. Molecular drive, produced by several molecular mechanisms including unequal crossover, slippage, rolling-circle replication, gene conversion, and transpositions, plays a pivotal role in facilitating concerted evolution (Dover 1982, 2002; Walsh 1987; Shi et al. 2010). Given the intense dynamism resulting from the previously mentioned mechanisms, the rapid differentiation of satDNAs substantially influences reproductive isolation and the occurrence of hybrid incompatibilities among individuals and populations, thereby playing a significant role in long-term speciation. Incompatibility can manifest as reduced fertility or viability of hybrid offspring due to genetic conflicts arising from the presence of divergent satDNA sequences (Yunis and Yasmineh 1971; Bachmann et al. 1989; Mestrovic et al. 1998; Malik and Henikoff 2001; Ferree and Barbash 2009; Ferree and Prasad 2012; Jagannathan and Yamashita 2021; Cabral-de-Mello and Palacios-Gimenez 2025).

Aphids are hemipteran insects that feed on plant sap representing a significant relevance for agriculture due to their detrimental impact as pests. Given their feeding behavior, aphids can inflict direct harm by damaging the plants or indirectly by acting as vectors for plant viruses (Dedryver et al. 2010). The group possesses diverse karyotypes, ranging from 2*n* = 4 to 2*n* = 72, as observed in the species *Amphorophora* (Blackman 1980; Manicardi et al. 2015). Sex chromosomes systems are predominantly XX/X0, but some species present multiple sex chromosomes (Blackman 1980; Manicardi et al. 2015). The occurrence of diverse karyotypes in aphids may be attributed to both their chromosomal structure and the life cycle characteristics. Aphids possess holocentric chromosomes, which lack a localized centromere. Although several holocentric species among plants and animals have a stable karyotype, this chromosome structure theoretically allows rapid karyotype evolution through the maintenance of distributed centromeric activity even after events of chromosome fission and fusion (Melters et al. 2012). Additionally, holocentric chromosomes may cause inverted meiosis in some species and limitations in the crossing over (Manicardi et al. 2015). Regarding the life cycle of aphids, it is characterized by clonal reproduction through apomictic parthenogenesis, followed by a sexual stage (Dixon 1977). The tolerance of chromosomal rearrangements due to holocentric chromosomes, coupled with prolonged periods of asexuality, may facilitate the persistence of rearranged karyotypes and potentially contribute to speciation events (Mathers et al. 2021).

Despite the recognition of satDNAs as a highly captivating genome component, their evolutionary dynamics covering complete libraries have received limited investigation, particularly at the intraspecies level. Furthermore, most evolutionary models have primarily emerged from empirical analyses conducted on species with monocentric chromosomes. Consequently, only a limited number of studies have explored comprehensive data on a large scale, such as satellitome studies, specifically in species with holocentric chromosomes (e.g. Subirana et al. 2015; Pita et al. 2017; Ribeiro et al. 2017; Cabral-de-Mello et al. 2021, 2023; Mora et al. 2023). Therefore, here we attempt to fill gaps by thoroughly studying the organization of satDNAs in *Acyrthosiphon pisum* genome across different biotypes. In this way, we take advantage of the public availability of sequenced genomes from distinct biotypes (Table 1), which are genetically divergent and adapted to different plant hosts that are able to provide dissimilar microenvironmental conditions or enemies (Ferrari et al. 2008; 2012; Nouhaud et al. 2018). Previous studies based on microsatellite markers revealed that sympatric biotypes that develop in different host species present gradual levels of genetic divergence, ranging from races or even incipient cryptic species, being a few hybrids observed in the field (Peccoud et al. 2009, 2015). Moreover, preference for some host plants and performance and genetic tradeoffs in host adaptation have been identified, with alleles that increase performance on a host-plant tending to decrease the performance in a different host (Hawthorne and Via 2001; Via and Hawthorne 2002). Our analysis consisted of a comparative examination of the satDNAs in 16 genomes of *A. pisum*, including 1 genome each from 10 biotypes and 6 genomes from the biotype *Medicago sativa*. We also compared the sharing of satDNAs with another *Acyrthosiphon* species, plus representatives of 2 other genera, *Myzus persicae* and *Aphis craccivora* (Table 1). In addition, we explored the organization and evolution of satDNA families in the chromosomes of *A. pisum* through bioinformatics analysis of the reference genome and physical mapping by fluorescence in situ hybridization (FISH).

**Table 1:** List of biotypes of *A. pisum* and sister species analyzed for satDNA characterization in this study, along with their accession numbers in the NCBI database, sequencing strategy, C+G content, and submission authors/reference

Sample		Accession number	Sequencer/strategy	Read lenght	GC content	Submitter
*A. pisum* biotypes	*Vicia cracca*	SRX4038418	Illumina HiSeq 2000 WGS Paired	100	29.4%	INRA^a^
	*Onobrychis viciifolia*	SRX4038417	Illumina HiSeq 2000 WGS Paired	100	29.6%	INRA^a^
	*Securigea varia*	SRX4038416	Illumina HiSeq 2000 WGS Paired	100	29.5%	INRA^a^
	*Ononis spinosa*	SRX4038415	Illumina HiSeq 2000 WGS Paired	100	29.5%	INRA^a^
	*Medicago lupulina*	SRX4038414	Illumina HiSeq 2000 WGS Paired	100	31.1%	INRA^a^
	*Melilotus officinalis*	SRX4038413	Illumina HiSeq 2000 WGS Paired	100	29.4%	INRA^a^
	*Lotus corniculatus*	SRX4038412	Illumina HiSeq 2000 WGS Paired	100	29.2%	INRA^a^
	*Genista tinctoria*	SRX4038411	Illumina HiSeq 2000 WGS Paired	100	29.8%	INRA^a^
	*Genista sagitallis*	SRX4038410	Illumina HiSeq 2000 WGS Paired	100	30.0%	INRA^a^
	*Pisum sativum*	SRX3185869	Illumina HiSeq 2000 WGS Paired	100	28.8%	INRA^a^
	*Medicago sativa* Switzerland—SW	SRX4028530	Illumina HiSeq 2000 WGS Paired	100	29.8%	INRA
	*M. sativa* Ranspach—RP	SRX4028529	Illumina HiSeq 2000 WGS Paired	100	30.4%	INRA
	*M. sativa* Mirecourt—MR	SRX4028528	Illumina HiSeq 2000 WGS Paired	100	29.9%	INRA
	*M. sativa* Castelnaudary—CS	SRX4028527	Illumina HiSeq 2000 WGS Paired	100	30.0%	INRA
	*M. sativa* Gers—GE	SRX4028526	Illumina HiSeq 2000 WGS Paired	100	29.7%	INRA
	*M. sativa* Lusignan—LS	SRX4028525	Illumina HiSeq 2000 WGS Paired	100	30.0%	INRA
Sister species	*Acyrthosiphon caraganae*	SRX6674381	Illumina MiSeq WGS Paired	151	30.5%	Belarusian State University
	*Myzus persicae*	SRX6674382	Illumina MiSeq WGS Paired	151	30.3%	Belarusian State University
	*Aphis craccivora*	SRX2888378	Illumina HiSeq 2500 WGS Paired	149	29.7%	Embrapa

Numbers in the collum of submitter indicates works where the genomes were studied.

^a^ Nouhaud et al. (2018). More detailed information for each sample could be consulted in NCBI/SRA database using the accession number provided.

## Results

### Identification and Characterization of satDNAs

A total of 43 families of satDNAs were identified in at least one of the *A. pisum* genomes (accession numbers PV768461-PV768503), each classified as “high confidence” by TAREAN in at least one of the populations studied, with varying consensus sequence lengths and A+T content (Table 2). Upon subjecting our collection of satDNA families to a masking or BLAST search, no significant matches were found with previously reported repeats. The monomer length of the consensus sequences of satDNA families ranged from 19 bp (ApiSat17-19) to 3,661 bp (ApiSat02-3661), with a mean of 564 bp. All satDNAs were rich in A+T content, with a mean content of 66.3%, like the overall A+T content of the *A. pisum* genome, which is ∼73% (The International Aphid Genomics Consortium 2010). The lowest A+T proportion was found in ApiSat12-194 (51%), while the highest was observed in ApiSat39-1227 (78.2%).

**Table 2:** General genomic properties of the 43 satDNA families identified in the genome of *A. pisum* through the analysis of short reads (Table 1)

SatDNA families	Length (bp)	A+T (%)	Average abundance (%)	Abundance range (%)	Abundance fold change	Average divergence (%)	Divergence range (%)	Divergence fold change
ApiSat01-173	173	52.3	0.1383	0.191150–0.104231	1.83	8.52	8.91–6.7	1.33
ApiSat02-3661	3661	56.2	0.1616	0.273540–0.095524	2.86	0.32	0.47–0.21	2.24
ApiSat03-334	334	71.5	0.0447	0.057971–0.035240	1.65	13.26	13.87–12.31	1.13
ApiSat04-253	253	69.3	0.0427	0.077549–0.019925	3.89	3.10	3.48–2.89	1.20
ApiSat05-1174	1174	65.8	0.0267	0.036955–0.021371	1.73	2.79	3.39–1.66	2.04
ApiSat06-1828	1828	60.5	0.0250	0.033703–0.020803	1.62	2.84	3.35–2.26	1.48
ApiSat07-315	315	65.1	0.0288	0.042331–0.020040	2.11	6.80	7.17–6.34	1.13
ApiSat08-1172	1172	65.5	0.0119	0.020723–0.009901	2.09	2.65	3.39–1.54	2.20
ApiSat09-786	786	71.8	0.0089	0.017167–0.005241	3.28	4.00	4.98–3.56	1.40
ApiSat10-929	929	63.2	0.0170	0.024670–0.010525	2.34	4.26	4.86–3.15	1.54
ApiSat11-316	316	53.4	0.0121	0.030134–0.002927	10.29	4.80	7.97–3.11	2.56
ApiSat12-194	194	51.0	0.0249	0.041242–0.012086	3.41	7.28	7.75–6.05	1.28
ApiSat13-668	668	63.4	0.0071	0.011232–0.004478	2.51	4.04	4.52–3.67	1.23
ApiSat14-203	203	59.6	0.0218	0.035920–0.000323	111.07	3.96	10.97–2.79	3.93
ApiSat15-152	152	59.7	0.0145	0.019655–0.006122	3.21	7.25	9.43–5.79	1.63
ApiSat16-718	718	73.8	0.0095	0.013408–0.006543	2.05	1.57	1.70–1.47	1.16
ApiSat17-19	19	62.5	0.0123	0.021968–0.008476	2.59	18.77	19.35–17.76	1.09
ApiSat18-631	631	77.4	0.0107	0.016059–0.008294	1.94	2.52	3.01–2.15	1.40
ApiSat19-248	248	67.1	0.0127	0.023701–0.003437	6.90	3.05	6.24–2.30	2.71
ApiSat20-1634	1634	61.3	0.0101	0.018206–0.005785	3.15	2.28	2.79–1.04	2.68
ApiSat21-48	48	69.0	0.0183	0.027309–0.007675	3.56	5.55	5.97–5.36	1.11
ApiSat22-226	226	63.5	0.0038	0.007058–0.002485	2.84	3.10	5.08–2.41	2.11
ApiSat23-192	192	70.1	0.0231	0.090377–0.006777	13.34	6.95	7.52–6.50	1.16
ApiSat24-326	326	65.9	0.0104	0.017277–0.005532	3.12	5.69	8.07–5.06	1.59
ApiSat25-587	587	59.9	0.0099	0.014151–0.006740	2.10	4.19	5.56–3.23	1.72
ApiSat26-926	926	63.9	0.0097	0.014176–0.004982	2.85	4.35	9.28–2.60	3.57
ApiSat27-498	498	67.9	0.0060	0.009957–0.004175	2.39	3.74	6.99–2.48	2.82
ApiSat28-131	131	61.1	0.0079	0.011139–0.005971	1.87	9.03	10.14–7.86	1.29
ApiSat29-476	476	71.9	0.0051	0.018246–0.002656	6.87	2.28	3.06 –1.21	2.53
ApiSat30-191	191	68.5	0.0197	0.031972–0.005381	5.94	6.61	7.15–6.06	1.18
ApiSat31-146	146	77.5	0.0148	0.022867–0.004921	4.65	6.87	12.73–4.08	3.12
ApiSat32-140	140	74.4	0.0095	0.023724–0.004463	5.32	5.70	7.48–3.26	2.29
ApiSat33-306	306	74.5	0.0069	0.012326–0.003859	3.19	3.41	3.96–2.82	1.40
ApiSat34-549	549	73.4	0.0073	0.011497–0.003714	3.10	4.64	5.38–4.34	1.24
ApiSat35-372	372	75.3	0.0051	0.009664–0.002396	4.03	1.86	2.69–1.18	2.28
ApiSat36-277	277	62.7	0.0012	0.002672–0.000545	4.90	4.84	5.41–4.57	1.18
ApiSat37-755	755	64.6	0.0091	0.062469–0.001835	34.05	9.69	12.55–1.0	12.55
ApiSat38-147	147	77.1	0.0036	0.005533–0.001441	3.84	7.61	11.21–5.81	1.93
ApiSat39-1227	1227	78.2	0.0090	0.014827–0.001318	11.25	8.04	15.97–5.22	3.06
ApiSat40-406	406	54.3	0.0039	0.020741–0.001192	17.40	12.35	19.45–4.11	4.73
ApiSat41-185	185	62.7	0.0016	0.015346–0.000006	2740.36	3.04	12.74–0.61	20.89
ApiSat42-562	562	74.4	0.0005	0.004214–0.000004	1053.43	13.57	20.53–0.39	52.64
ApiSat43-174	174	69.6	0.0013	0.015315–0.005361	2.86	4.92	5.06–4.78	1.06

The table includes the following information for each satDNA family: name, monomer size in base pairs, A+T content, and the mean, range, and fold change of abundance and divergence.

### Abundance and Sequence Divergence of satDNA Families

**Fig. 1.**
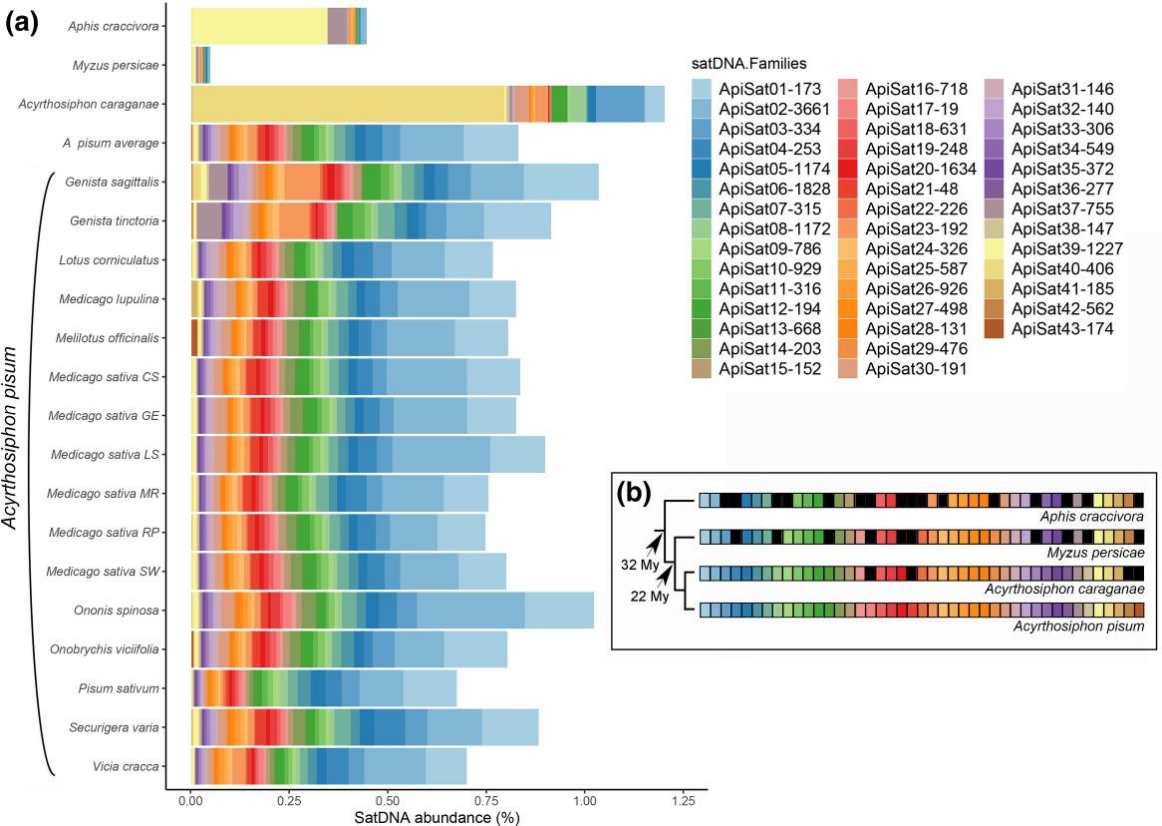
The characterization of the satDNA library in *A. pisum* and its comparison with satDNAs in sister species is detailed as follows: a) SatDNA libraries are visualized using stacked bar plots, illustrating the relative abundances of satDNA families in *A. pisum* genomes and its sister species. The average abundance of satDNA families is shown across *A. pisum* populations and biotype libraries. b) A phylogenetic tree is included for comparing *A. pisum* satDNAs with those other sister species. Absence of specific satDNA families is indicated by black squares. The phylogeny and divergence timeline are based on Mathers et al. (2021). Each color in the tree denotes a distinct satDNA family.

The collection of 43 satDNA families comprised 0.83% of the genome, averaging across all *A. pisum* genomes. This varied from 0.67% in the *Pisum sativum* biotype to 1.03% in the *Genista sagittalis* biotype, resulting in an abundance fold change of 1.53-fold. Following the criterion that each monomer must be present in at least 2 copies, within the collection of 43 satDNA families, 40 were present in all *A. pisum* genomes, with the exceptions of the less abundant satDNAs ApiSat41-185, ApiSat42-562, and ApiSat43-174. ApiSat43-174 was observed only in 2 biotypes, *Onobrychis viciifolia* and *Melilotus officinalis*. ApiSat42-562 was observed only in 3 genomes, belonging to the 2 *Genista* biotypes and *Ononis spinosa* biotype. Finally, ApiSat41-185 was present in 14 genomes and was absent from *M. officinalis* and *Vicia cracca* biotypes. Notably, ApiSat14-203 displayed a large abundance fold change of about 111 times, varying from 0.000323% (*G. sagittalis* biotype) to 0.03592% (*O. spinosa* biotype). In contrast, most other satDNA families exhibited lower abundance fold changes, generally <5 times, including the most abundant satDNA family in the species, which showed an abundance fold change of only 1.83 between 0.19115% in the *G. sagitallis* biotype and 0.104231% in *V. cracca* biotype (Fig. 1a; Table 2; supplementary table S2, Supplementary Material online). Although considerable variation in abundance was detected for the least abundant satDNAs, it is important to note that these differences could be influenced by factors such as library construction and stochastic effects during sequencing and read sampling. Therefore, major conclusions based on these repeats were avoided. The divergence of satDNA sequences among the *A. pisum* biotypes ranged from 0.21% to 19.45%; the highest was observed for ApiSat40-406 in the *Medicago lupina* biotype and the lowest for ApiSat02-3661 in the *G. sagittalis* biotype. Across all genomes, the mean divergence for all satDNAs was 5.32%, with the lowest observed in ApiSat02-3661 (0.32%) and the highest in ApiSat17-19 (18.77%). The range of fold changes in divergence varied greatly among families, with highest increases, i.e. more than 10 times, noted in some, such as in ApiSat37-755 and ApiSat41-185. Controversially, the smallest increase was found in ApiSat17-19, with a divergence of only 1.08 times greater between genomes. Generally, the difference in divergences was <2 times, as observed in 24 out of 43 satDNA families (Table 2; supplementary table S2, Supplementary Material online). The examination conducted within closely related genomes, specifically using 6 populations from the same *Medicago sativa* biotype, revealed discernible quantitative differences among the satDNAs. However, as anticipated, these differences were less pronounced compared to the variations observed across all biotypes. The abundance of the satDNA library ranged from 0.74% in *M. sativa* RP to 0.89% in *M. sativa* LS, with a mean of ∼0.81% (supplementary fig. S1, Supplementary Material online; supplementary table S2, Supplementary Material online; Table 2).

### Interspecific Comparisons

Shared satDNA families among closely related species highlight their potential role in genomic evolution. At the interspecies level, a subset of satDNA families identified in *A. pisum* were also found within the genomes of closely related sister species. Specifically, 27 satDNA families were shared with *A. craccivora*, 33 with *M. persicae*, and 39 with *A. caraganae*, accounting for ∼0.05% to 1.2% of their respective genomes (supplementary table S2, Supplementary Material online; Fig. 1b). This highlights the presence of shared satDNA elements among these related species, further emphasizing their potential significance in genomic evolution and divergence.

### Repeat Landscape Analysis Based on Short Reads

**Fig. 2.**
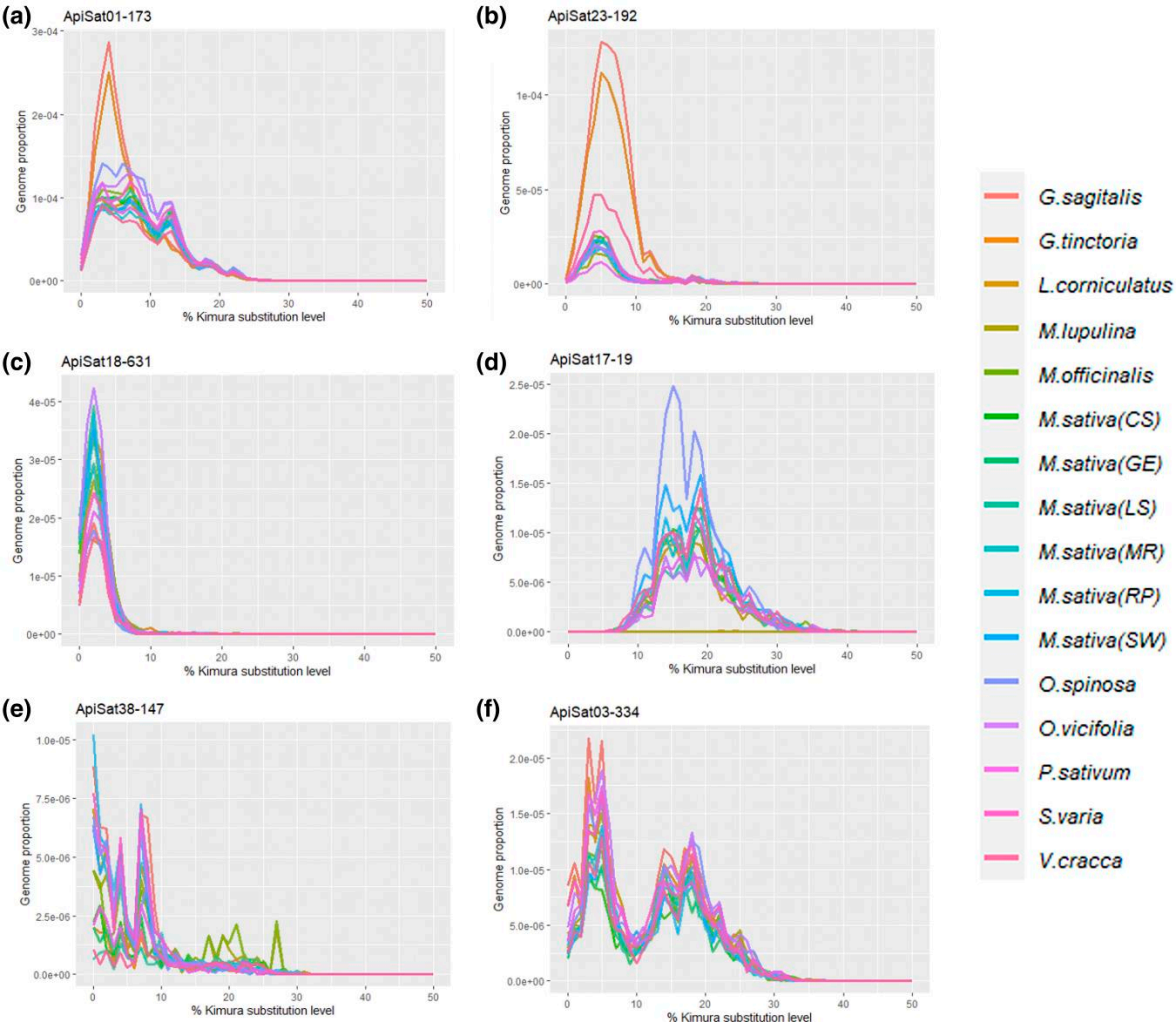
The analysis of satDNA landscapes illustrates the relationship between sequences abundance (%) and divergence (% K2P values) for individual satDNA families. Distinct host plant species are represented by different colors. Several notable patterns emerge from the analysis: (a,b) SatDNA families ApiSat01-173 and ApiSat23-192 show prominent peaks of recent amplification specific to the *Genista* genus, particularly in *G. sagitalis* and *G. tinctoria*. c) ApiSat18-631 consistently demonstrates recent amplification patterns across all biotypes. d) ApiSat17-19 demonstrates a peak of amplification with high K2P divergence values, indicating an ancient origin of amplification. e) ApiSat38-147 shows differential amplification peaks characterized by distinct K2P divergence values. f) ApiSat03-334 shows 2 distinct amplification peaks observed across all biotypes. Each landscape employs color coding to indicate the host plant species associated with the satDNA family analyzed.

To investigate the patterns of differential or recent/ancestral amplification of satDNA families among biotypes, we generated specific satDNA repeat landscapes. These landscapes plot the abundance of each satDNA family against their K2P divergence values (Fig. 2; supplementary fig. S2, Supplementary Material online). SatDNA families that have undergone more recent amplification tend to show greater homogeneity, reflected by low K2P values. Conversely, older satDNA families, which have not been recently amplified, are expected to exhibit higher variability, indicated by higher K2P values due to accumulation of mutations. We observed common waves of amplification for some satDNA families among the biotypes and differential amplification for specific biotypes. For example, satDNA families like ApiSat01-173, ApiSat23-192, and ApiSat18-631 displayed similar low K2P divergence values across all biotypes, suggesting more recent amplification (Fig. 2a-c). In the case of ApiSat01-173 and ApiSat23-192, the amplification in *Genista* genus biotypes was higher compared to other biotypes (Fig. 2a and b). A similar pattern of amplification for all biotypes, including 2 distinct waves (1 older than the other), was observed for ApiSat03-334 (Fig. 2f). Such amplification patterns were consistent across biotypes for some satDNA families, as depicted in supplementary fig. S2, Supplementary Material online. On the other hand, some satDNA families exhibited a discernible pattern of homogenization and more recent amplification. Finally, other differential patterns are exemplified by ApiSat17-19 that shows amplification peaks with high K2P divergence values (Fig. 2d) and ApiSat38-147 with differential amplification peaks characterized by distinct K2P divergence values (Fig. 2e).

### Repeat Landscape Analysis Based on the Genome Assembly

We identified 42 out of the 43 satDNA families in the assembled genome of *A. pisum* using RepeatMasker analysis, highlighting implications for understanding genome assembly limitations. The family ApiSat43-174 was not detected in the genome assembly (supplementary table S3, Supplementary Material online). The absence of this family is likely due to the exclusion of certain repeats during the assembly process, potentially underestimating the actual abundance of satDNAs per chromosome, a phenomenon observed in other insect species (e.g. Oppert et al. 2023; Mora et al. 2024; Rico-Porras et al. 2024). Nevertheless, since our main focus is to compare satDNAs between chromosomes, this technical limitation does not compromise our conclusions.

**Fig. 3.**
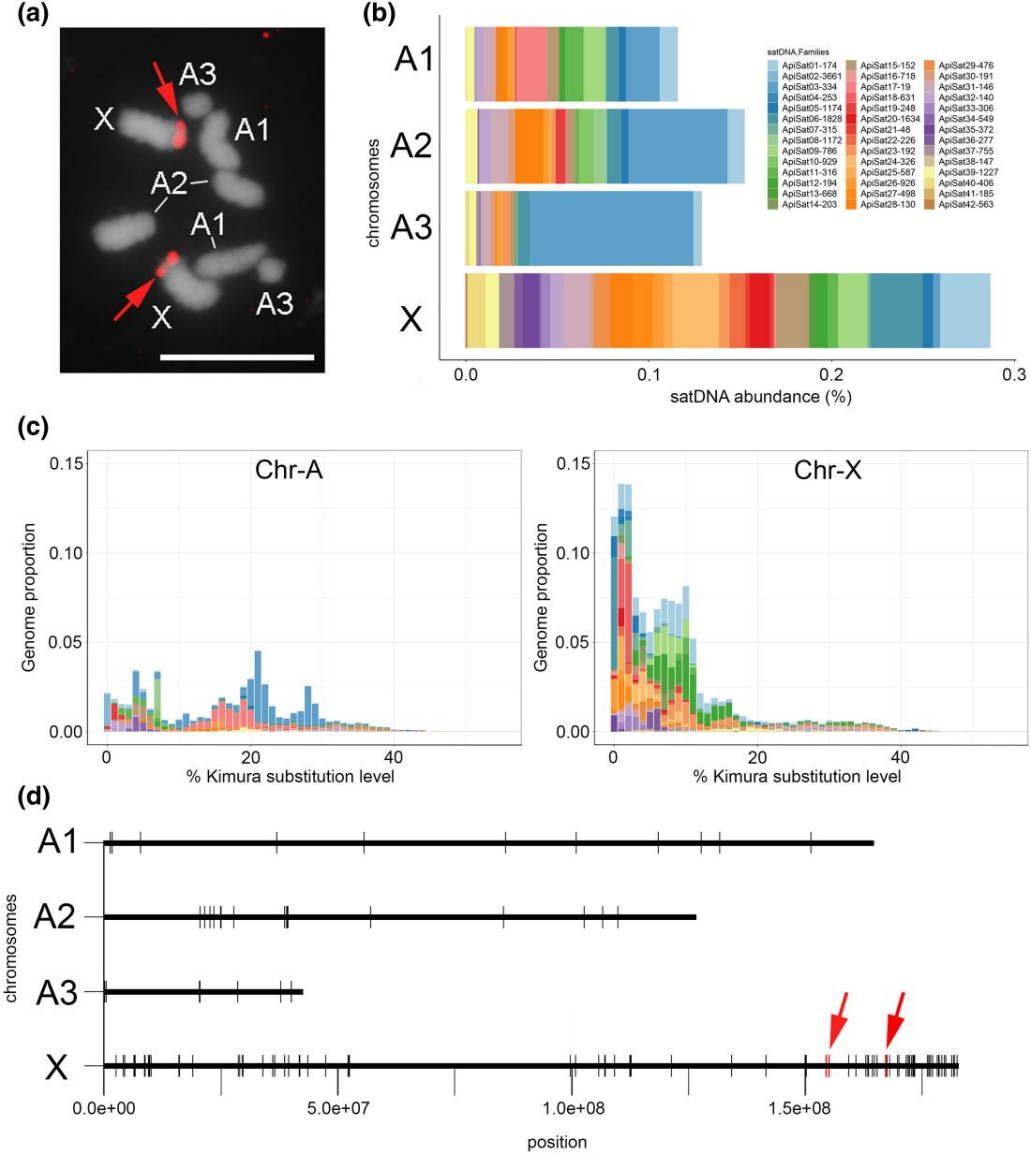
Chromosomal characteristics of satDNAs in *A. pisum*. a) Mitotic chromosomes of female embryos are visualized using DAPI staining (in gray scale), revealing a diploid number of 2*n* = 8. The X chromosomes are identified by the presence of 18S rDNA marked in red and indicated by red arrows (as described in Mandrioli et al. 1999). The remaining chromosomes are recognized based on size. Scale bar = 5 μm. b) A stacked bar plot illustrates the abundances of satDNA families on each chromosome. Each color in the plot corresponds to a specific satDNA family. c) SatDNA landscapes showing abundance and divergence for the satDNA families found on Chr-A (all autosomes) and Chr-X (chromosome X), based on the analysis of chromosomes in the genome assembly. Note the lower divergence of satDNAs from the consensus on Chr-X compared to Chr-A. d) In silico mapping with CHRISMAPP depicts the assembled chromosomes represented in megabases (Mb), in which each black line indicates the presence of satDNAs. The positions of 18S rDNA are marked in red lines (red arrows). SatDNAs are dispersed along the chromosomes, with notable accumulation observed on the Chr-X, particularly in distal regions, including the region housing the 18S rDNA.

Chromosome-specific analysis revealed varying distributions of satDNA families, with Chr-X showing distinct accumulation patterns. Chr-X showed the highest number of satDNA families, with 40 families, followed by Chr-A1 and Chr-A2, each with 27 families, and Chr-A3 with 13 families (supplementary table S3, Supplementary Material online, Fig. 3a and b). Analyzing the distribution of satDNAs across all chromosomes, we found that Chr-X had ∼0.92% of satDNAs, while Chr-A1 accounted for approximately 0.14%, Chr-A2 for 0.27%, and Chr-A3 for 0.4% (Fig. 3b; supplementary table S3, Supplementary Material online). The mean divergence across chromosomes varied, as follows: Chr-X exhibited ∼11.34%, 21% for Chr-A1 and Chr-A3, and 17.2% for Chr-A2, respectively (supplementary table S3, Supplementary Material online). The overall repeat landscape analysis (abundance vs. K2P divergence) showed a distinct pattern of more recent amplification and homogenization of satDNAs on Chr-X compared to the autosomes (Fig. 3c).

### Chromosomal Location of satDNAs

**Fig. 4.**
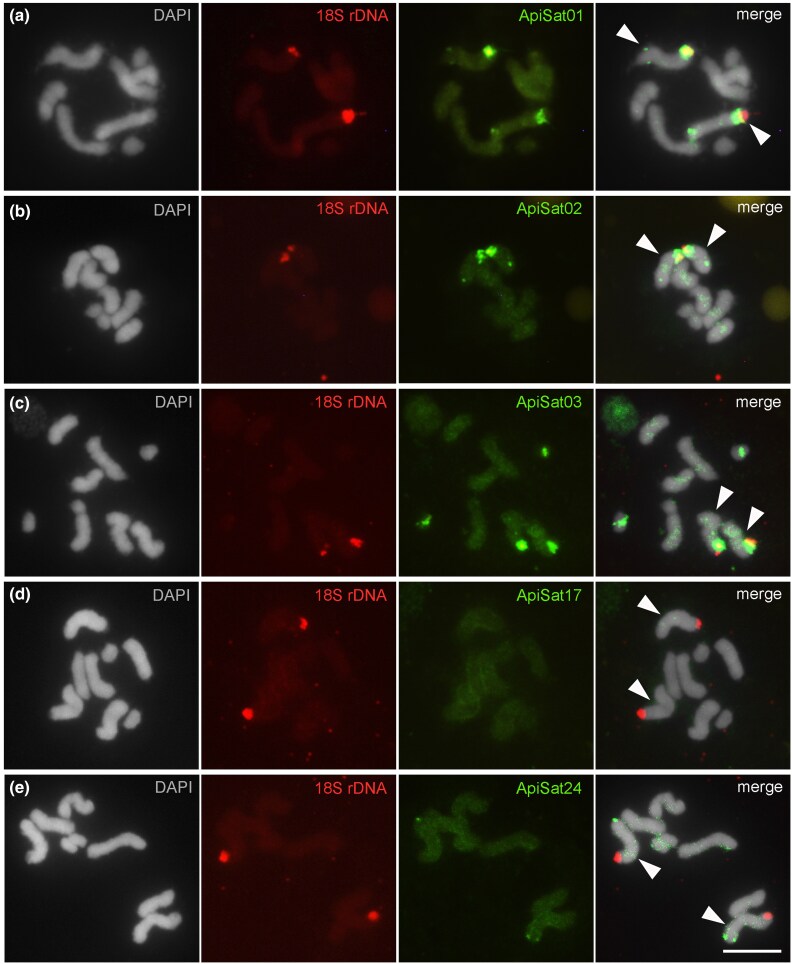
Fluorescence in situ hybridization (FISH) was performed to visualize the localization of 5 satDNAs (labeled in green) and 18S rDNA (labeled in red) on *A. pisum* chromosomes from the *Medicago sativa* biotype. The specific satDNAs analyzed and their corresponding images are: (a) ApiSat01-173, (b) ApiSat02-3661, (c) ApiSat03-334, (d) ApiSat17-19, and (e) ApiSat24-549. Chromosomes are counterstained with DAPI and shown in grayscale. The X chromosomes are identified by the presence of the rDNA cluster (see Mandrioli et al. 1999) and indicated by arrowheads. The remaining chromosomes are determined based on size differences. Bar = 5 μm.

Our computational genomic analysis using CHRomosome In Silico MAPPing (CHRISMAPP) pipeline revealed that the satDNA families were distributed along the lengths of the chromosomes (euchromatic regions), with specific accumulation at the terminus of the Chr-X, coinciding with heterochromatin regions and the presence of the 18S rDNA cluster (Fig. 3d; supplementary fig. S3, Supplementary Material online). To validate the results obtained by computational analysis, we selected 5 satDNA repeats for FISH, following rationale: (i) ApiSat01-173, due to its high abundance; (ii) ApiSat02-3661, due to both its high abundance and the lowest divergence; (iii) ApiSat03-334, due to its enrichment on autosomes and low abundance on the Chr-X; (iv) ApiSat17-19, due to it being the most highly divergent; and (v) ApiSat24-549, due to its enrichment on the Chr-X and low occurrence on autosomes (Table 2; supplementary table S3, Supplementary Material online). FISH confirmed chromosomal locations of selected satDNA families, revealing clustered and scattered distributions. The FISH mapping also revealed that the satDNAs ApiSat01-173 and ApiSat02-3661 exhibited large clusters near a terminus of Chr-X, close to the rDNA cluster. Additionally, minor subterminal clusters at the opposite terminus of Chr-X were observed, along with scattered small signals across all chromosomes (Fig. 4a and b). The FISH results for ApiSat01-173 were consistent with the analysis on assembled chromosomes. In contrast, the FISH data for ApiSat02-3661 diverged from the analysis on assembled chromosomes, probably due to the high homogenization of this repeat, which could be collapsed during the genome assembly, preventing its identification and quantification. But this is concordant with our analysis using the Illumina reads in all biotypes, as it is the second most abundant satDNA in the species. ApiSat03-334 formed a large cluster on a subterminal region of Chr-X, near the rDNA cluster, and on an interstitial region of Chr-A3, along with scattered signals across all chromosomes (Fig. 4c). ApiSat17-19 (Fig. 4d) and ApiSat24-549 exhibited a few scattered signals, with ApiSat24-549 showing a small signal on the opposite terminus of the rDNA site on Chr-X, consistent with the analysis on assembled chromosomes (Fig. 4e).

## Discussion

The content and organization of satDNAs in aphid genomes have been largely overlooked (Mathers et al. 2020; Mathers et al. 2021; Wang and Xu 2024). One reason could be the low abundance of these repetitive elements in comparison to others, like transposable elements (TEs). In *A. pisum*, satDNA content comprises about 0.83% of the genome, a small proportion compared to others insect species (Šatovic-Vukšic and Plohl 2023). This is in line with the species’ small genome size, about 540 Mb (Li et al. 2019), and could be related to low heterochromatin content, as C-positive heterochromatic blocks are confined only to the end of X chromosome and autosome 2. Low abundance of satDNAs has also been reported in certain Lepidoptera species (Cabral-de-Mello et al. 2021), which like *A. pisum*, have small genomes, low heterochromatin content, and holocentric chromosomes. Transposable elements, in contrast, show a different scenario. According to the genome assembly of *A. pisum*, LTR retrotransposons, DNA transposons, and rolling-circle *Helitrons* collectively account for ∼38% of the genome and are notably enriched in syntenic breakpoint regions, suggesting a significant role in genomic rearrangements (The International Aphid Genomics Consortium 2010; Mathers et al. 2021). In this way, based on abundance, TEs have more impact on the organization of *A. pisum* genome than do satDNAs. The disparity in abundance between TEs and satDNAs suggests that these elements may have undergone distinct phases of amplification independently.

The analysis of *A. pisum* satDNAs reveals both commonalities and unique features compared to other insect species. The enrichment of A+T content aligns with previous observations in other aphids as well as in other insects (Palomeque and Lorite 2008; Manicardi et al. 2015), that is apparently not related to the centromere organization. A notable aspect of *A. pisum* satDNAs is their exceptional length, with monomers reaching up to 3,661 bp in ApiSat02-3661, significantly longer than those typically found in other Aphididae species, which generally range from 100 to 600 bp (Bizzaro et al. 1996; Spence et al. 1998; Monti et al. 2010; Mandrioli et al. 2011). The limited size of satDNAs observed in previous studies on aphids could be influenced by the methods used for identifying the repeats (references above), such as enzymatic digestion, which generally hampers the identification of longer elements. Another notable characteristic of satDNAs among aphid genomes is the high degree of sequence similarity between satDNA monomers in some of the satDNA families. The high sequence similarity among many *A. pisum* satDNA families might suggest that this species presents high levels of nonhomologous recombination, maintaining sequence homogeneity by concerted evolution (Dover 1982), or also recent amplification of the repeats. This is contrasted by the more variable satDNAs (e.g. ApiSat03-334, ApiSat17-19, ApiSat40-406, and ApiSat42-562), which might result from their dispersion in euchromatic regions, limiting homogenization processes. The extreme case of low variability in ApiSat02-3661 underscores the impact of concerted evolution for specific satDNA families, as suggested for other species (Manicardi et al. 2015). Understanding these variations provides insight into the evolutionary dynamics of satDNAs in *A. pisum*, highlighting the interplay between chromosomal structure, sequence length, and genomic distribution. This knowledge can further contribute to our understanding of genome organization and the evolutionary pressures shaping repetitive DNA sequences. Our investigation into the satellitome across diverse biotypes of *A. pisum* sheds light on the dynamism of this genomic component at the intraspecies level, a relatively understudied aspect in satDNA biology (Pita et al. 2017; Haq et al. 2022; Montiel et al. 2022; Félix et al. 2024). Previous research has predominantly focused on interspecies comparisons (e.g. Palacios-Gimenez et al. 2020; dos Santos et al. 2021; Heitkam et al. 2021; Sales-Oliveira et al. 2024; Tunjić-Cvitanić et al. 2024). As anticipated in an intraspecies analysis, we found that the composition of satellitomes among biotypes was largely similar, particularly among those associated with plant species from the same genus (such as *Medicago*) that have more closely related genomes. Changes primarily involved quantitative differences, consistent with the library hypothesis (Fry and Salser 1977; Dover 1982). However, we observed notable instances of amplification or reduction of specific satDNAs, indicating expansion or contraction of these sequences even within more related biotypes. While our study does not reconstruct the biogeographic history of the biotypes/populations, our findings suggest that variations in satDNA abundance occurred prior to biotype specialization to different host plants as some variations were observed in the same biotype hosted by *M*. *sativa*. For example, ApiSat23-192 exhibited a higher peak of amplification in biotypes associated with *Genista* plant species. Furthermore, we observed waves of amplification following the separation of biotypes, as evidenced for *Genista* biotypes for satDNA families ApiSat20-1634 and ApiSat29-476. These changes in quantity and variability of satDNAs could contribute to biotype differentiation that along with other factors could contribute to accumulation of genetic incompatibilities (Ferree and Barbash 2009; Lower et al. 2018; Jagannathan and Yamashita 2021; Brand and Levine 2022). Fluctuations in satDNA abundance in certain biotypes are likely influenced by stochastic factors, given the generally low copy numbers of these sequences in the species’ genome, particularly among the less abundant satDNA families.

Our findings support the library hypothesis at the interspecies level (Fry and Salser 1977), as we observed variations in satDNA quantities between *A. pisum* and 3 related species. The loss of these satDNA families seems to follow a phylogenetic pattern, being present in both *Acyrthosiphon* species but absent in their sister species. Remarkably, most satDNA families have been conserved over a substantial evolutionary period, estimated at ∼32 million years, corresponding to the diversification time between these species. The long-term conservation of satDNAs across evolutionary timescales has been documented in various organisms, such as the FA-SAT in bilaterians (Chaves et al. 2017), CharSat01-52 in characiform fish (dos Santos et al. 2021), APSP in ants (Lorite et al. 2017), and PjHhal in mollusks (Petraccioli et al. 2015). In aphids, the preservation of most satDNAs across species challenges the notion of a recent origin of satDNAs in distinct species, especially considering the frequent observation of species-specific satDNAs with high sequence similarity (Manicardi et al. 2015). Previous studies relied on a limited number of satDNA families and traditional methods for satDNA analysis, which may have limited conclusions regarding satDNA evolution in aphids.

To date, comprehensive studies of the satellitome in aphid species remain limited. However, cytogenetic and molecular analyses of a few species have shown that satDNAs vary in their genomic location depending on the species, often aligning with heterochromatin distribution (Manicardi et al. 2015). This suggests that satDNAs contribute differently to chromosome organization. In *A. pisum* and other aphids, satDNAs are primarily located in the heterochromatin of the X chromosome, as seen in *Megoura viciae*, *M. persicae*, and *Rhopalosiphum padi*, each showing a distinct number of intercalary bands of satDNAs. Furthermore, enrichment in other chromosomes has been observed, such as in the subtelomeric region of all autosomes (169 bp satDNA) in *M. persicae*, highlighting the dynamic nature of this class of repetitive DNA between species. This dynamism potentially influences reproductive isolation and species differentiation (Jagannathan and Yamashita 2021; Brand and Levine 2022). In *A. pisum*, the distribution of satDNAs is also dynamic at the intragenomic level. Besides the large cluster found at a terminus of the Chr-X, there are additional clusters on Chr-X and Chr-A3, as well as dispersed signals in euchromatic regions of all chromosomes, as confirmed by FISH and CHRISMAPP. The notion of euchromatic distribution of the satDNAs in the species is based on previous data showing that the main heterochromatic blocks are on both termini of the X chromosome and autosome 2, as revealed by cytogenetic methods (Mandrioli and Borsati 2007). These findings reinforce that while satDNAs are a main component of aphid heterochromatin, not all are exclusively enriched in the main heterochromatic domains. Although, we cannot completely rule out the occurrence of small heterochromatic domains, not detected by cytogenetic techniques. The presence of satDNAs in euchromatin observed in *A. pisum* aligns with similar observations in other insects (Sproul et al. 2020; Cabral-de-Mello et al. 2023; Šatović-Vukšić and Plohl 2023; Rico-Porras et al. 2024). These sequences have been suggested to play functional roles as regulatory elements in gene expression (Brajković et al. 2012, 2018). This data underscores the need for further investigation into the functional roles of euchromatin-located satDNAs in *A. pisum*.

The autosomal chromosomes of *A. pisum* exhibit higher gene density and lower repetitive content compared to the Chr-X (Bickel et al. 2013). This aligns with the higher abundance of satDNAs on the Chr-X, which is consistent with the accumulation of other repetitive elements on this chromosome such as TEs (Mathers et al. 2021) and major rDNA clusters (Bizzaro et al. 2000). The satDNAs predominantly accumulate at the terminus of Chr-X, which corresponds to gene-poor regions, also known for TE proliferation. Mathers et al. (2021) reported that the Chr-X in *A. pisum* is larger than in *M. persicae*, potentially influenced by TE dynamics and their invasion of this chromosome. Our findings suggest that the Chr-X serves as a “fountain of youth” for satDNAs, where these sequences are continuously amplified compared to other parts of the genome, contributing to the enlargement of the Chr-X. SatDNAs on the Chr-X also exhibit lower K2P divergence compared to those on the autosomes, indicating more recent amplification and homogenization of these repeats, thereby expanding this chromosome element. These findings hint at a potential role of satDNA in contributing to the “fast-X” effect observed in aphids (Jaquiéry et al. 2018).

Overall, our investigation into the evolution of satDNAs in the pest aphid *A. pisum* revealed significant dynamics within its genome. We identified 43 distinct satDNA families characterized by A+T richness, varying monomer lengths, and divergence levels across different biotypes. Together, these families constitute 0.83% of the *A. pisum* genome, exhibiting diverse abundance and divergence patterns among biotypes. Our analysis highlighted pronounced instances of recent satDNA amplification and homogenization, particularly on specific chromosomes such as Chr-X, which harbors the largest number of satDNA families. The observed patterns of satDNA diversification in *A. pisum* suggest a dynamic interplay between amplification, homogenization, and chromosomal localization that could potentially influence genomic differentiation and isolation between biotypes. Notably, the Chr-X appears to serve as a focal point for ongoing satDNA amplification, contributing to its enlargement relative to autosomes. The lower divergence of satDNAs on Chr-X compared to autosomes implies recent amplification events followed by homogenization processes. This phenomenon may contribute to the “fast-X” effect observed in aphids, where genomic elements on Chr-X evolve at an accelerated pace compared to autosomes. Understanding these dynamics provides insights into the evolutionary mechanisms shaping satDNA diversity and distribution within the *A. pisum* genome.

## Materials and Methods

### Genomic Datasets

The datasets for *A. pisum* included 16 individual genomes including 11 biotypes associated with distinct plants. For each biotype we analyzed 1 sample, except for *M. sativa* of which 6 samples were studied. The average sample size included 25 pooled individuals per biotype, covering distinct clones. Each sample derives from individuals associated with identical plant species, plants belonging to the same genus, and distinct genera. Furthermore, we selected 3 sister species as outgroups of *A. pisum* (*Acyrthosiphon caraganae*, *A. craccivora*, and *M. persicae*) based on their taxonomic relationship as delineated by the phylogenetic hypothesis proposed by Mathers et al. (2021). We retrieved the sequencing libraries from NCBI-SRA (www.ncbi.nlm.nih.gov/sra). Detailed information about the samples could be consulted by accessing the accession numbers indicated in Table 1. These libraries were subsequently used for the prospecting, characterizing, and analysis of satDNAs.

### satDNA Library Analysis: Identification of satDNAs and Calculation of Abundance and Divergence

At the outset, a random sampling of 10 million paired-end reads per genome was performed using the Galaxy online platform (Afgan et al. 2016) on the public server www.usegalaxy.org. The raw reads obtained from sampling were subjected to preprocessing procedures, including trimming, filtering, and read interlacing, using the default parameters on the Galaxy online platform. The quality cutoff value of 10 and the criterion of 95% above cutoff were implemented in our analysis. To identify the satDNA families, we employed the TAndem REpeat ANalyzer, TAREAN (Novák et al. 2017), on each of the 16 *A. pisum* samples. To enhance the reliability of satDNAs identification, we exclusively considered satDNAs classified as “high confidence” by TAREAN, provided they were identified at least once in one of the biotypes. Only those satDNAs meeting these criteria were subjected to subsequent analyses.

To perform the homology analysis, the consensus sequences (monomers) of each satDNA family were compared all-to-all using Geneious 4.8.5 software (http://www.geneious.com). We classified the satDNA families following the framework proposed by Ruiz-Ruano et al. (2016). The outcome of this analysis was used to construct the satDNA library specific to *A. pisum*. This specific library generated for *A. pisum* thus served as a customized reference, enabling the evaluation and comparative analysis of the relative abundance and sequence divergence of satDNA families across the 11 biotypes (16 genomes) and the 3 closely related aphid species using the short read sequenced genomes. This analysis was performed using RepeatMasker Open-4.0 software (http://www.repeatmasker.org), considering the number of nucleotides analyzed in each sample for abundance calculation. A satDNA family was considered to be present in a genome only when at least 2 monomers were present, based on the abundance percentage and the monomer sizes. The script calcDivergenceFromAlign.pl was used to estimate the divergence average based on Kimura2-parameter distances (Kimura 1980) for each satDNA family. The satDNAs were named ApiSat and subsequently numbered in a descending order of abundance, following the abundance of the *P. sativum* biotype. To check the similarity of the satDNAs with previously identified sequences, we ran a BLAST analysis using a monomer of each satDNA family against nucleotide database from NCBI or sequence masking using Repbase (Bao et al. 2015) datasets. Both analyses were performed online.

We also used the generated satDNA library specific to *A. pisum* to calculate the abundance and divergence of satDNA families between the assembled chromosomes (JIC1 v1 genome assembly), employing RepeatMasker. In the assembled genome JIC1 v1, the scaffold_1 corresponds to Chr-X, scaffold_2 is the Chr-A1, scaffold_3 is the Chr-A2, and scaffold_4 is the Chr-A3. This genome assembly was done in an isolate collected in *Lathyrus odoratus* from Norwich in 2005 that were reared under controlled conditions in the lab. It was based on high-coverage Nanopore long-read data. The pseudomolecules were assembled with Hi-C chromatin conformation capture data and 10X Genomics Chromium linked reads (Mathers et al. 2021).

### Chromosomal Mapping of satDNAs by Fluorescence In Situ Hybridization and CHRISMAPP Pipeline

For the physical chromosomal characterization of satDNAs, we selected 5 families based on their abundance, divergence, and genome organization as revealed by computational analysis (see details in results section). The probes for each satDNA family were synthesized with biotin at the 3′ end (supplementary table S1, Supplementary Material online). Additionally, a heterologous probe for 18S rDNA was amplified from the genome of *Diatraea saccharalis* (Lepidoptera) using the primers Sca18SF (5′ CCC CGT AAT CGGAAT GAG TA) and Sca18SR (5′ GAG GTT TCC CGT GTTGAG TC) (Cabral-de-Mello et al. 2010) and labeled with digoxigenin-11-dUTP (Roche, Mannheim, Germany) by PCR. This aimed for the clear identification of Chr-X, which has a similar size to Chr-A1, but harbors the rDNA cluster. The FISH procedure followed the protocol outlined by Cabral-de-Mello and Marec (2021) using as source chromosomes from a population collected in alfalfa (*M. sativa*) field situated ∼800 m apart in Ithaca, NY (Site 1:42°26′52.9″N, 76°27′07.5″W; Site 2:42°26′46.0″N, 76°26′25.5″W) (Chung et al. 2020). Probes labeled with biotin were detected using Alexa-Fluor 488-conjugated streptavidin from Invitrogen (CA, USA), while the digoxigenin-labeled probe was detected using antidigoxigenin-rhodamine (Roche). Chromosomes were stained with 4′,6-diamidino-2-phenylindole (DAPI), and the slides were mounted using VECTASHIELD from Vector (CA, USA). The results were analyzed using an Olympus BX61 epifluorescence microscope, and photographs were captured using a DP70 cooled digital camera. The images were merged and optimized for brightness and contrast using Adobe Photoshop CS2 software.

We determined the chromosomal locations of each satDNA and the 18S rDNA (accession number U27819.1) through the CHRISMAPP pipeline, following Rico-Porras et al. (2024) on the 4 scaffolds corresponding to chromosomes on the JIC1 v1assembled genome (Mathers et al. 2021) available on AphidBase (https://bipaa.genouest.org/is/aphidbase/).

## Supplementary Material

evaf104_Supplementary_Data

## Data Availability

All data analyzed in this work is indicated on Table 1 with their respective accession numbers form NCBI.
