# Supplementary material for: Exploring the Satellitome of the Pest Aphid Acyrthosiphon pisum (Hemiptera, Aphididae): Insights Into Genome Organization and Intraspecies Evolution
Source: Genome Biol Evol. 2025 Jul 10;17(7):evaf104. doi: 10.1093/gbe/evaf104 (PMC12241859; doi:10.1093/gbe/evaf104)
Supplement: evaf104_Supplementary_Data [file evaf104_supplementary_data.zip › R2_Supp_tables/R2_Supplementary_Table_1_probes.docx]

| **SatDNA** | **Probe sequence** | **3’ marker** |
| --- | --- | --- |
| ApisSat01-173 | CACCTGTGGCGGTGGAATCCCGTCCCGTTTCTCCAATACTTAATATTTTTTCATAATTTTTTTACCAAAAGATAGGGTTTAATATACTTAAGTTGACGTG | Biotin |
| ApisSat02-3661 | CGCCATAACTTCCTCTCCCTTCCCCTTCATATTCAGCAATTAATTGCATCGCTCGAACTTTGGGTGCACCAGTTAATGTCCCCATATTCATACAAGAGGA | Biotin |
| ApisSat03-334 | CGATCTAAACGTTTATAGAAAAAAATTTTGTAAATGTTATTTTTTTTTGAACTAAGGTGTTGACGACATACCTGTTGATGGCTTTTTTAATACAAAATTT | Biotin |
| ApisSat17-19 | TACCTAGAGCTGATGGTAGTACCTAGAGCTGATGGTAG | Biotin |
| ApisSat24-326 | TGGAAATAGGTCTCTAAATTGTATATTACGTGTATTATTTTGTTATTGTATAATTCTAACACCCAGGTTAAACTATTTCTAGGAGTCAATACCACCGTGT | Biotin |

**Supplementary Table 2.** Oligonucleotides used as probes for Fluorescence in situ Hybridization on chromosomes of *Acyrthosiphon pisum*.
