# Supplementary material for: Exploring the Satellitome of the Pest Aphid Acyrthosiphon pisum (Hemiptera, Aphididae): Insights Into Genome Organization and Intraspecies Evolution
Source: Genome Biol Evol. 2025 Jul 10;17(7):evaf104. doi: 10.1093/gbe/evaf104 (PMC12241859; doi:10.1093/gbe/evaf104)
Supplement: evaf104_Supplementary_Data [file evaf104_supplementary_data.zip › R2_Supp_tables/R2_Supplementary_Table_3_satDNAs_chr.docx]

**Supplementary Table 4.** SatDNA chromosome (Chr) proportions and divergences on the chromosomes of assembled genome of *Acyrthosiphon pisum*.

| **SatDNA** | **Chromosomes** | | | | | | | |
| --- | --- | --- | --- | --- | --- | --- | --- | --- |
|  | **Chr-A1** | | **Chr-A2** | | **Chr-3** | | **Chr-X** | |
|  | **K2P div (%)** | **Chr proportion** | **K2P div (%)** | **Chr proportion** | **K2P div (%)** | **Chr proportion** | **K2P div (%)** | **Chr proportion** |
| ApiSat01-174 | 6.35 | 0.0115513 | 21.24 | 0.003285571 | 18.73 | 0.00468827 | 7.95 | 0.143047075 |
| ApiSat02-3661 | 0.22 | 0.01559039 | - | 0 | - | 0 | - | 0 |
| ApiSat03-334 | 23.82 | 0.01754854 | 25.85 | 0.079521895 | 16.24 | 0.32443471 | 21.22 | 0.001492003 |
| ApiSat04-253 | - | 0 | - | 0 | - | 0 | 1.98 | 0.005235982 |
| ApiSat05-1174 | 26.84 | 0.00239342 | 23.33 | 0.005827046 |  | 0 | 7.15 | 0.033273362 |
| ApiSat06-1828 | 23.32 | 0.00609329 | 20.44 | 0.00725277 | 25.77 | 0.00850528 | 3.86 | 0.049337461 |
| ApiSat07-315 | 28.93 | 0.00012767 | 22.14 | 0.000102007 |  | 0 | 4.24 | 0.03347993 |
| ApiSat08-1172 | - | 0 | 7.48 | 0.017625054 | - | 0 | 11.92 | 5.26009E-05 |
| ApiSat09-786 | - | 0 | - | 0 | - | 0 | 8.04 | 0.048360508 |
| ApiSat10-929 | - | 0 | 8.41 | 0.008929163 | - | 0 | 35.09 | 0.000291497 |
| ApiSat11-316 | 28.53 | 0.00035017 | 6.94 | 0.020819689 | - | 0 | 13.66 | 0.015166595 |
| ApiSat12-194 | 34.35 | 0.00035868 | 14.90 | 0.006153626 | - | 0 | 11.03 | 0.107159009 |
| ApiSat13-668 | - | 0 | - | 0 | - | 0 | 8.68 | 0.021134607 |
| ApiSat14-203 | 26.74 | 0.00013496 | 5.85 | 7.67029E-05 | 18.65 | 0.00055807 | 5.81 | 0.026765096 |
| ApiSat15-152 | 19.32 | 0.02122166 | 20.87 | 0.022554597 | 27.83 | 0.00674866 | 20.98 | 0.021211864 |
| ApiSat16-718 | 33.48 | 0.00037509 | - | 0 | - | 0 | 3.39 | 0.0089038 |
| ApiSat17-19 | 15.77 | 0.03858417 | 28.78 | 0.001035093 | - | 0 | - | 0 |
| ApiSat18-631 | - | 0 | 17.81 | 0.000702187 | - | 0 | 2.16 | 0.057135545 |
| ApiSat19-248 | 32.74 | 0.00037813 | 6.56 | 0.022228807 | - | 0 | 24.44 | 0.001805965 |
| ApiSat20-1634 | - | 0 | - | 0 | - | 0 | 3.03 | 0.015646579 |
| ApiSat21-48 | - | 0 | - | 0 | - | 0 | 7.11 | 0.004210264 |
| ApiSat22-226 | 23.10 | 0.00021825 | 20.98 | 5.69341E-05 | - | 0 | 2.86 | 0.011336592 |
| ApiSat23-192 | 6.78 | 0.00027782 | 16.79 | 0.001602853 | 14.96 | 0.02836979 | 10.99 | 0.041690056 |
| ApiSat24-326 | 28.54 | 0.00018299 |  | 0 |  | 0 | 5.18 | 0.03415114 |
| ApiSat25-587 | 31.86 | 0.00143837 | 36.62 | 0.003858866 | 29.53 | 0.00284687 | 9.79 | 0.024769549 |
| ApiSat26-926 | 21.48 | 0.00290773 | 19.67 | 0.002602362 | 20.50 | 0.00305408 | 6.62 | 0.022286895 |
| ApiSat27-498 | 17.72 | 8.9974E-05 | - | 0 | - | 0 | 2.32 | 0.019719861 |
| ApiSat28-130 | 11.30 | 0.0069371 | 11.95 | 0.007420409 | - | 0 | 10.46 | 0.012991329 |
| ApiSat29-476 | - | 0 | 22.04 | 0.000177128 | - | 0 | 5.28 | 0.014438949 |
| ApiSat30-191 | - | 0 | 4.29 | 0.005527351 | 20.18 | 0.00068523 | 5.62 | 0.00880079 |
| ApiSat31-146 | 29.21 | 0.00835905 | 28.21 | 0.007946259 | 30.69 | 0.01876484 | 25.64 | 0.02496461 |
| ApiSat32-140 | 14.16 | 0.00276183 | 4.48 | 0.018478274 | 9.67 | 0.00089244 | 11.34 | 0.019140704 |
| ApiSat33-306 | 22.60 | 0.00028998 | 20.45 | 5.13988E-05 | - | 0 | 2.96 | 0.014950165 |
| ApiSat34-549 | - | 0 | 4.88 | 0.017882048 | - | 0 | 28.97 | 0.000751207 |
| ApiSat35-372 | 18.05 | 3.4652E-05 | 22.58 | 0.000374816 | 24.36 | 0.00029905 | 1.89 | 0.006972908 |
| ApiSat36-277 | - | 0 | - | 0 | - | 0 | 6.14 | 0.022997556 |
| ApiSat37-755 | 6.95 | 2.9181E-05 | - | 0 | - | 0 | 14.54 | 0.010497169 |
| ApiSat38-147 | - | 0 | - | 0 | - | 0 | 11.28 | 0.008632028 |
| ApiSat39-1227 | 23.17 | 0.00445917 | 21.01 | 0.006427226 | 20.60 | 0.00444338 | 23.28 | 0.015976978 |
| ApiSat40-406 | 26.60 | 0.00011611 | - | 0 | - | 0 | 18.50 | 0.009043521 |
| ApiSat41-185 | - | 0 | - | 0 | - | 0 | 27.65 | 0.000150132 |
| ApiSat42-563 | - | 0 | - | 0 | - | 0 | 20.69 | 0.001553371 |
| ApiSat43-174 | - | 0 | - | 0 | - | 0 | - | 0 |
| Total abundance |  | 0.14280971 |  | 0.268520133 |  | 0.404290667 |  | 0.919525251 |
| Mean divergence | 21.55296 |  | 17.20556 |  | 21.36231 |  | 11.3435 |  |
