# Supplementary material for: Exploring the Satellitome of the Pest Aphid Acyrthosiphon pisum (Hemiptera, Aphididae): Insights Into Genome Organization and Intraspecies Evolution
Source: Genome Biol Evol. 2025 Jul 10;17(7):evaf104. doi: 10.1093/gbe/evaf104 (PMC12241859; doi:10.1093/gbe/evaf104)

Supplementary figure 1. The satDNA library found in *Acyrthosiphon pisum* associated with *Medicago sativa* is visually represented through stacked bar plots. These plots offer a comparative view of the proportions of each satDNA across different populations from same biotype. Each color within the plot corresponds to a specific population, enabling visual differentiation and analysis.

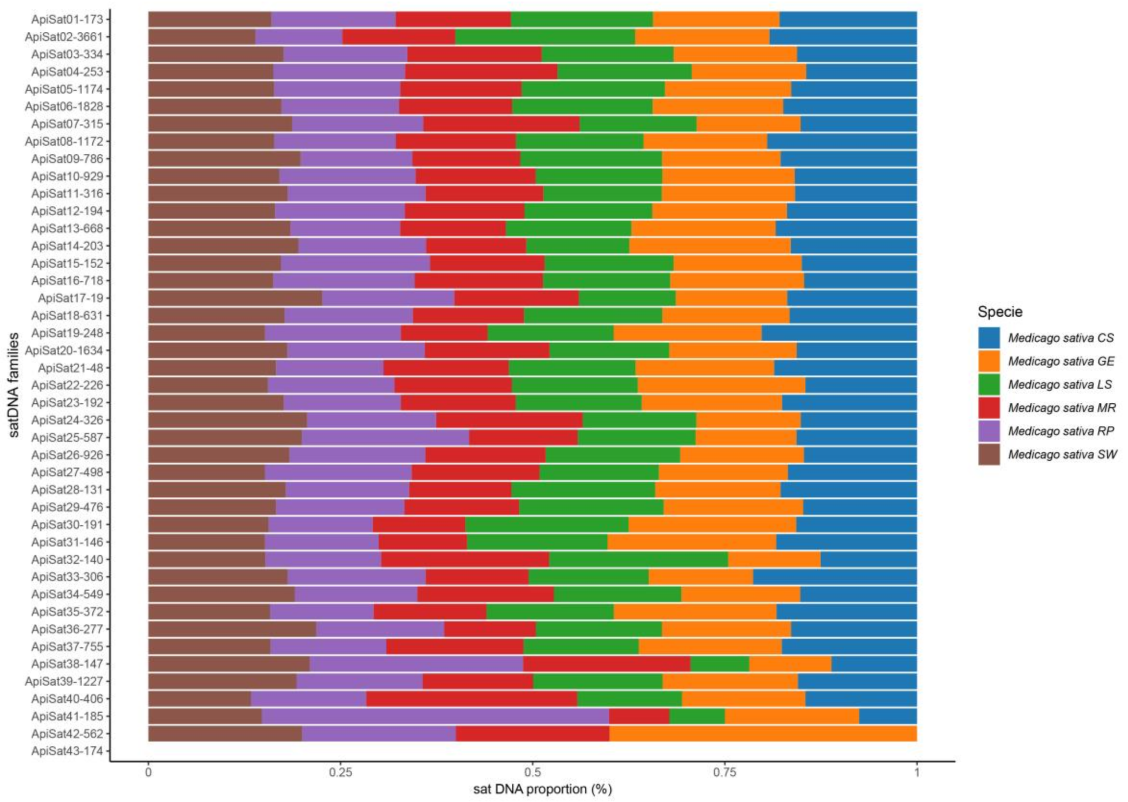

Supplement: evaf104_Supplementary_Data [file evaf104_supplementary_data.zip › R2_Supp_Figures/R2_Supp_Figure_1_medicago.pdf]
