# Supplementary material for: Exploring the Satellitome of the Pest Aphid Acyrthosiphon pisum (Hemiptera, Aphididae): Insights Into Genome Organization and Intraspecies Evolution
Source: Genome Biol Evol. 2025 Jul 10;17(7):evaf104. doi: 10.1093/gbe/evaf104 (PMC12241859; doi:10.1093/gbe/evaf104)
Supplement: evaf104_Supplementary_Data [file evaf104_supplementary_data.zip › R2_Supp_Figures/R2_Supp_Figure_2_landscapes_general.pdf]

Supplementary figure 2. Landscape plots illustrating the abundance versus K2P divergence values patterns for the 43 satDNA families across the 16 populations of *Acytosiphon pisum*.

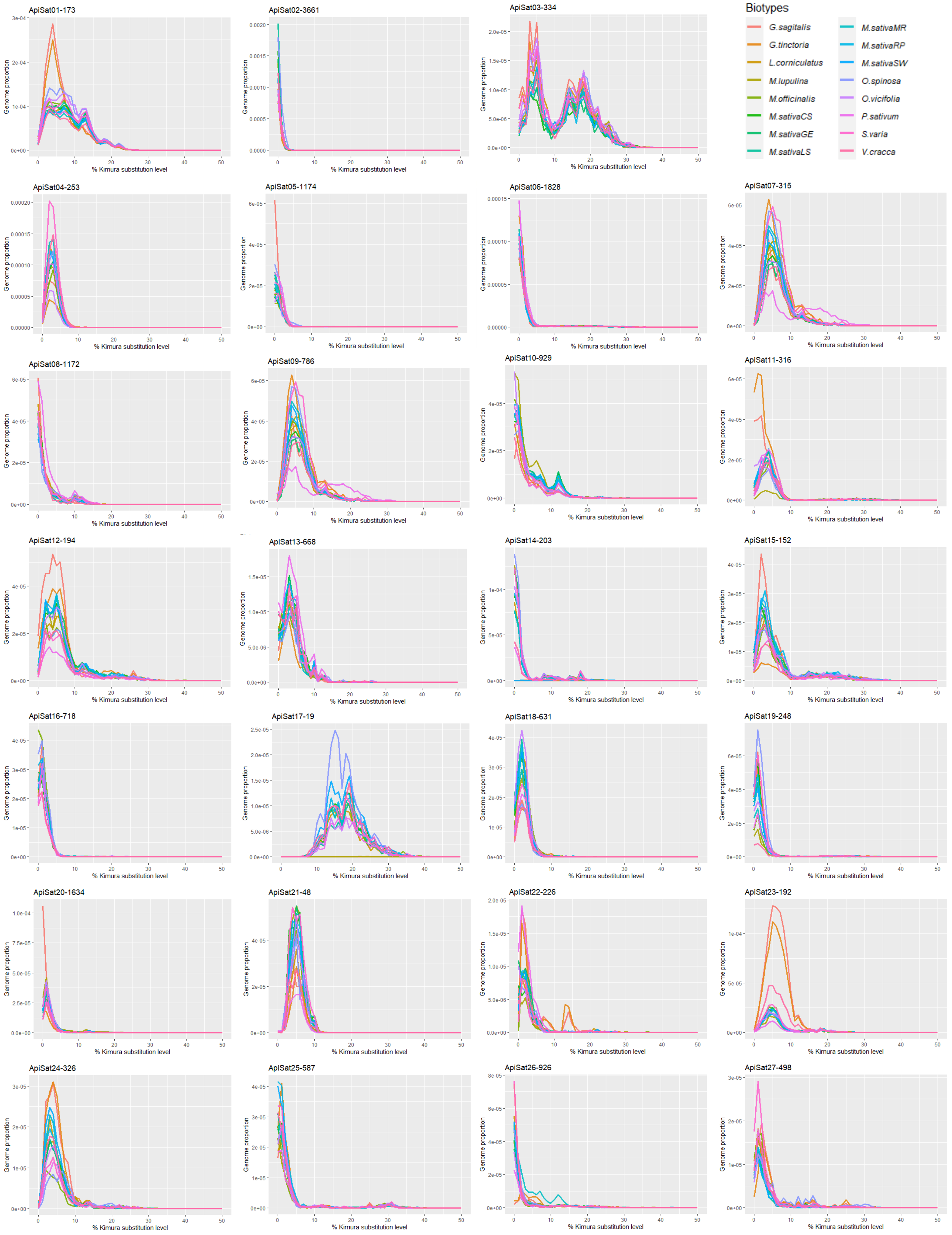

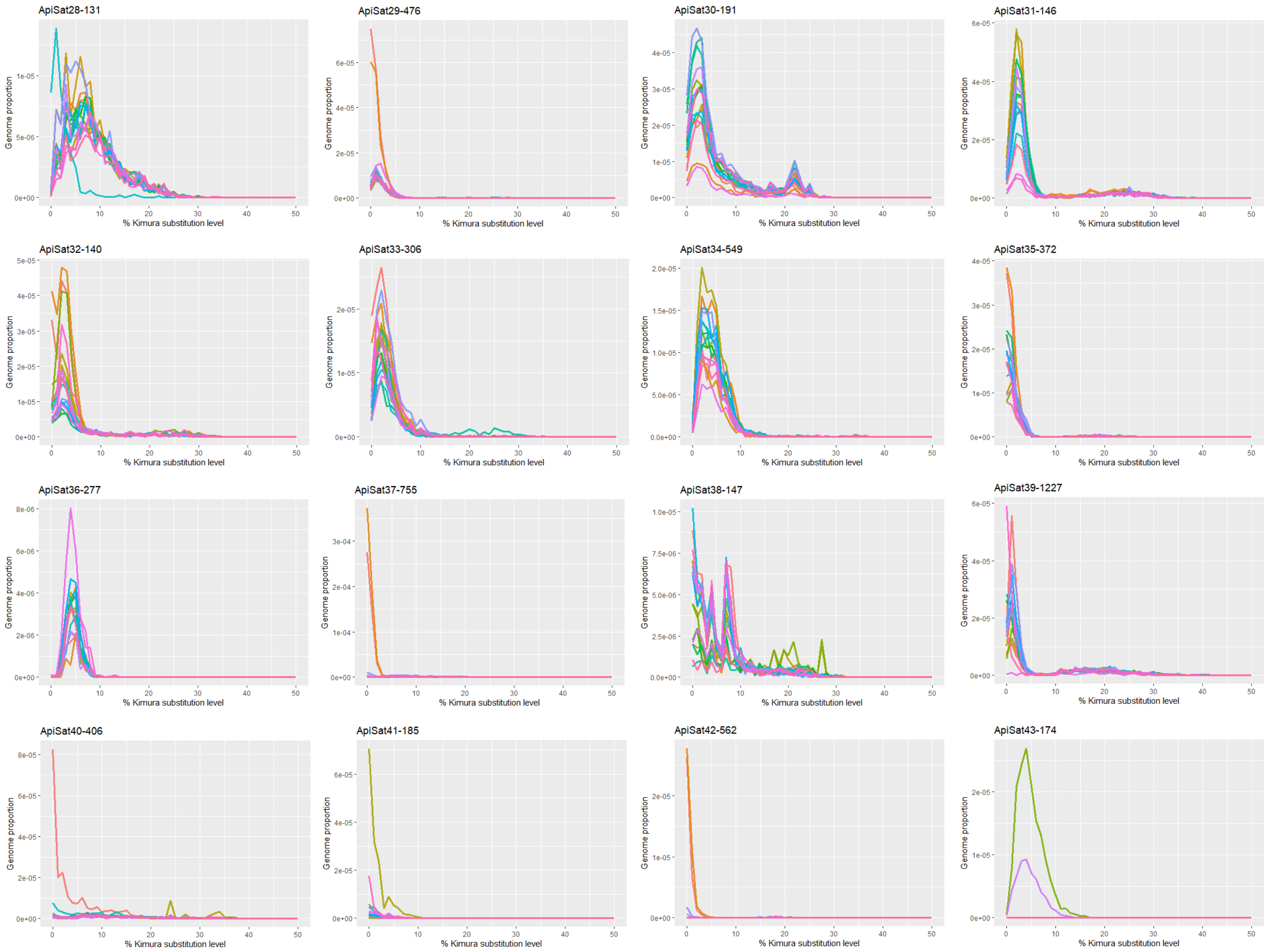
ApiSat28-131
ApiSat29-476
ApiSat30-191
ApiSat31-146
ApiSat32-140
ApiSat33-306
ApiSat34-549
ApiSat35-372
ApiSat36-277
ApiSat37-755
ApiSat38-147
ApiSat39-1227
ApiSat40-406
ApiSat41-185
ApiSat42-562
ApiSat43-174
Genome proportion
% Kimura substitution level
