# Supplementary figures and images for: Exploring the Satellitome of the Pest Aphid Acyrthosiphon pisum (Hemiptera, Aphididae): Insights Into Genome Organization and Intraspecies Evolution
Source: Genome Biol Evol. 2025 Jul 10;17(7):evaf104. doi: 10.1093/gbe/evaf104 (PMC12241859; doi:10.1093/gbe/evaf104)

**Supplementary Figure 3.** Individual CHRISMAPP chromosome mapping of the satDNAs families.

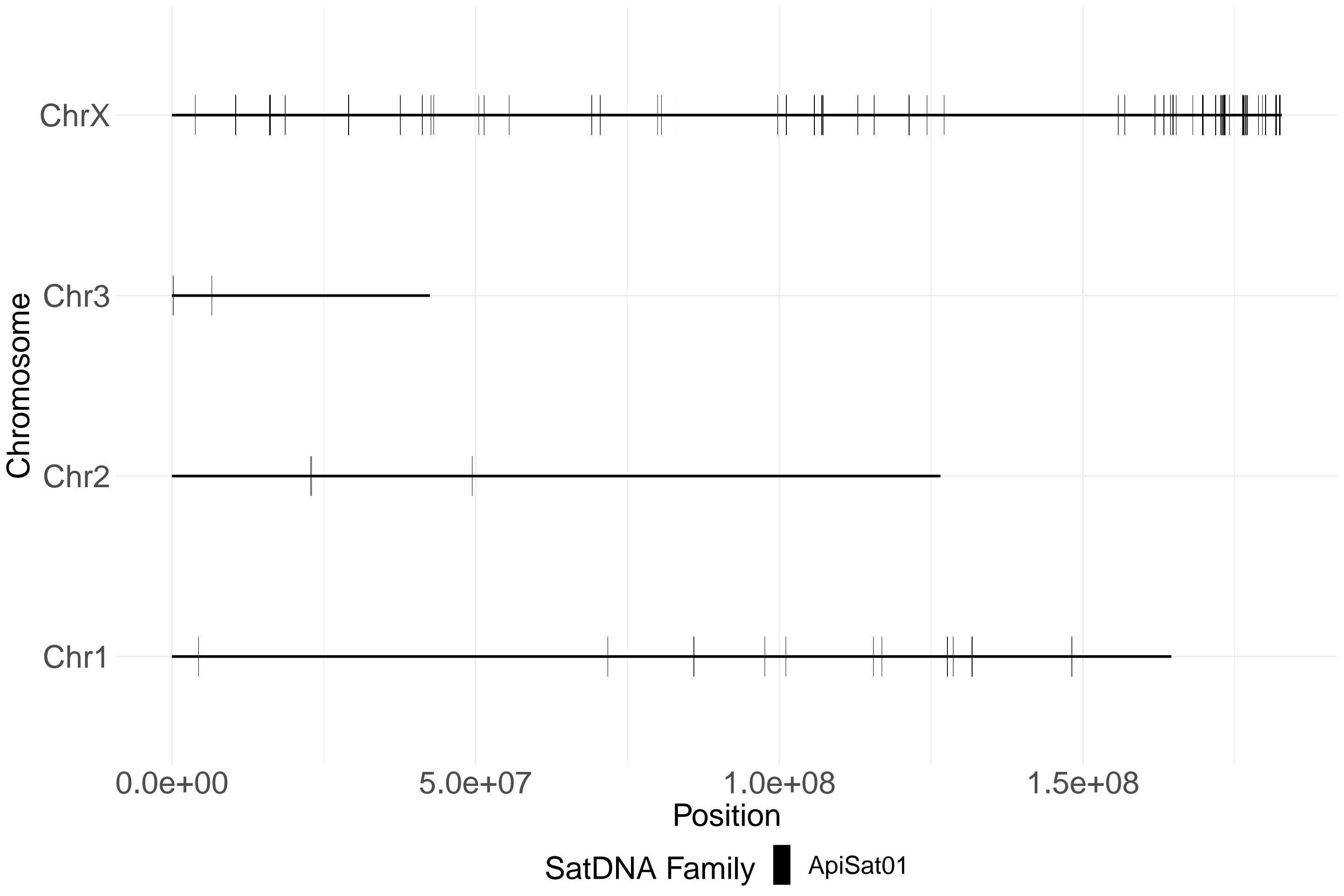

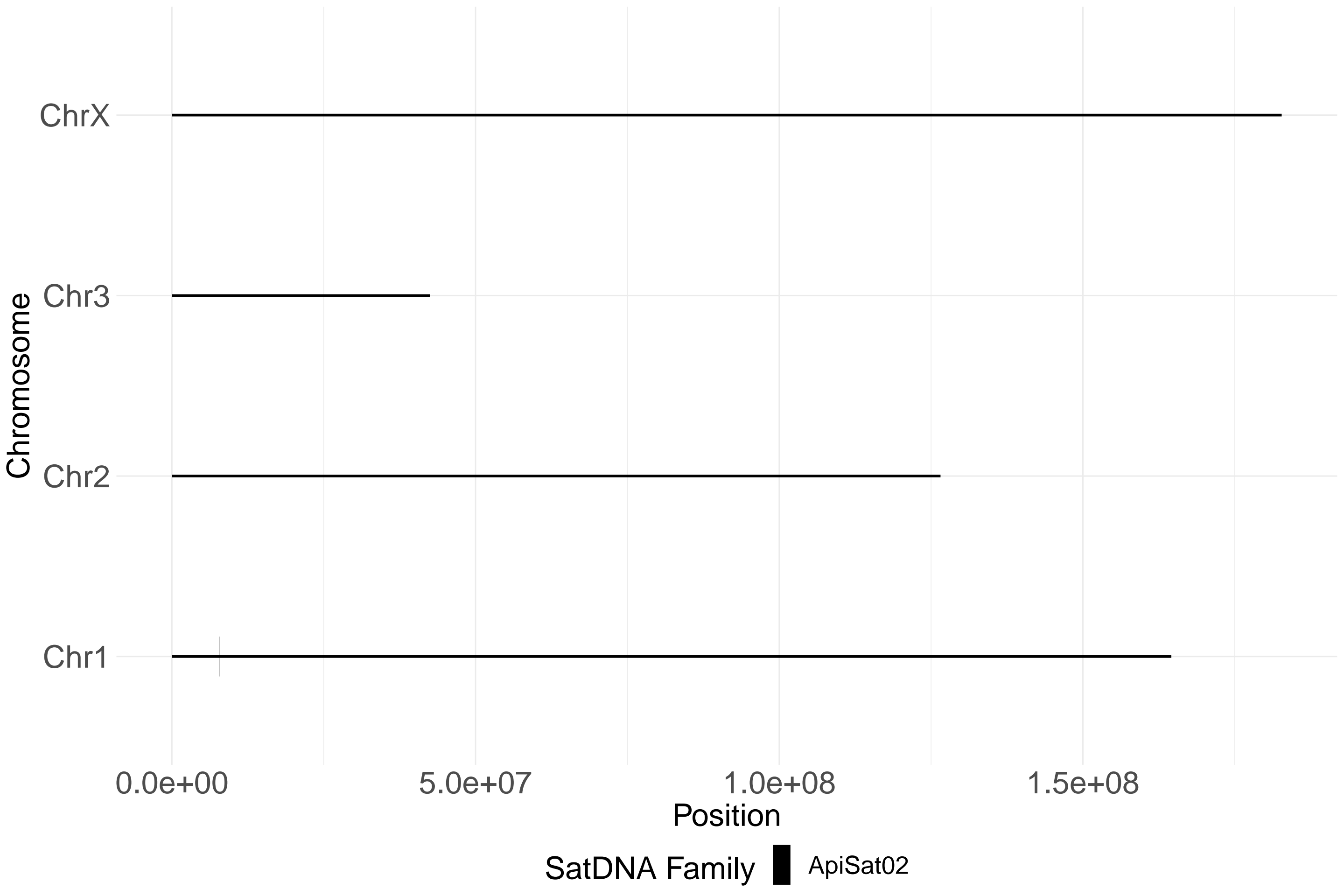

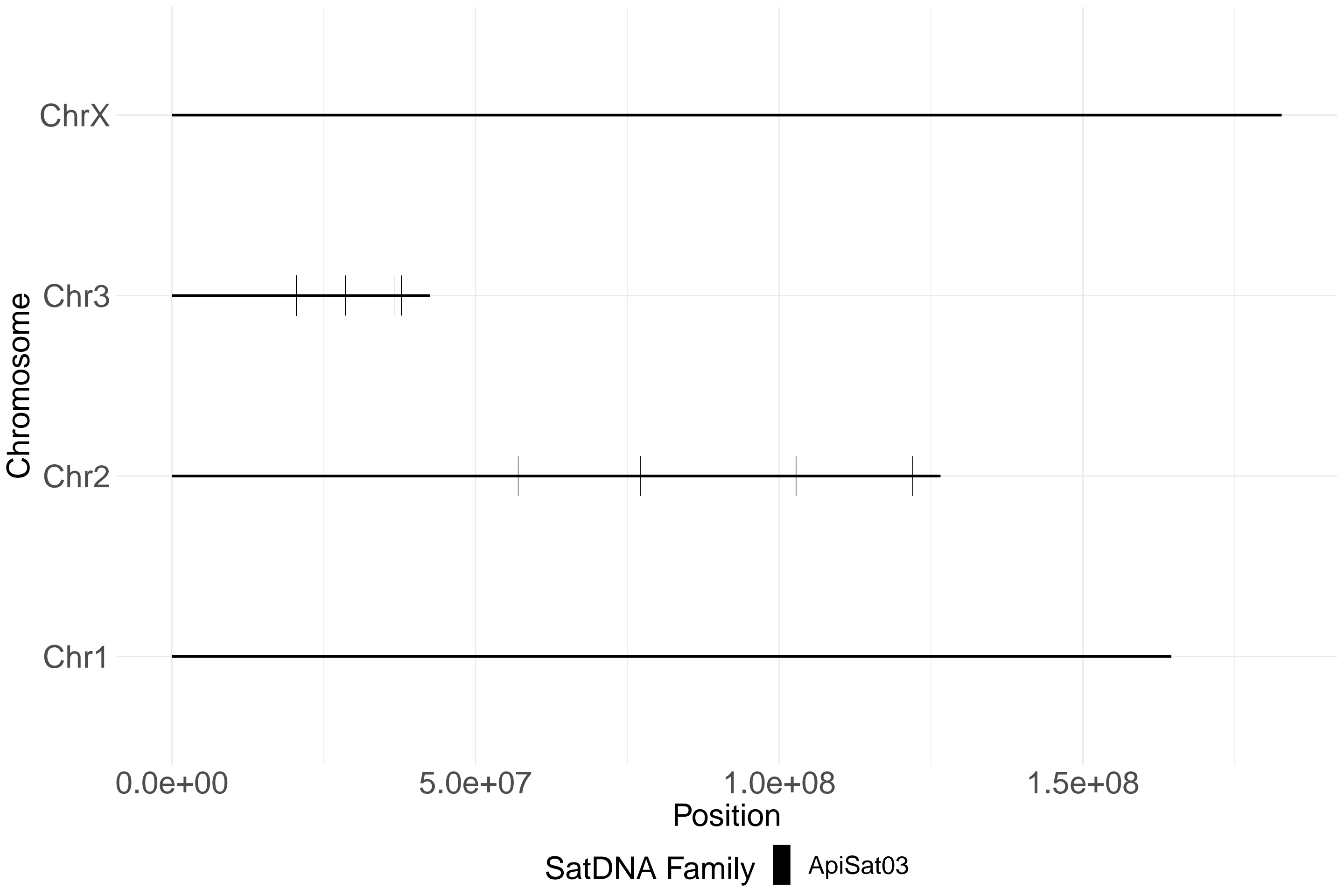

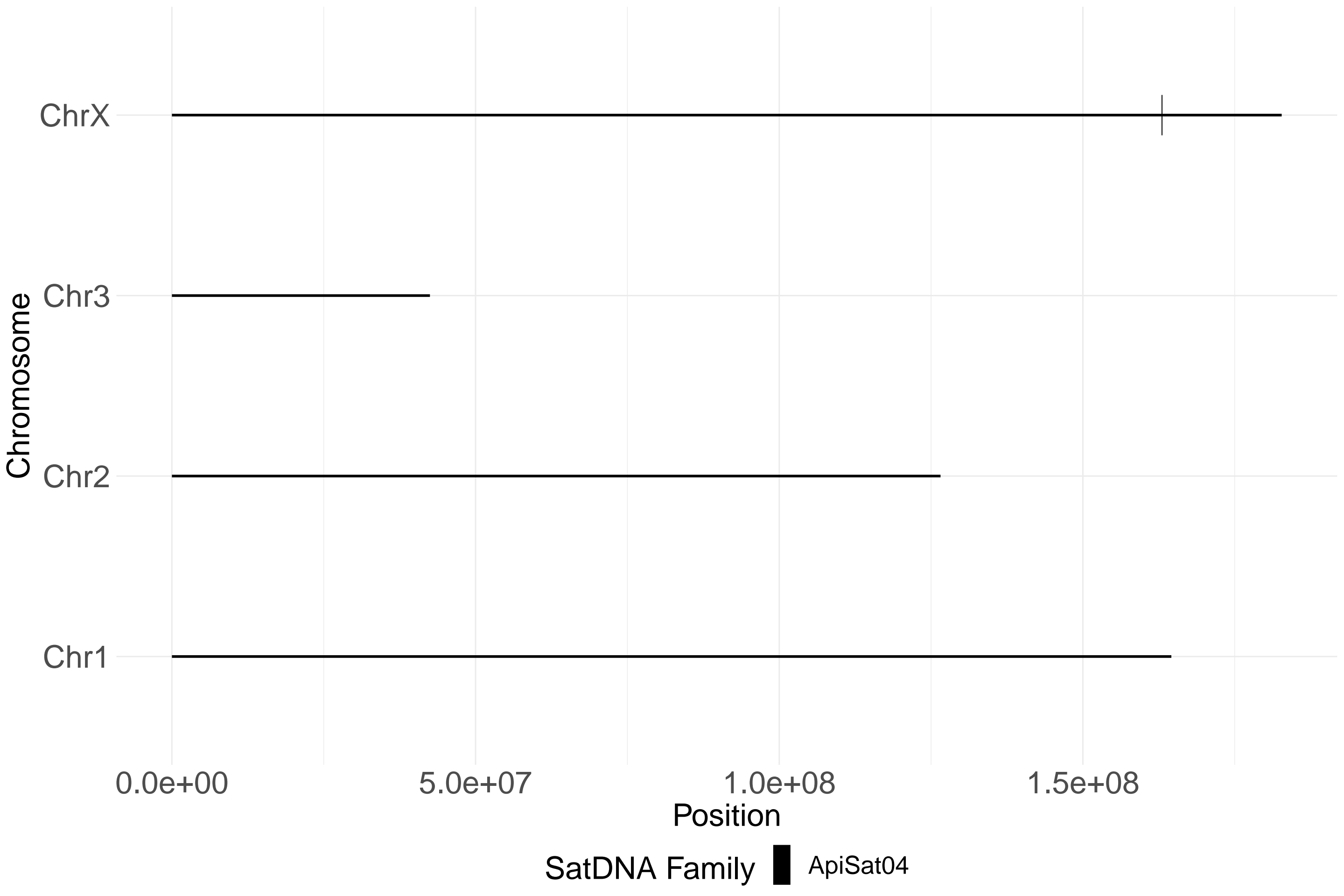

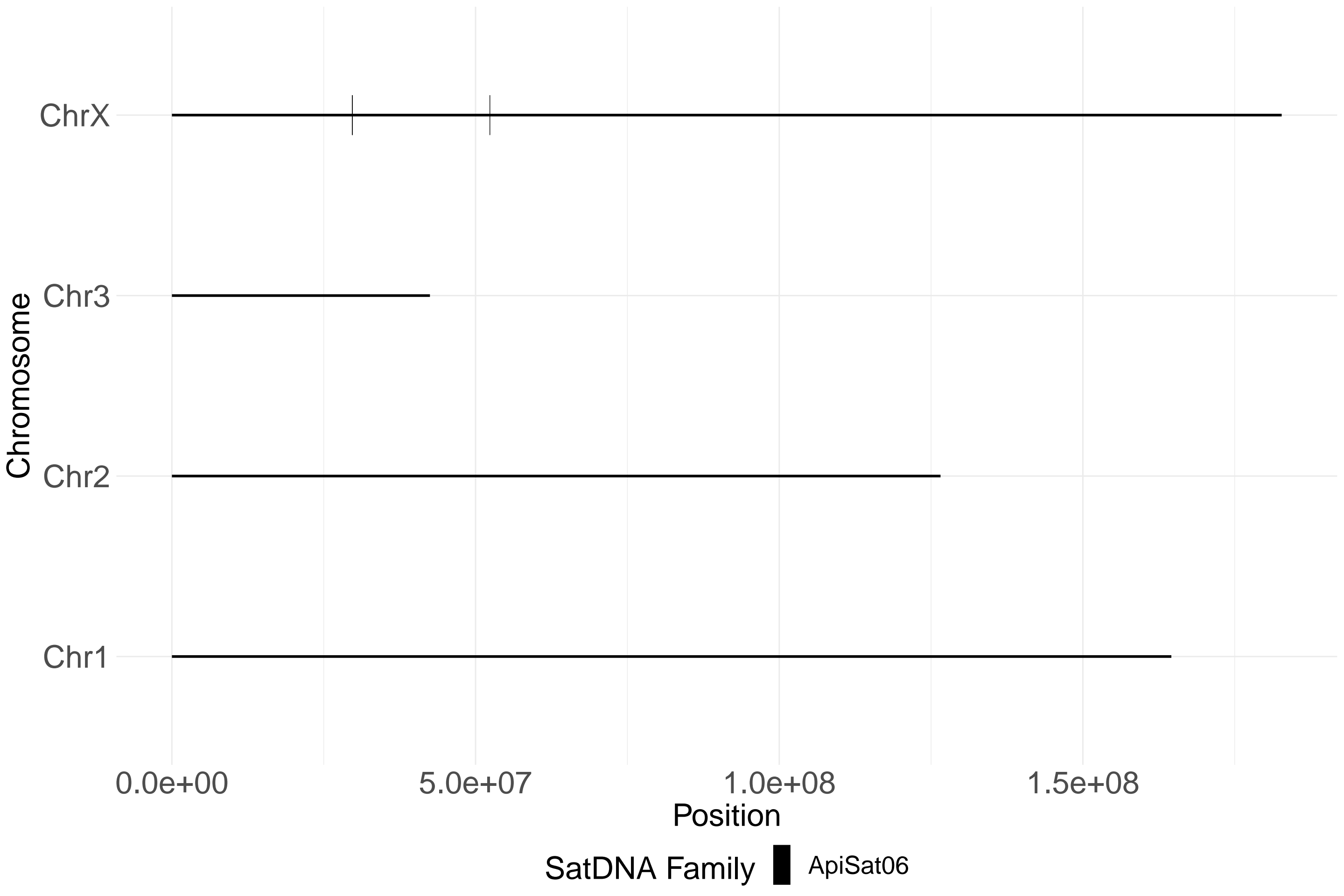

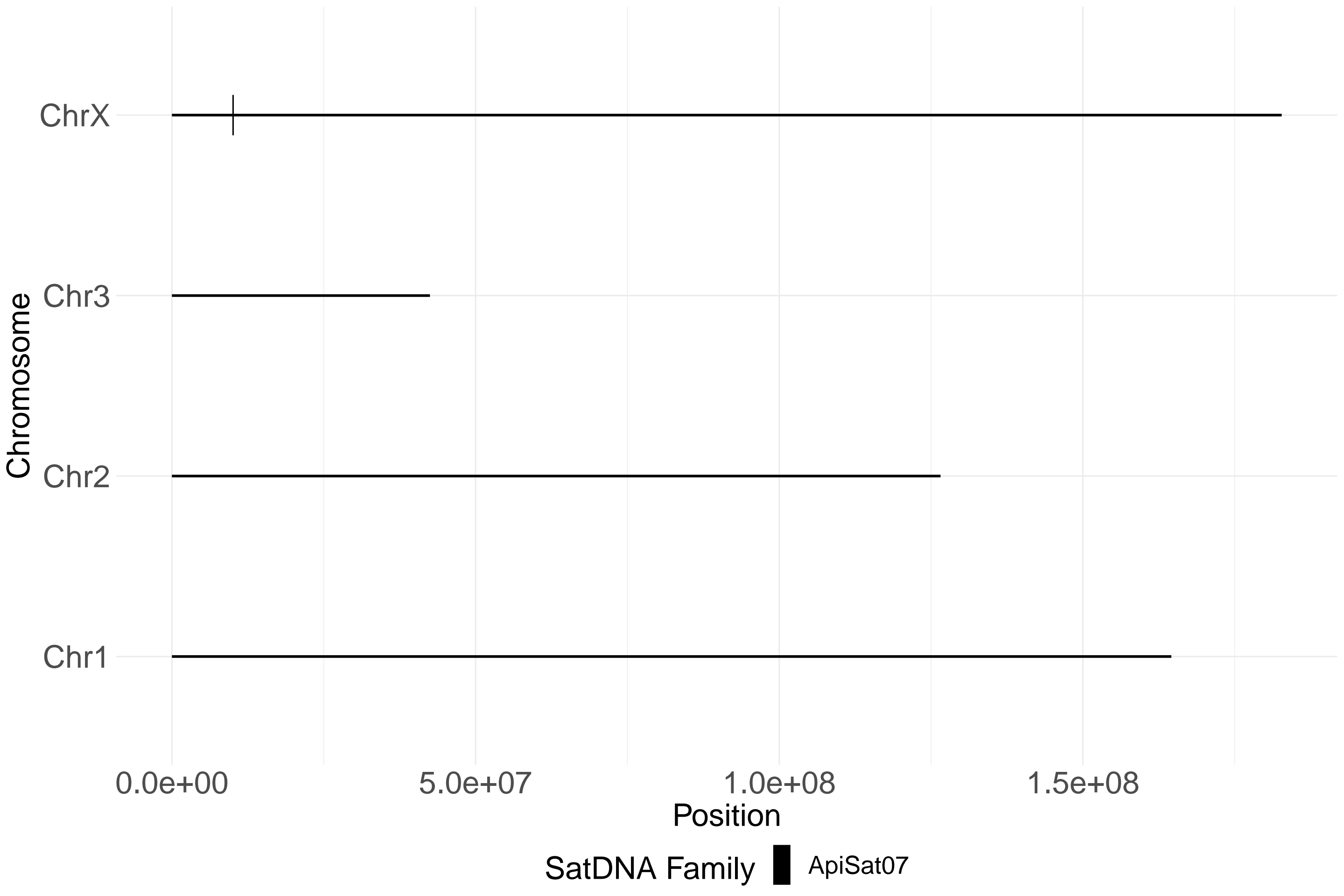

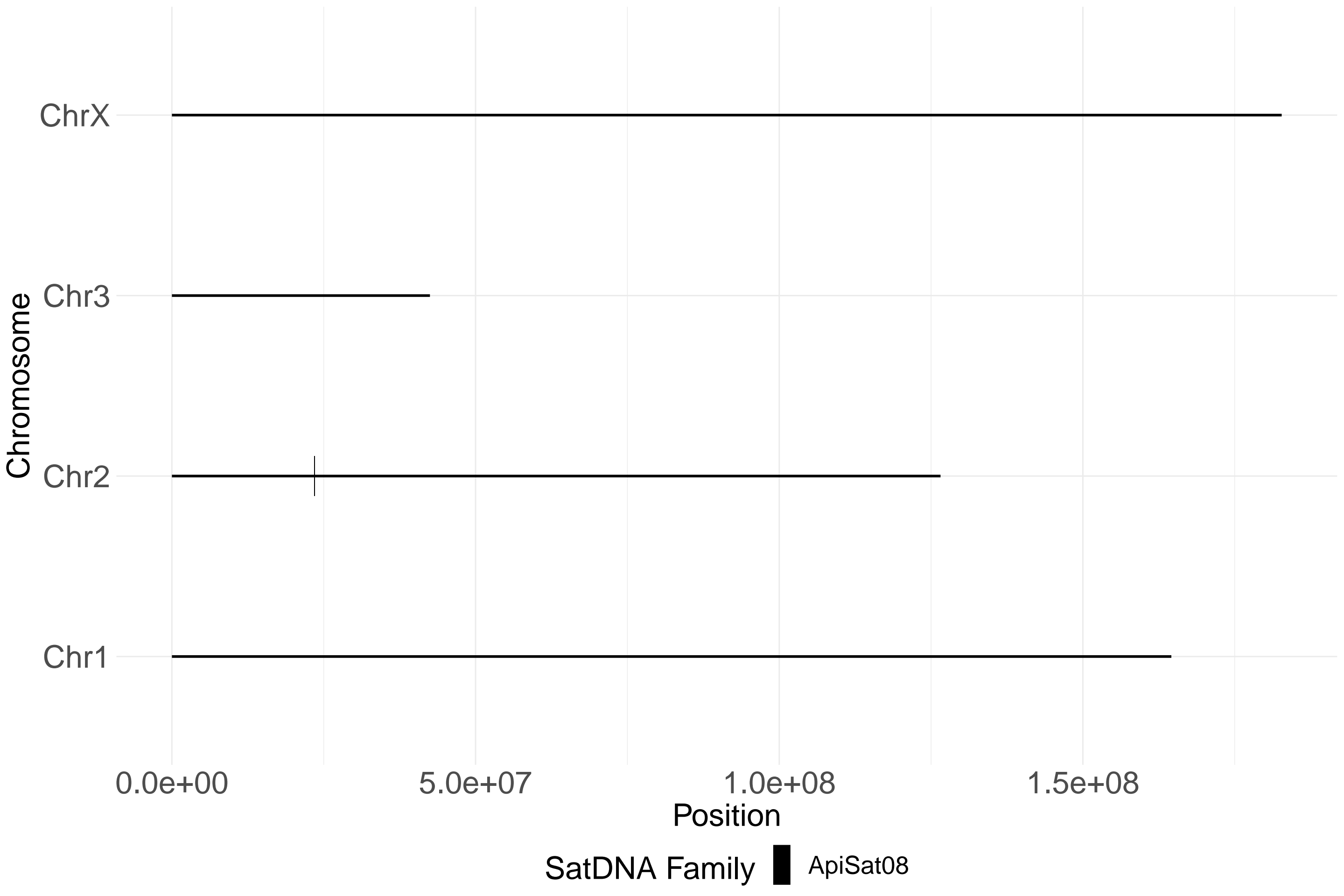

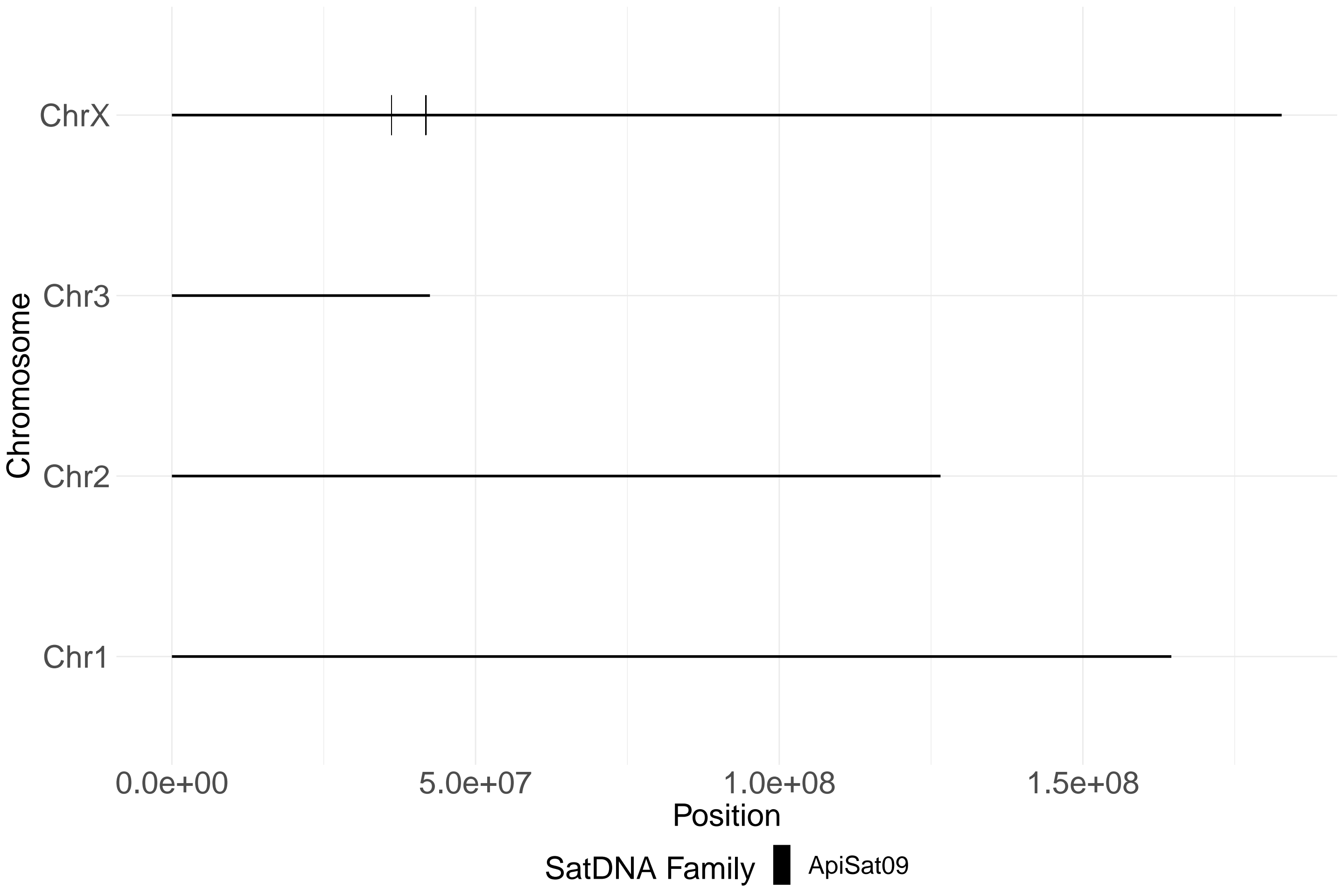

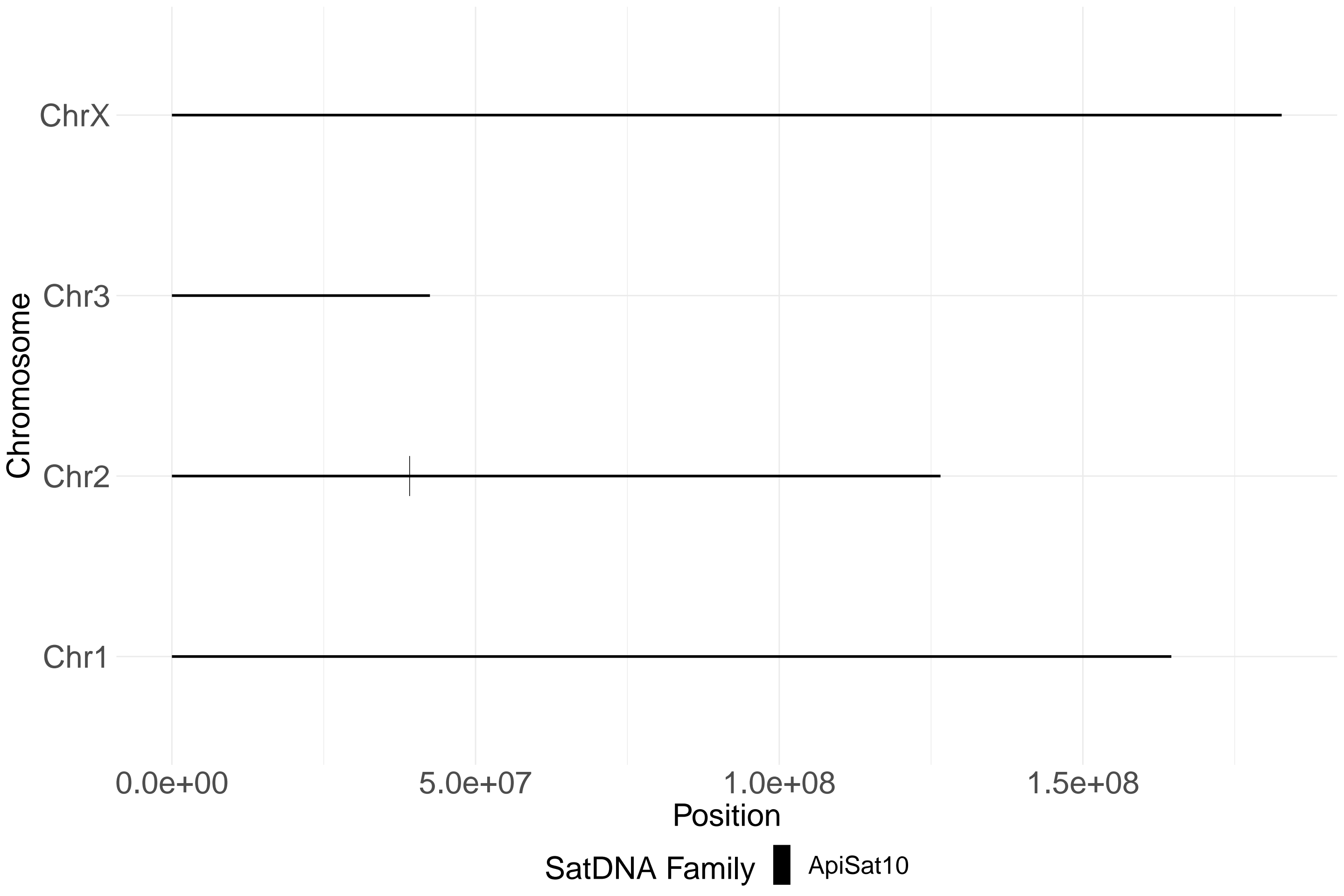

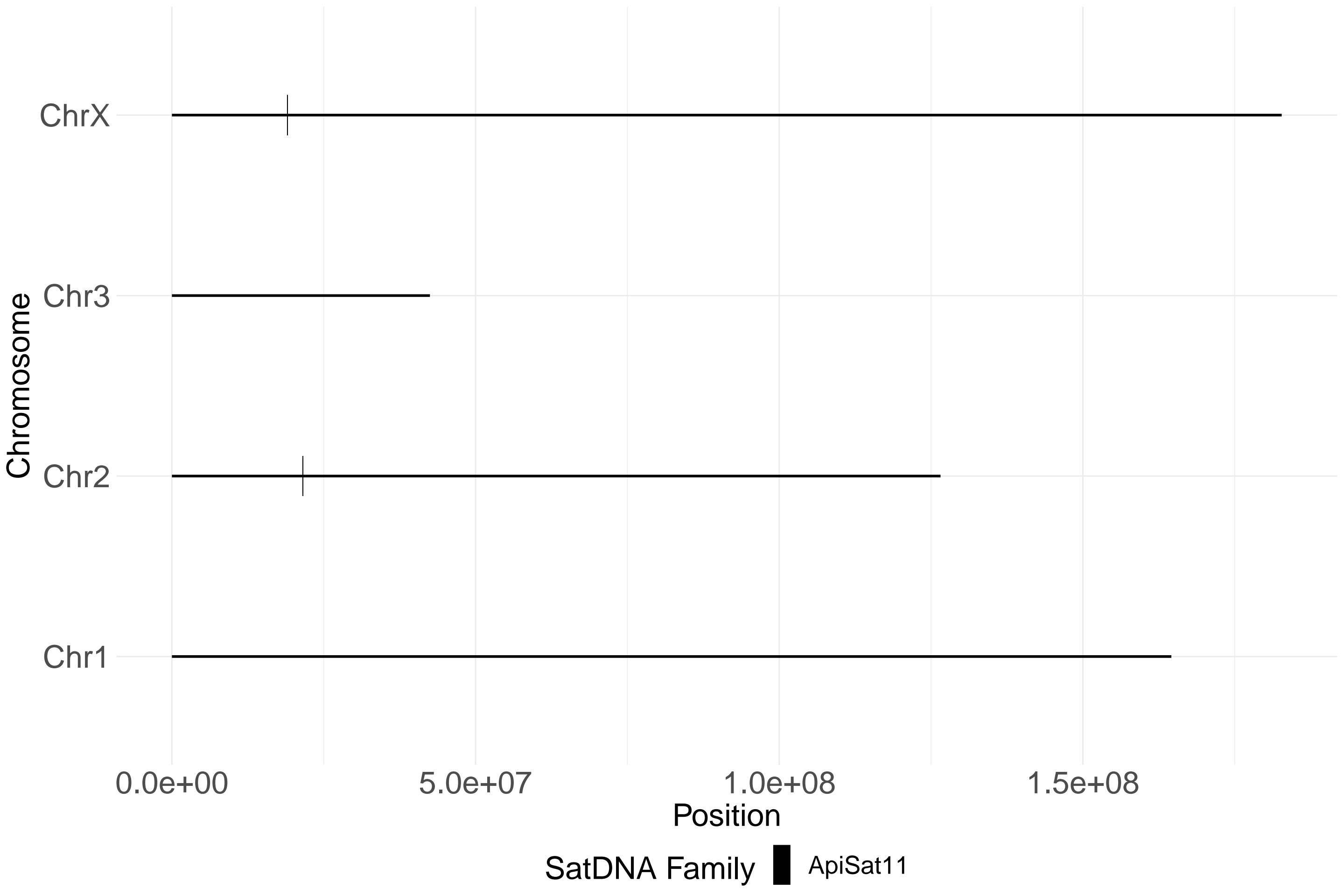

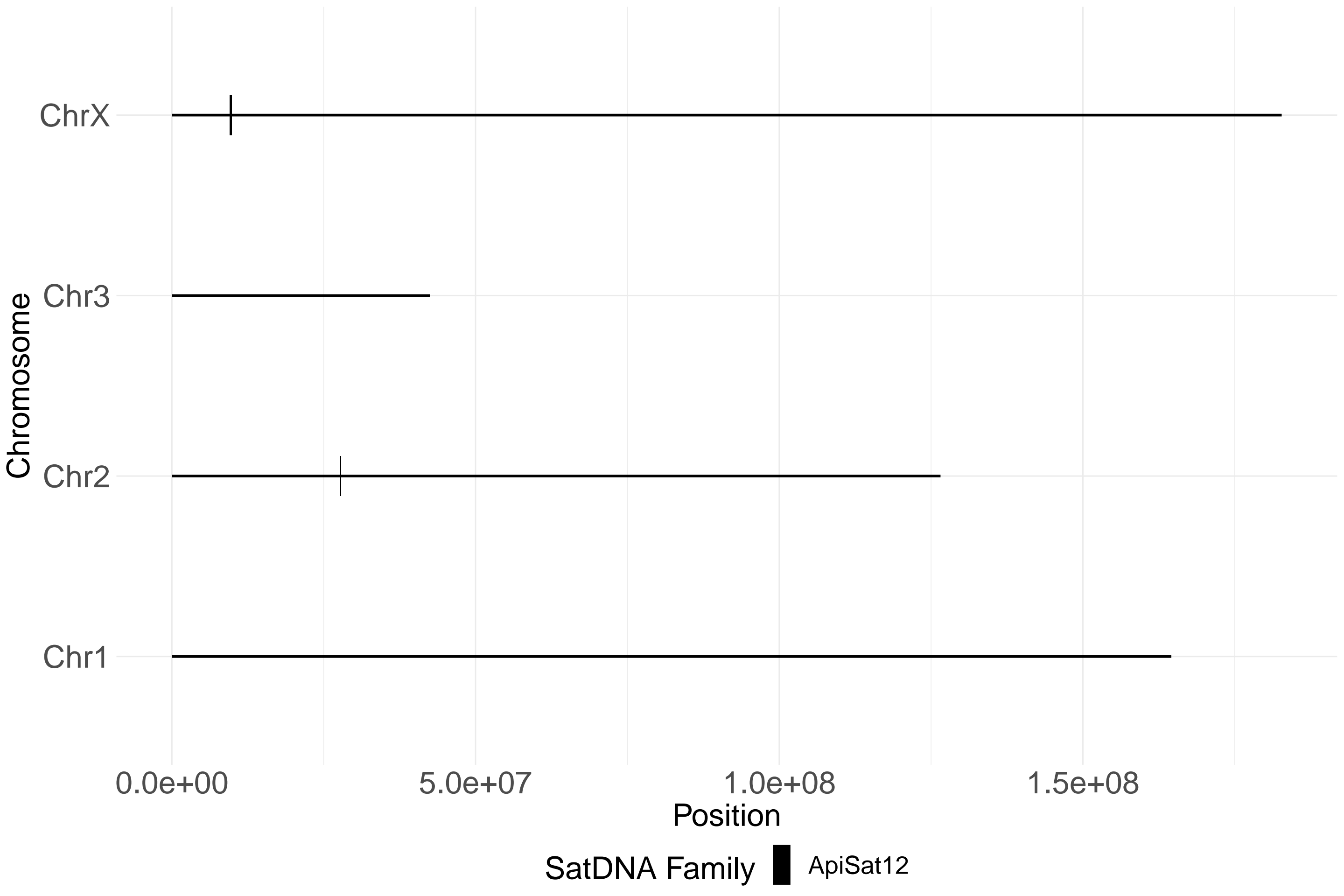

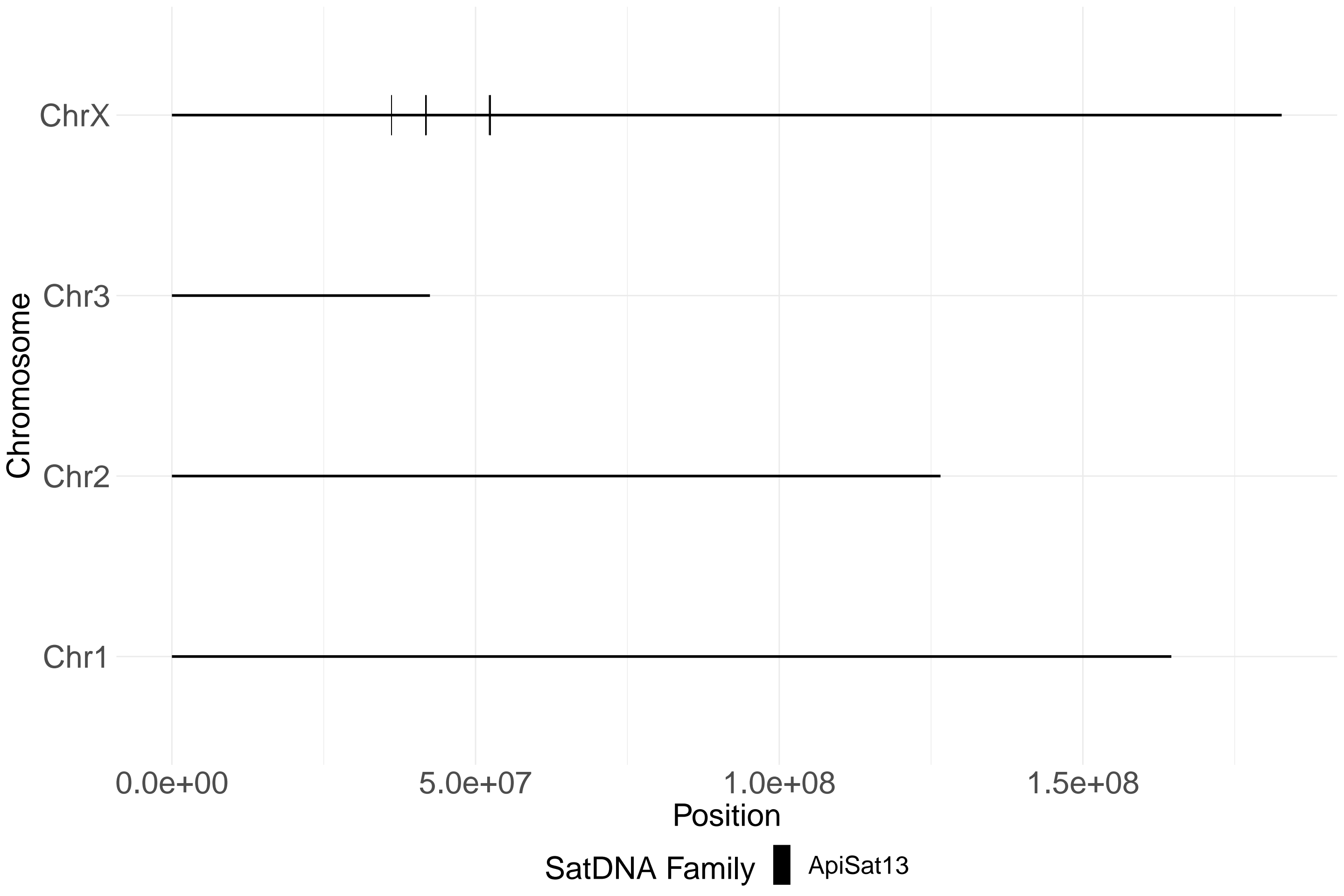

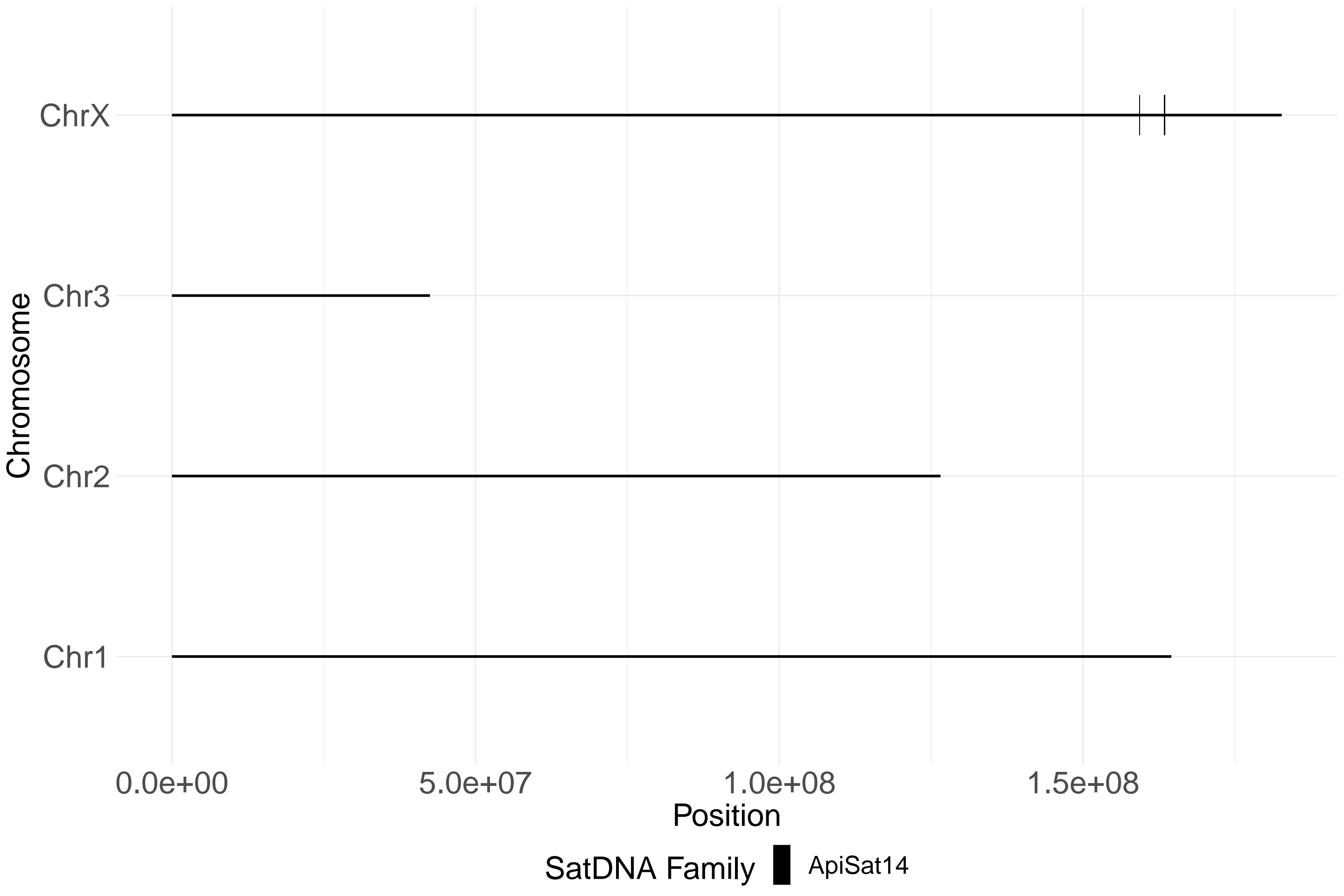

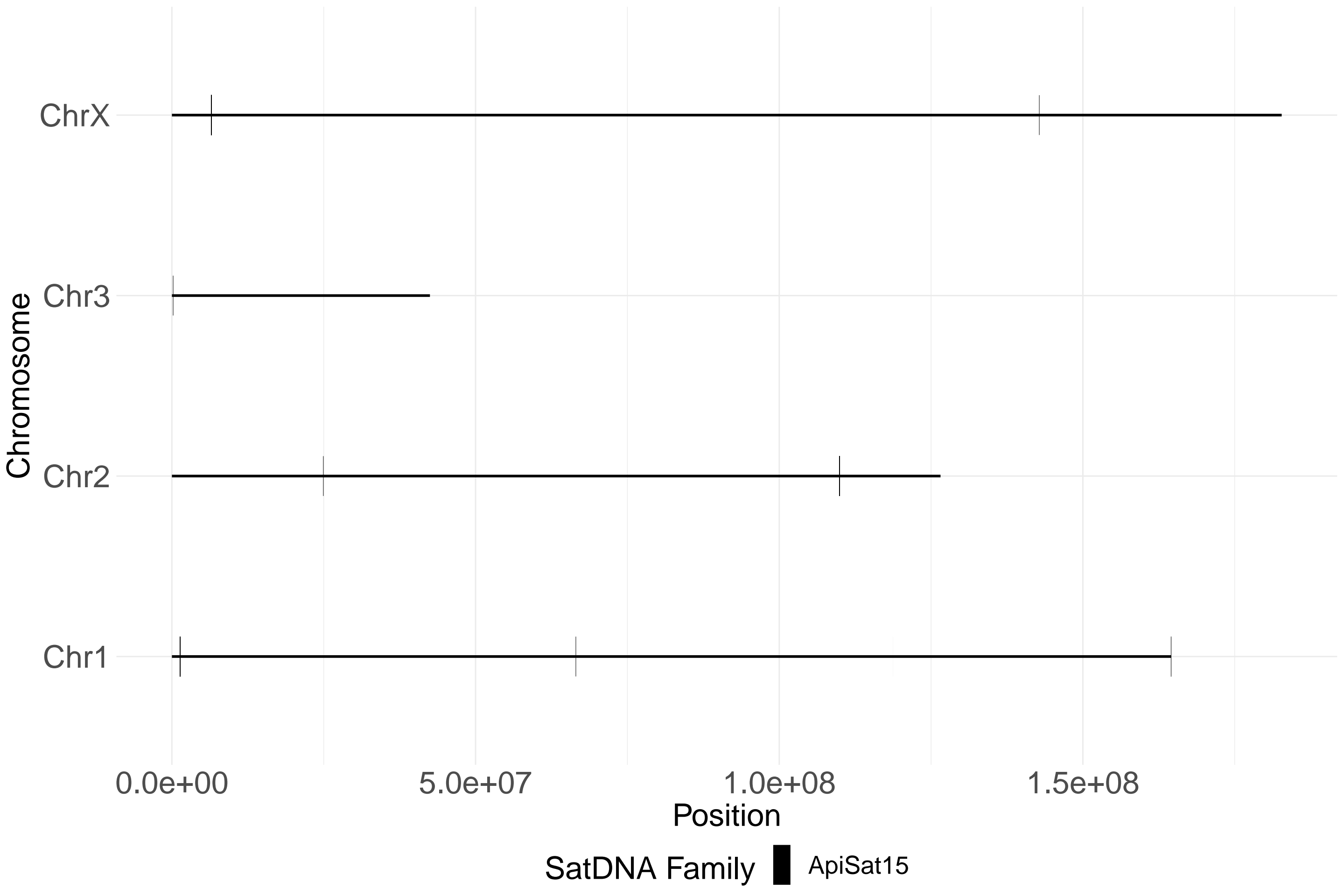

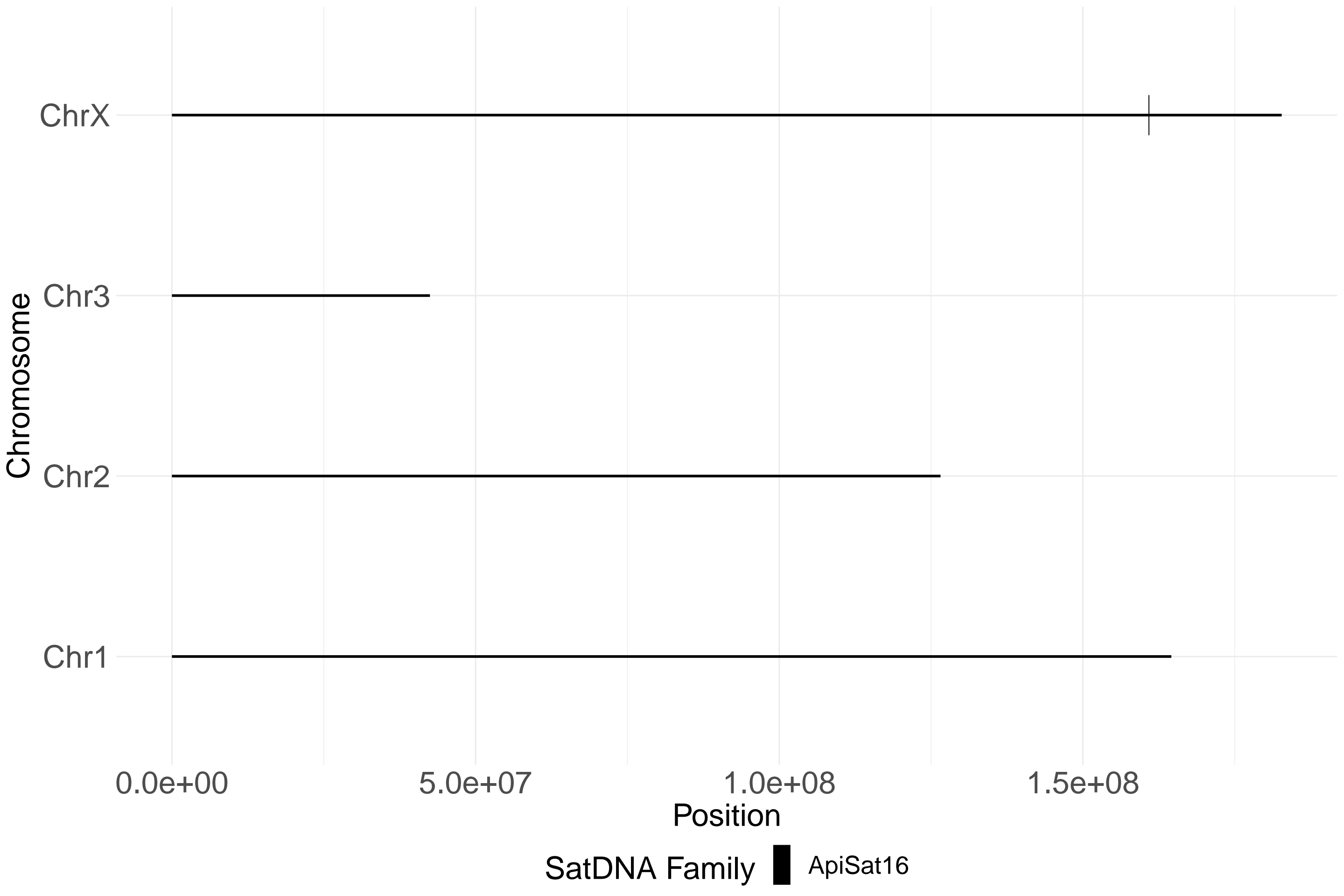

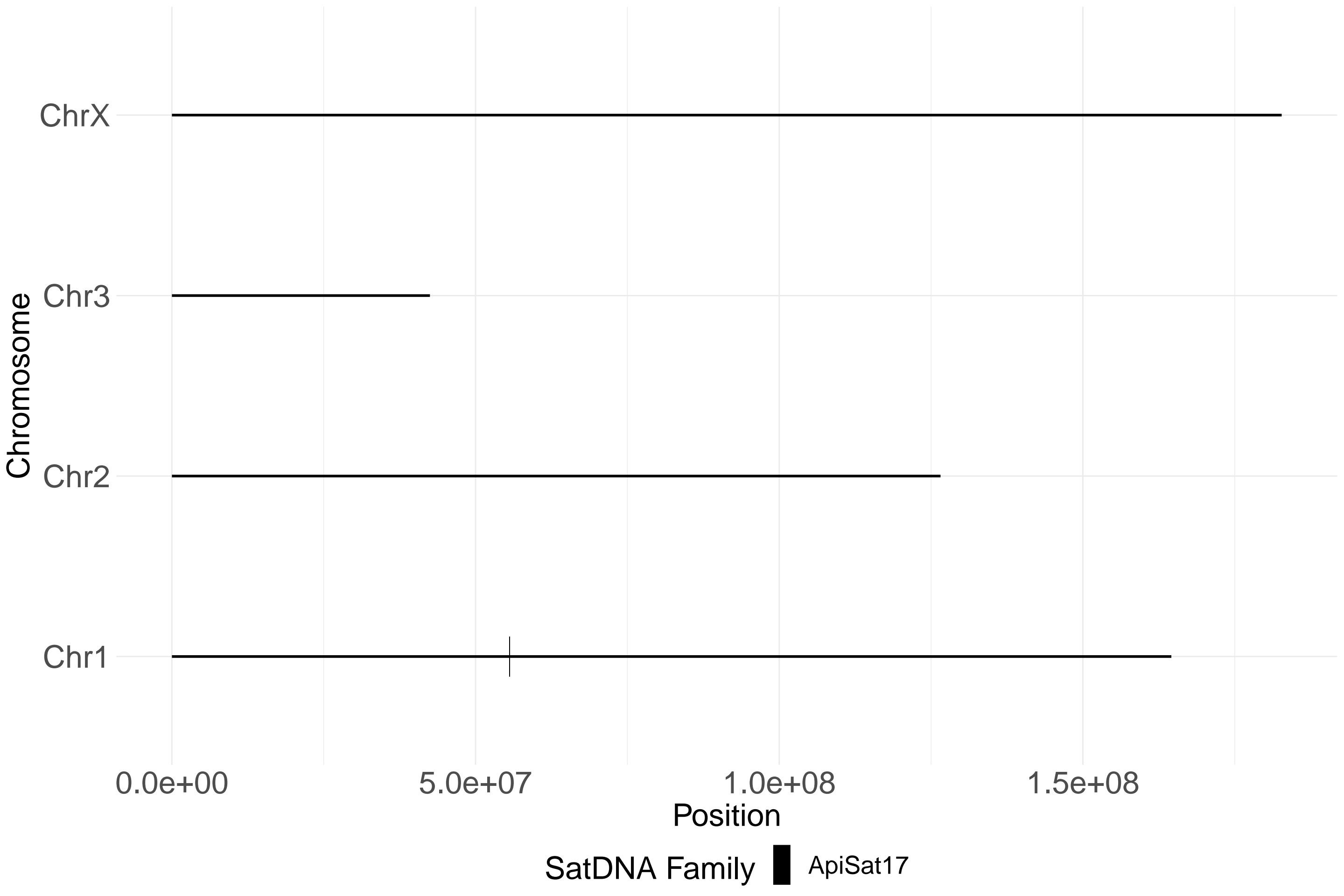

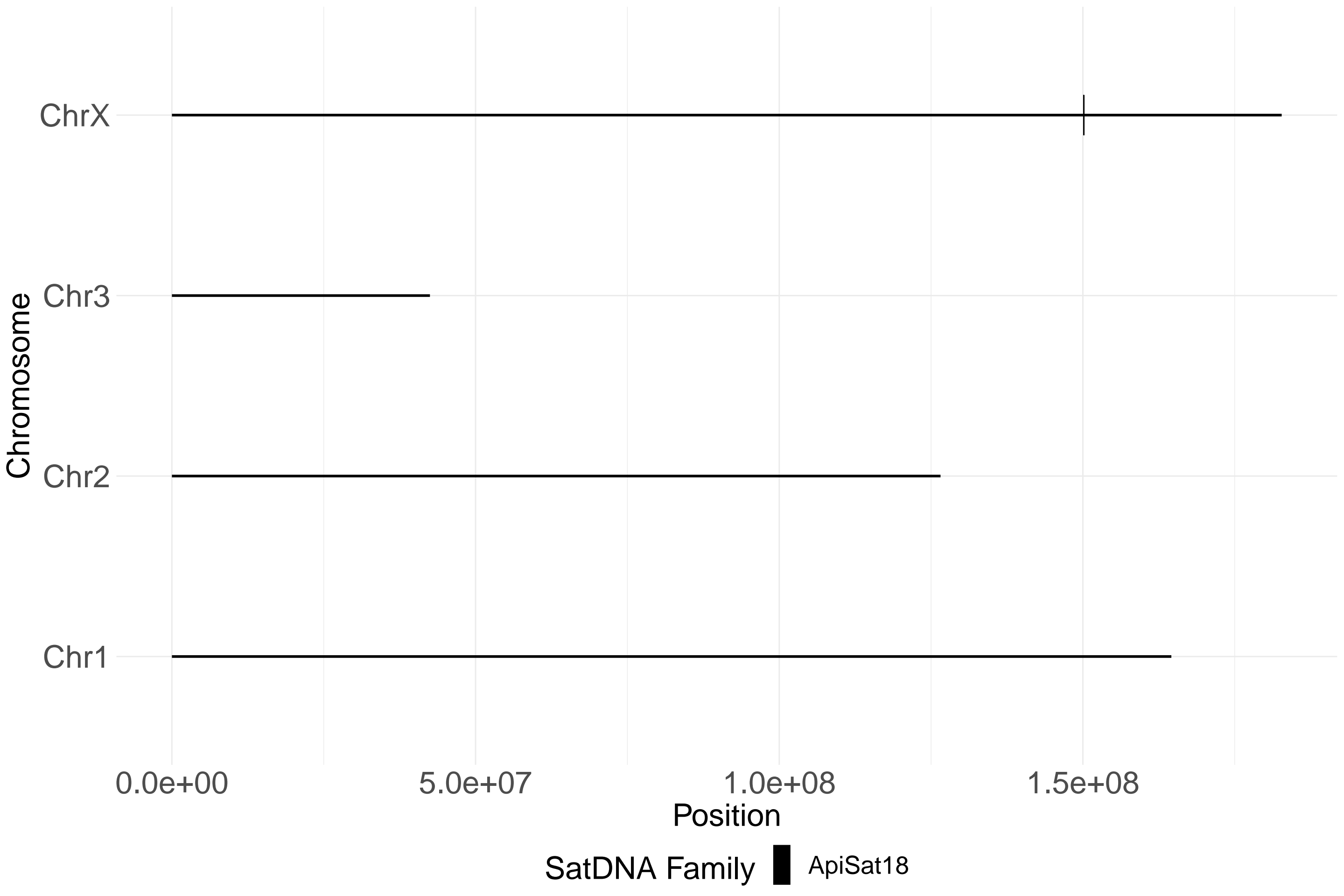

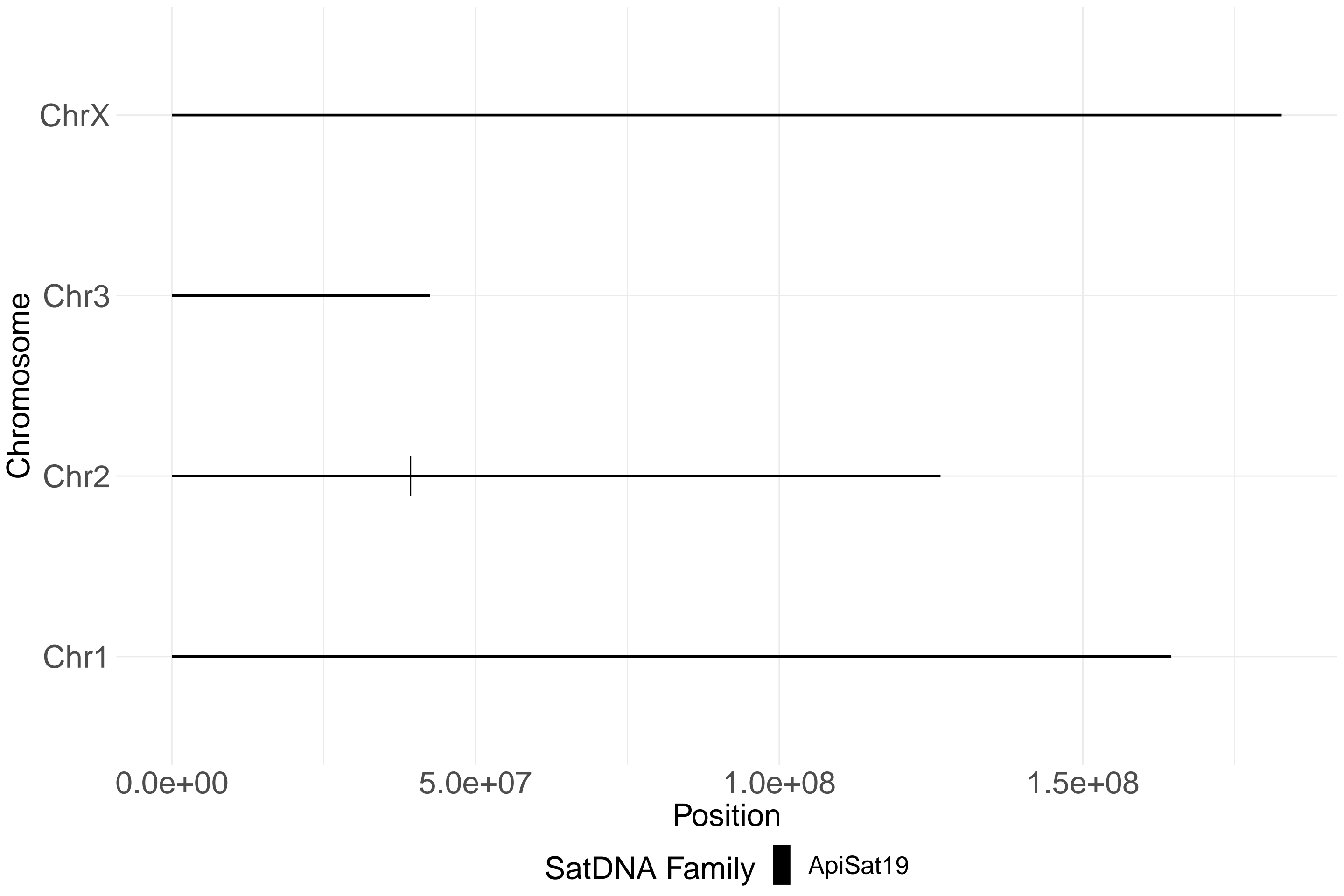

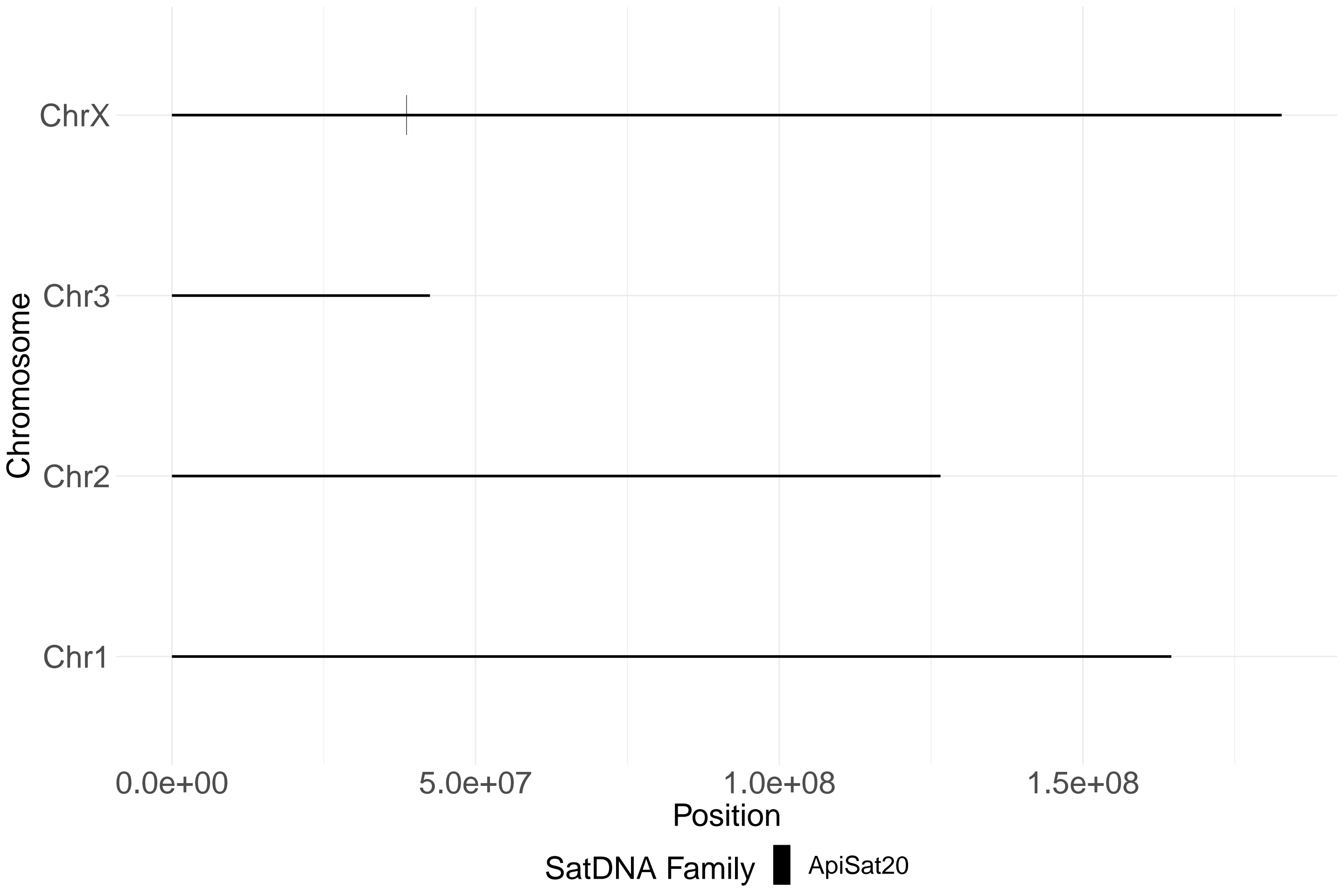

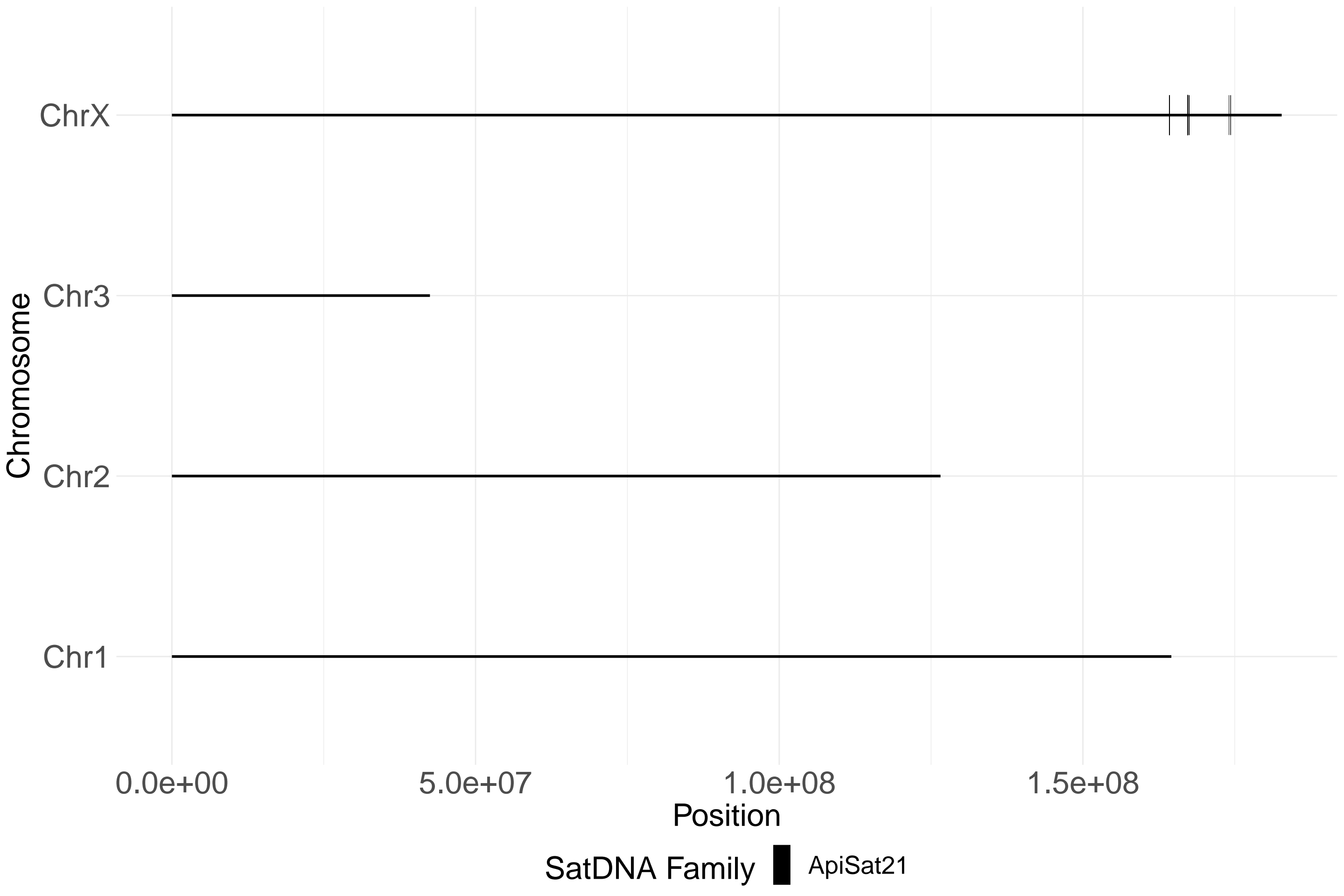

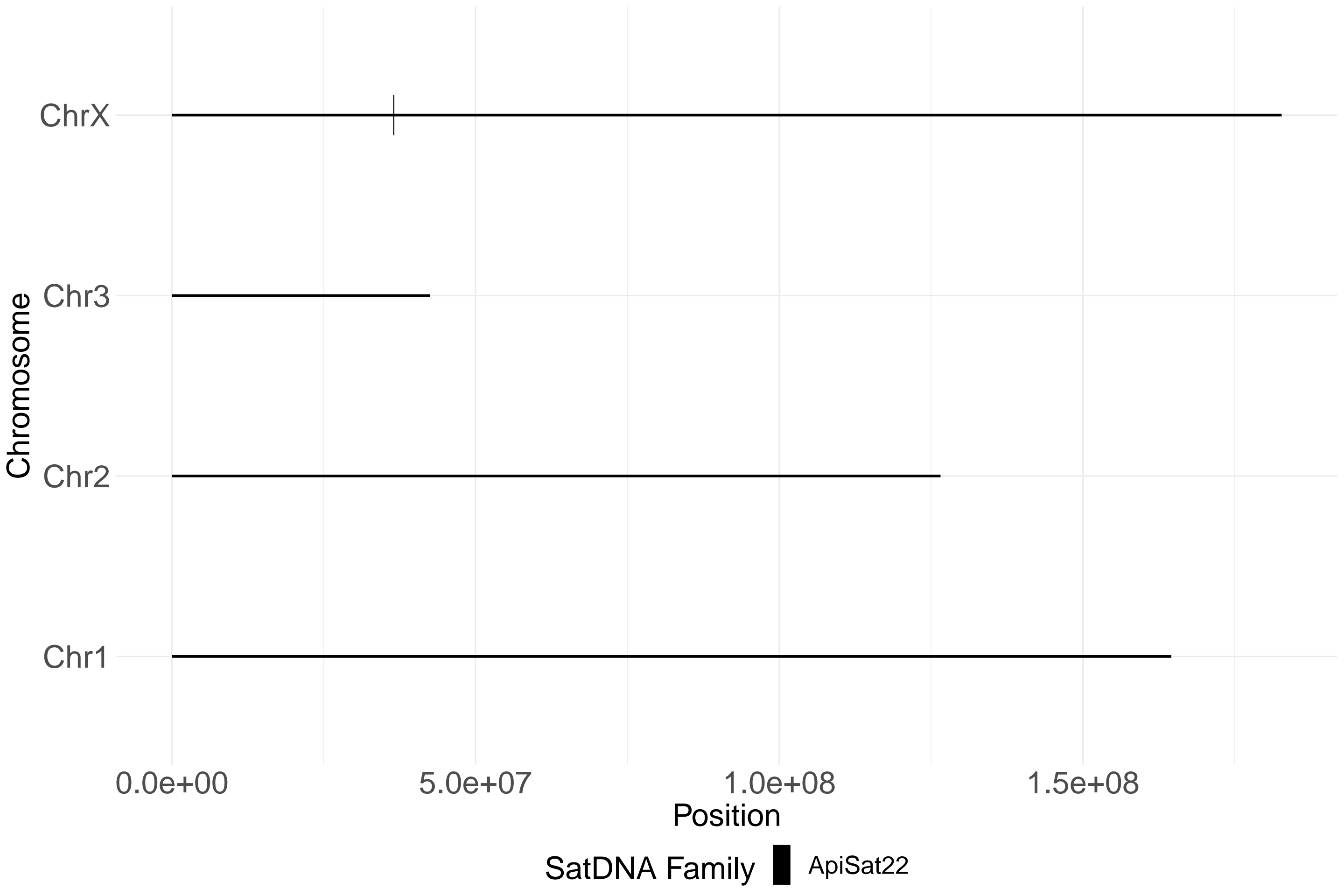

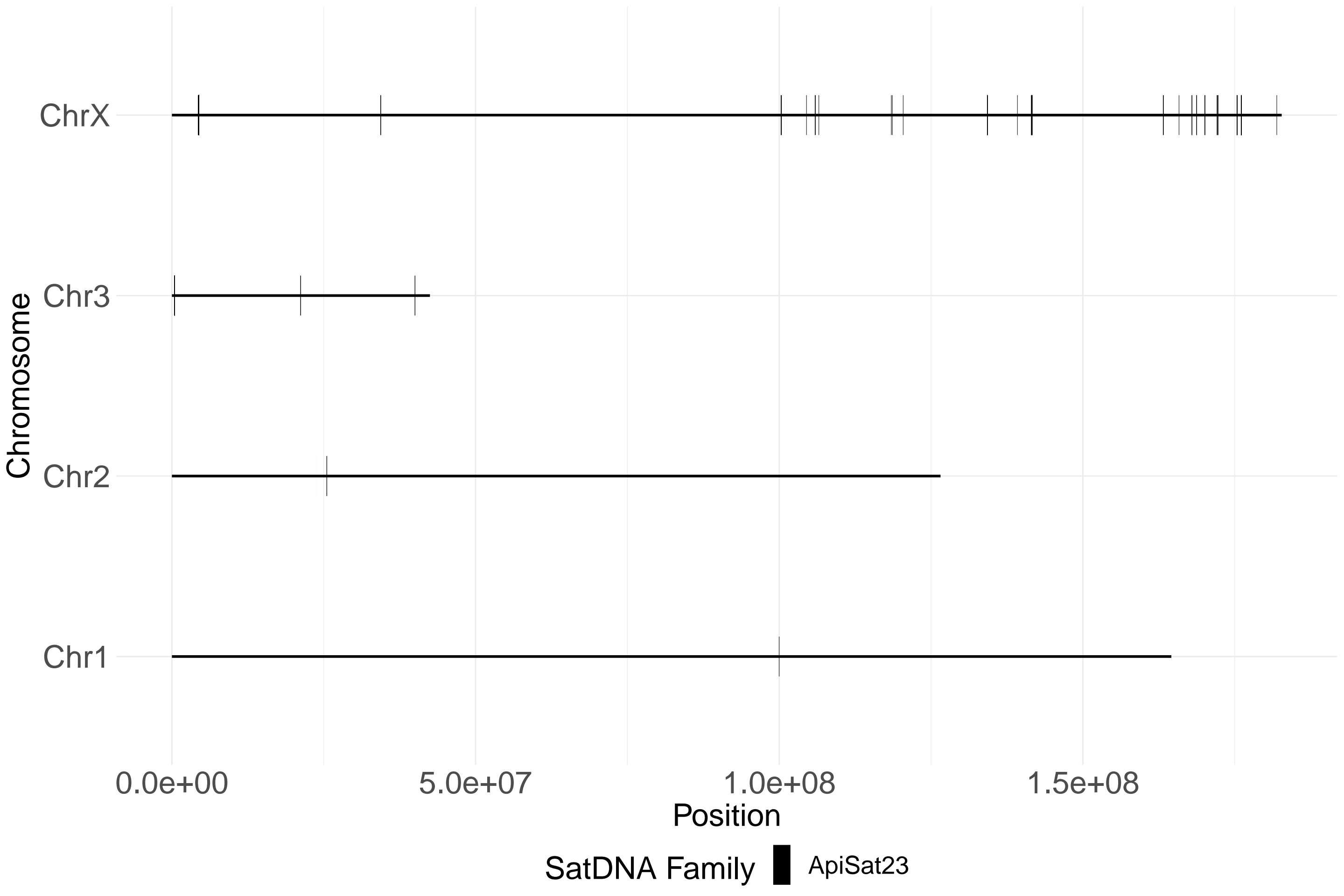

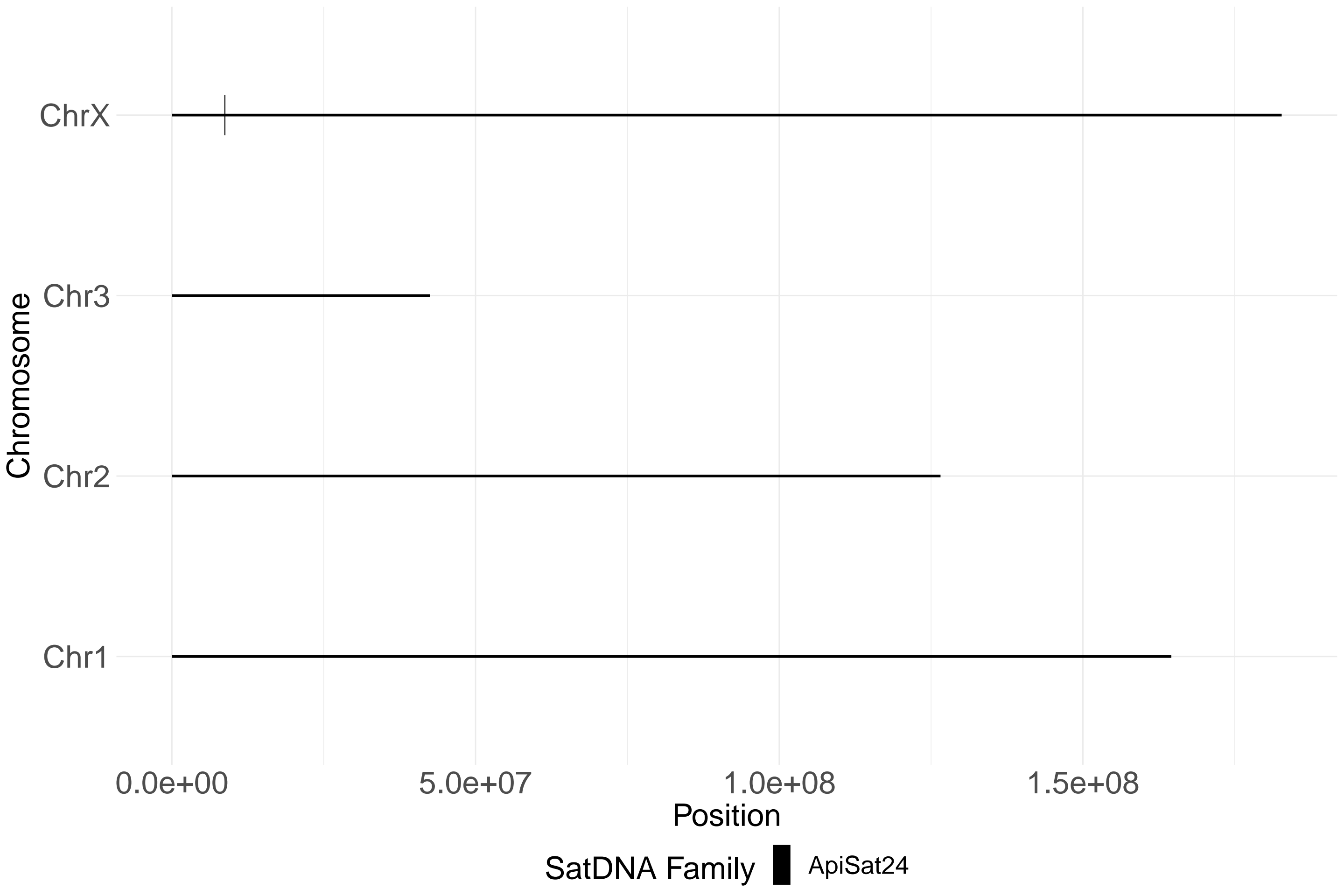

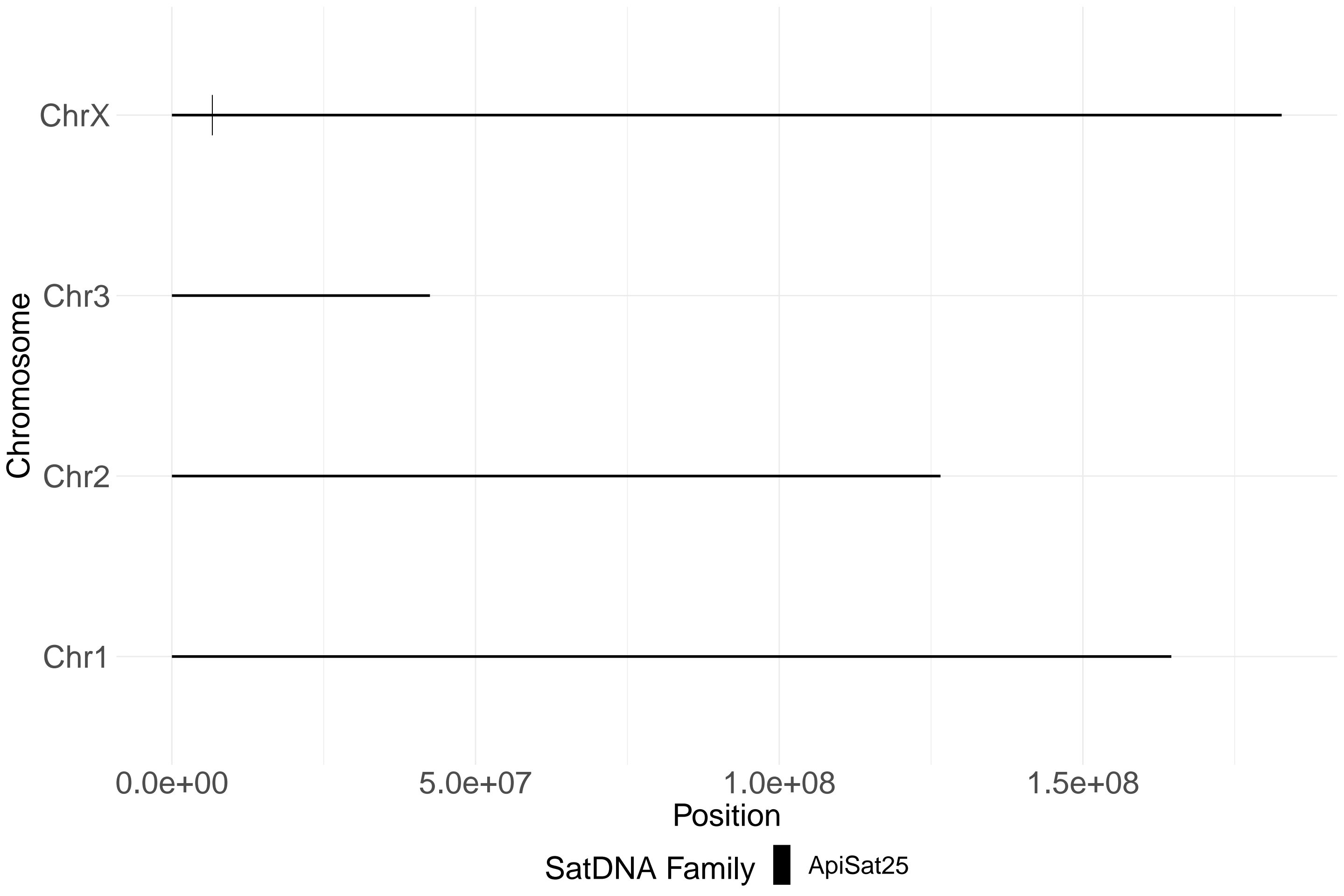

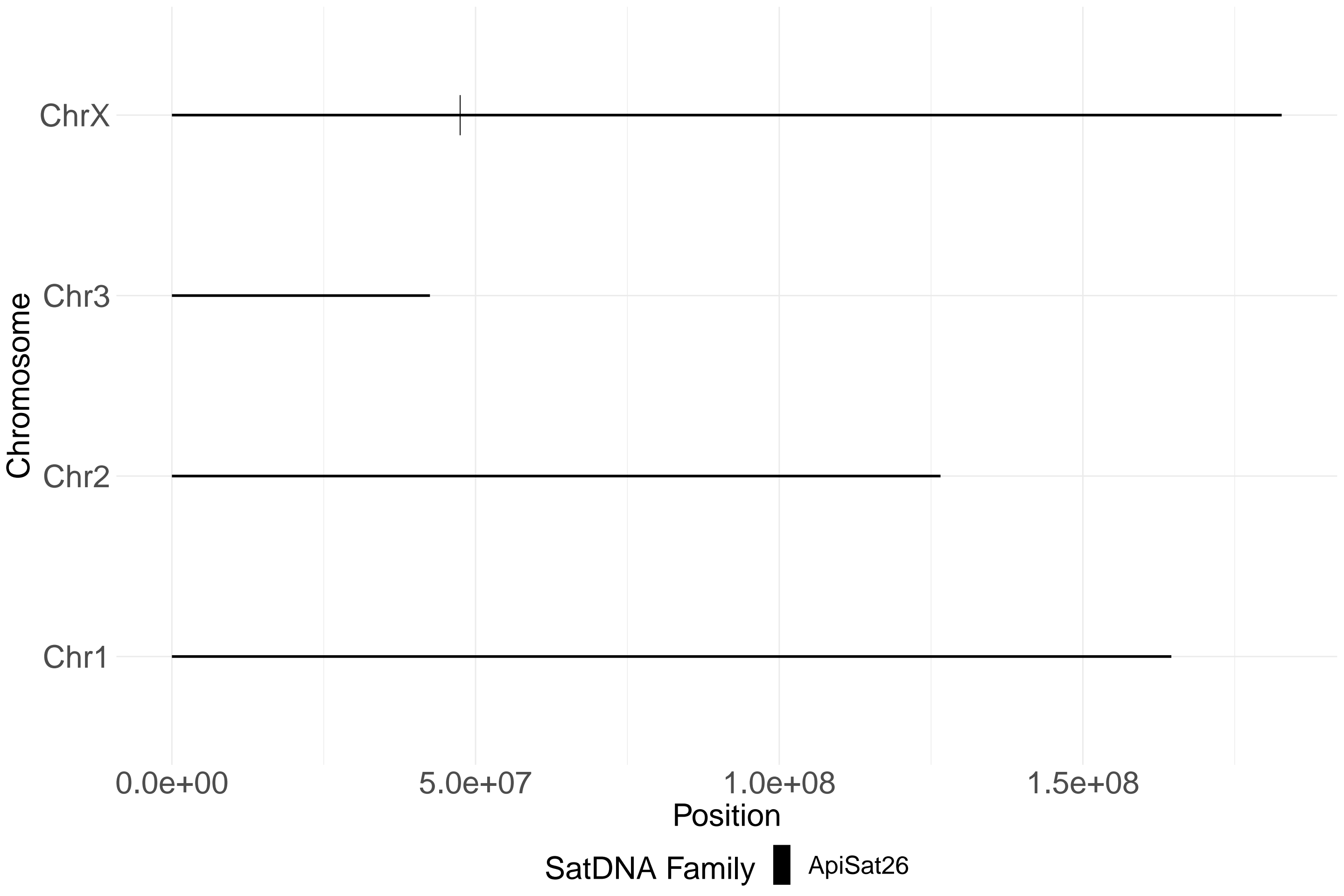

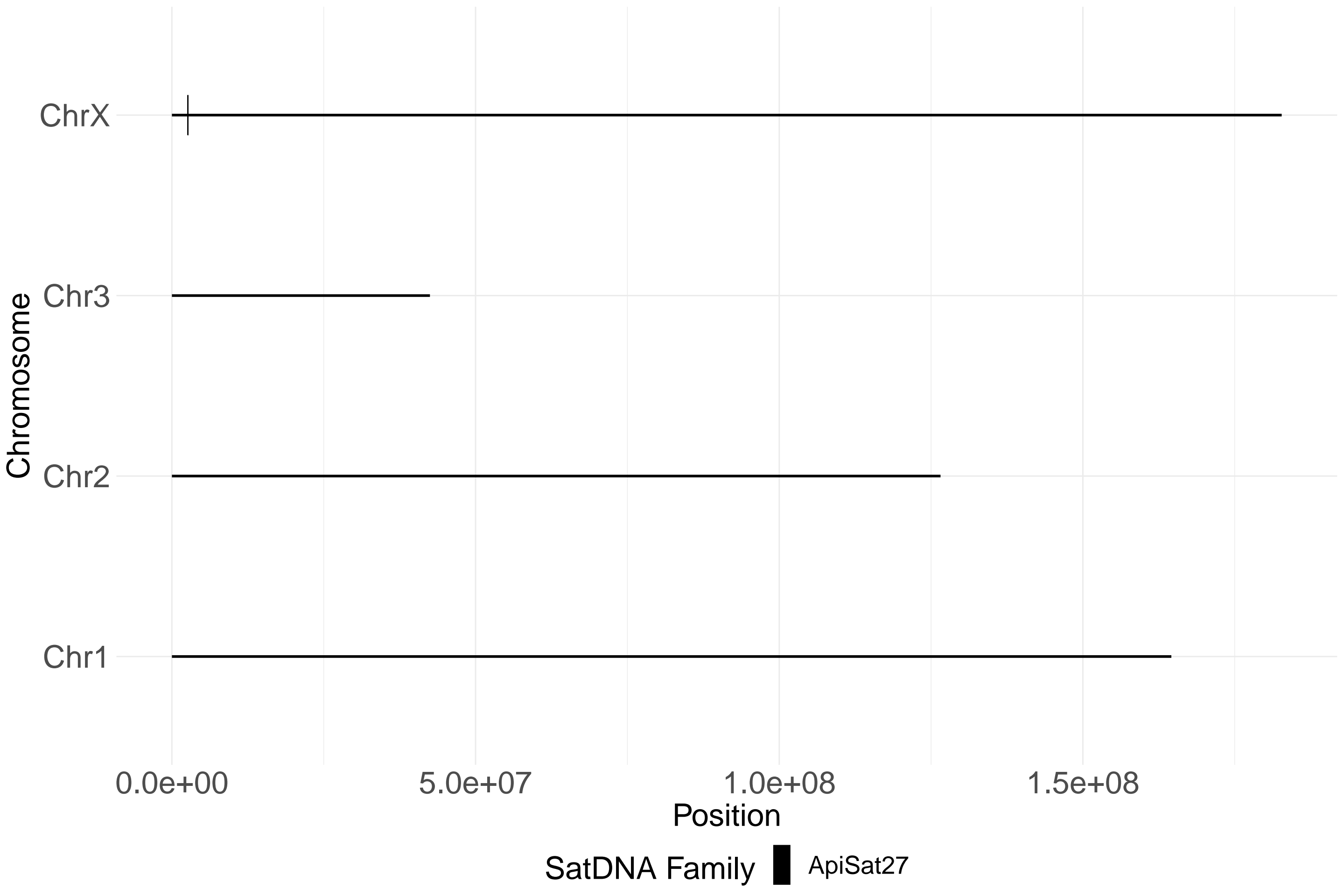

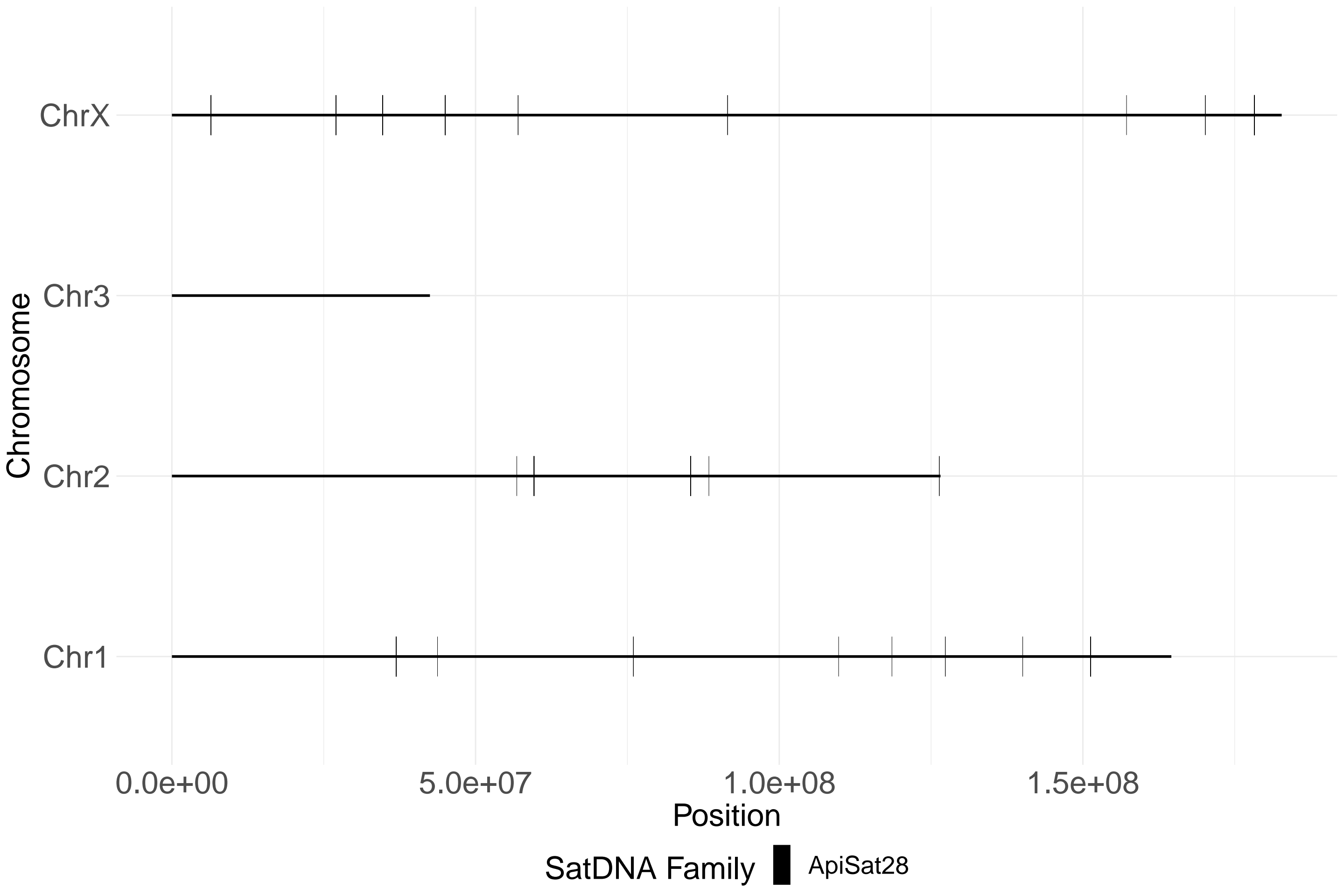

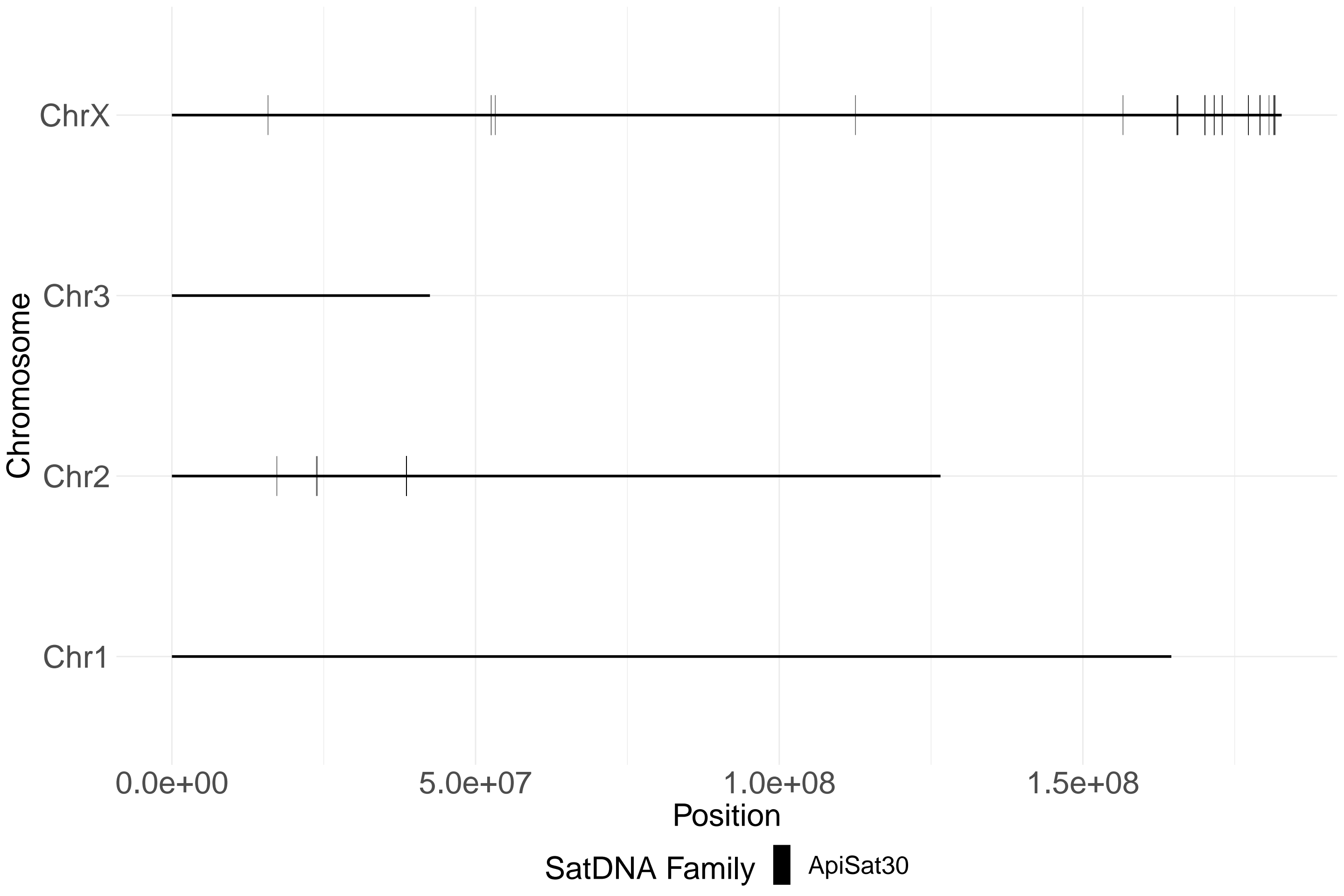

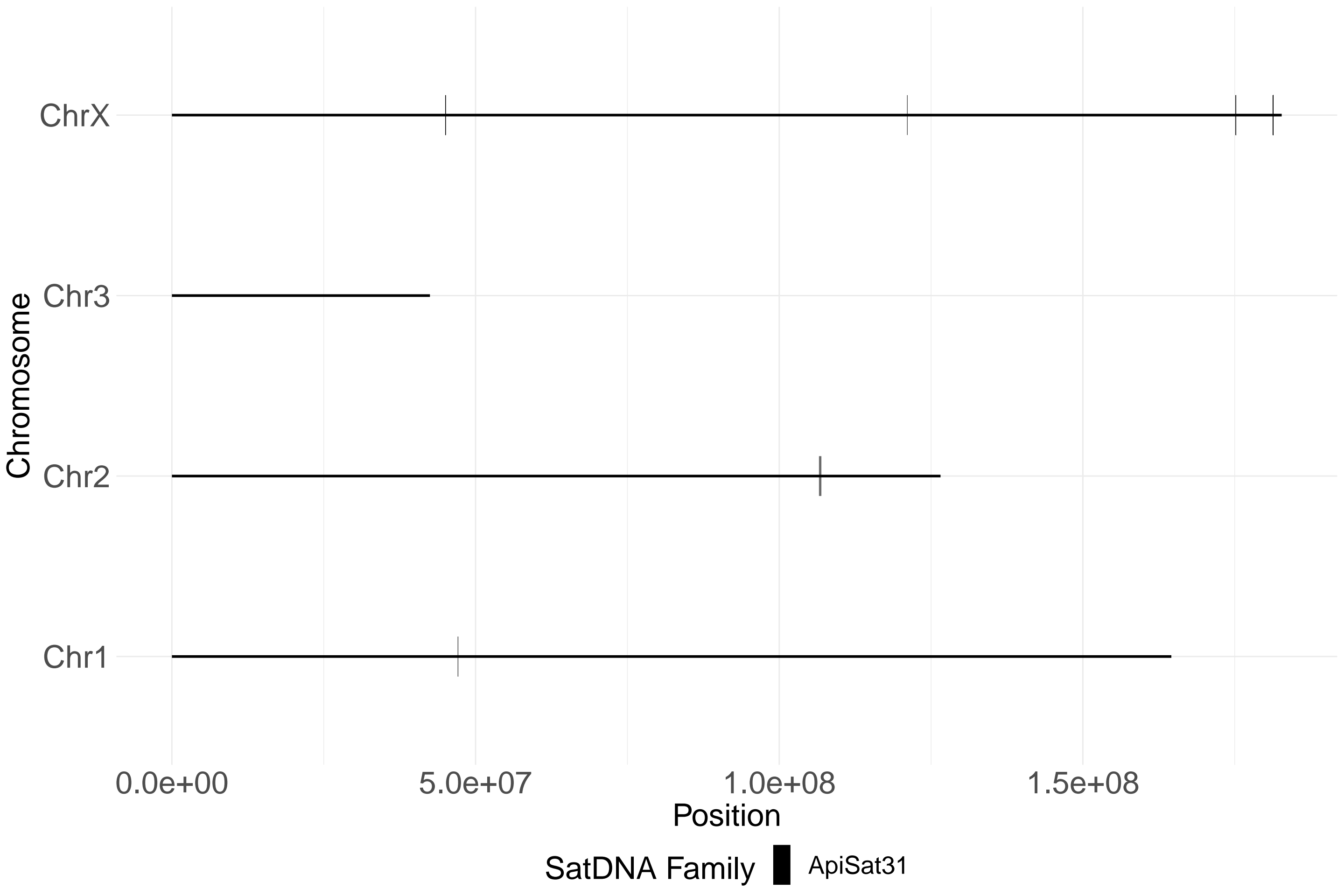

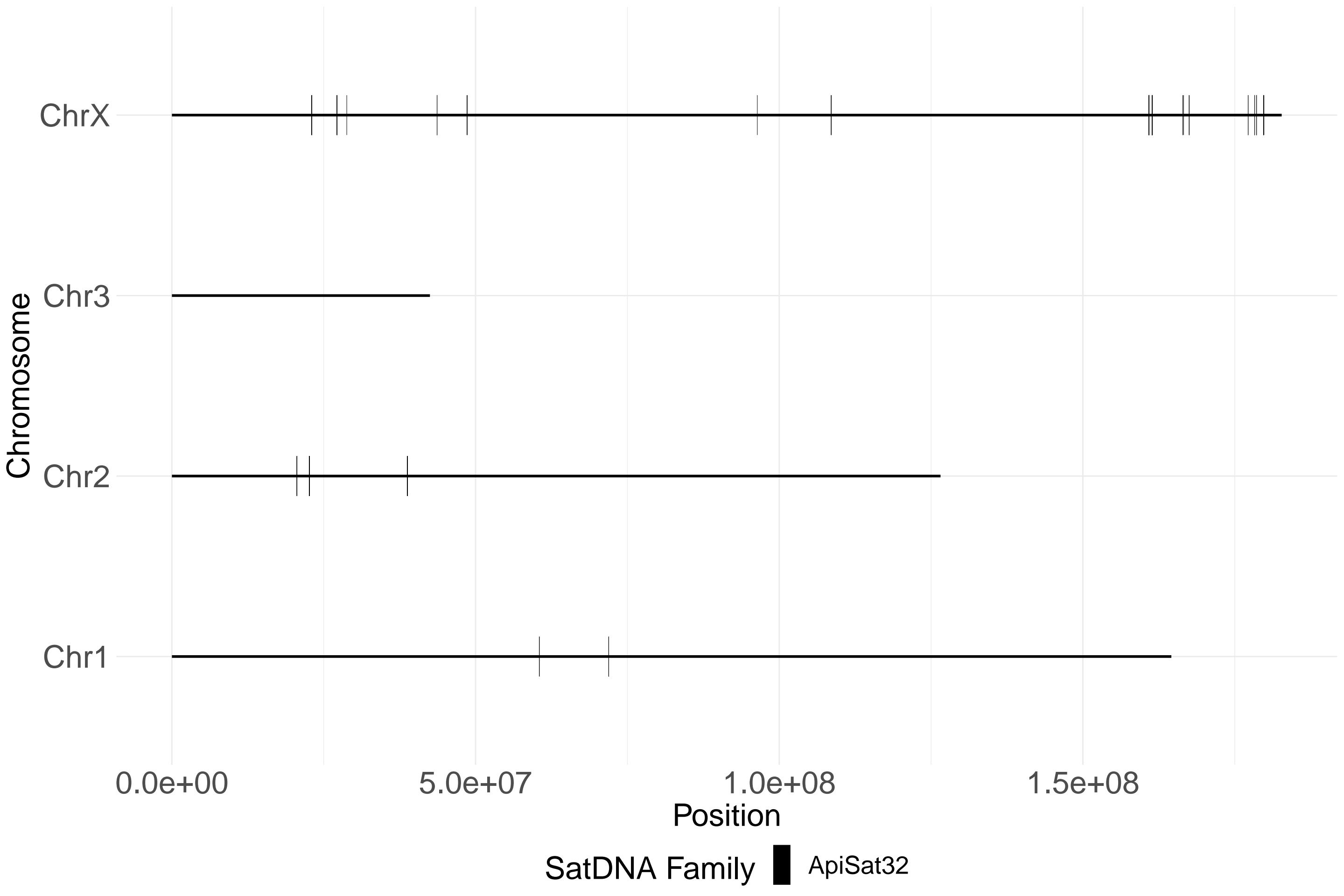

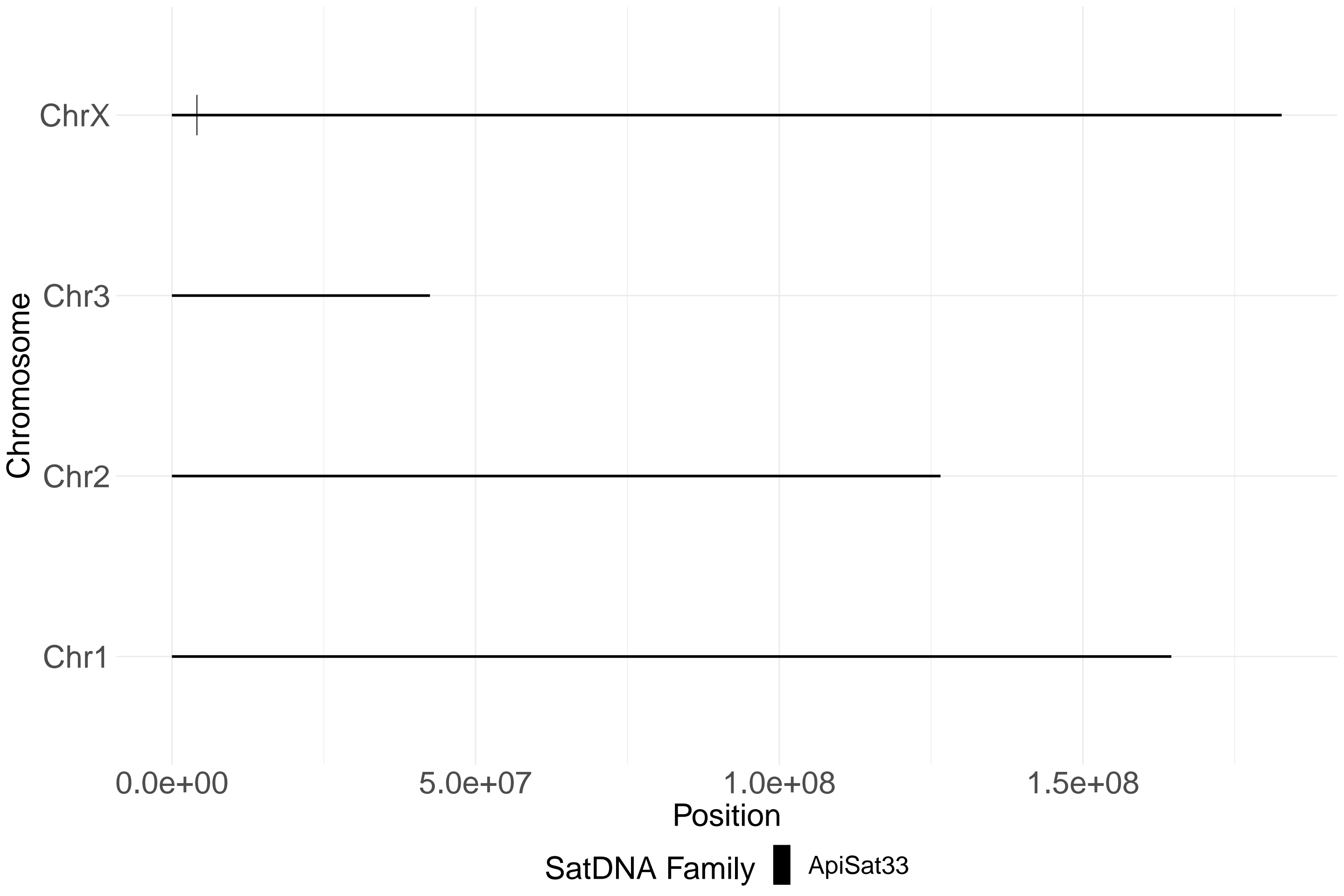

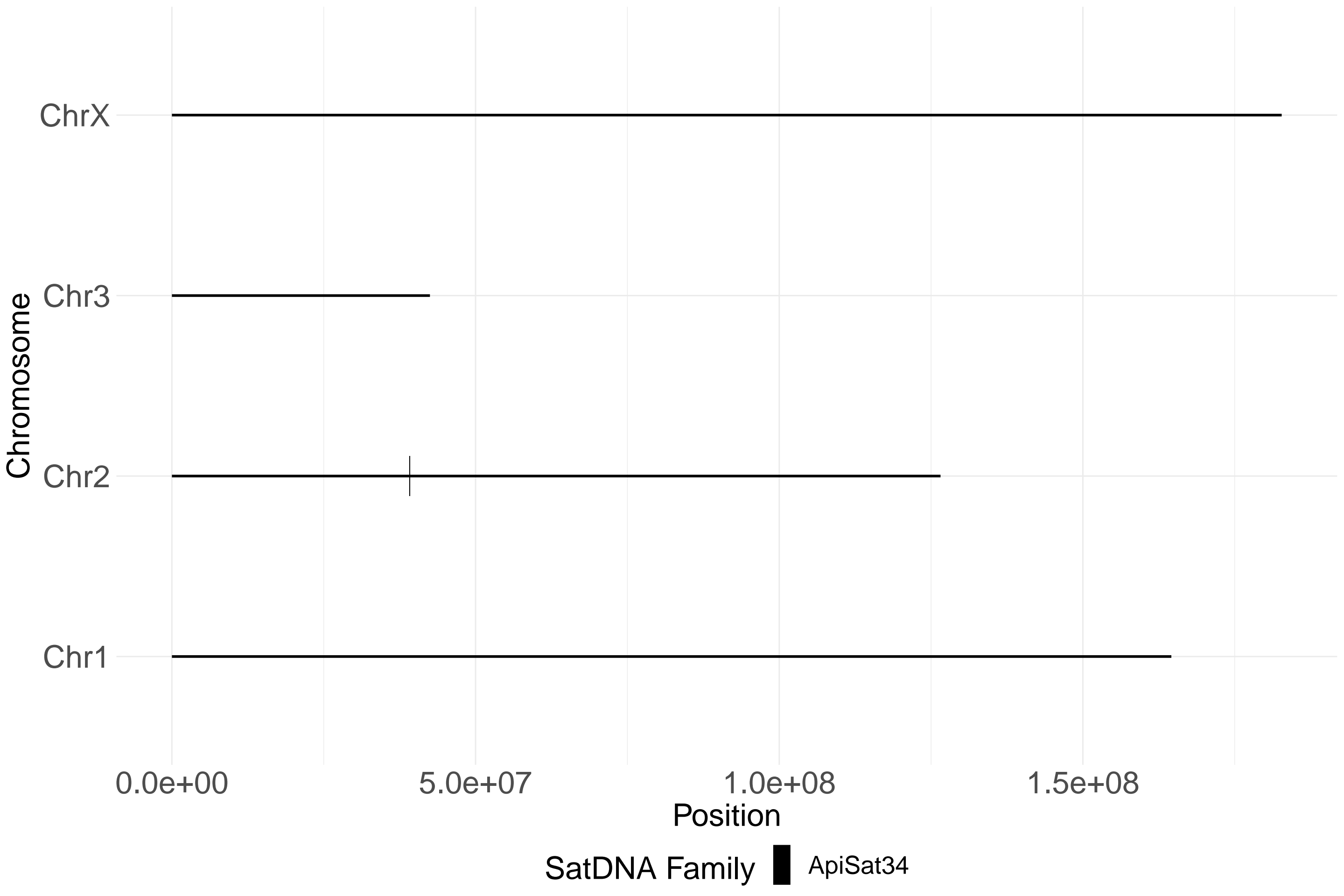

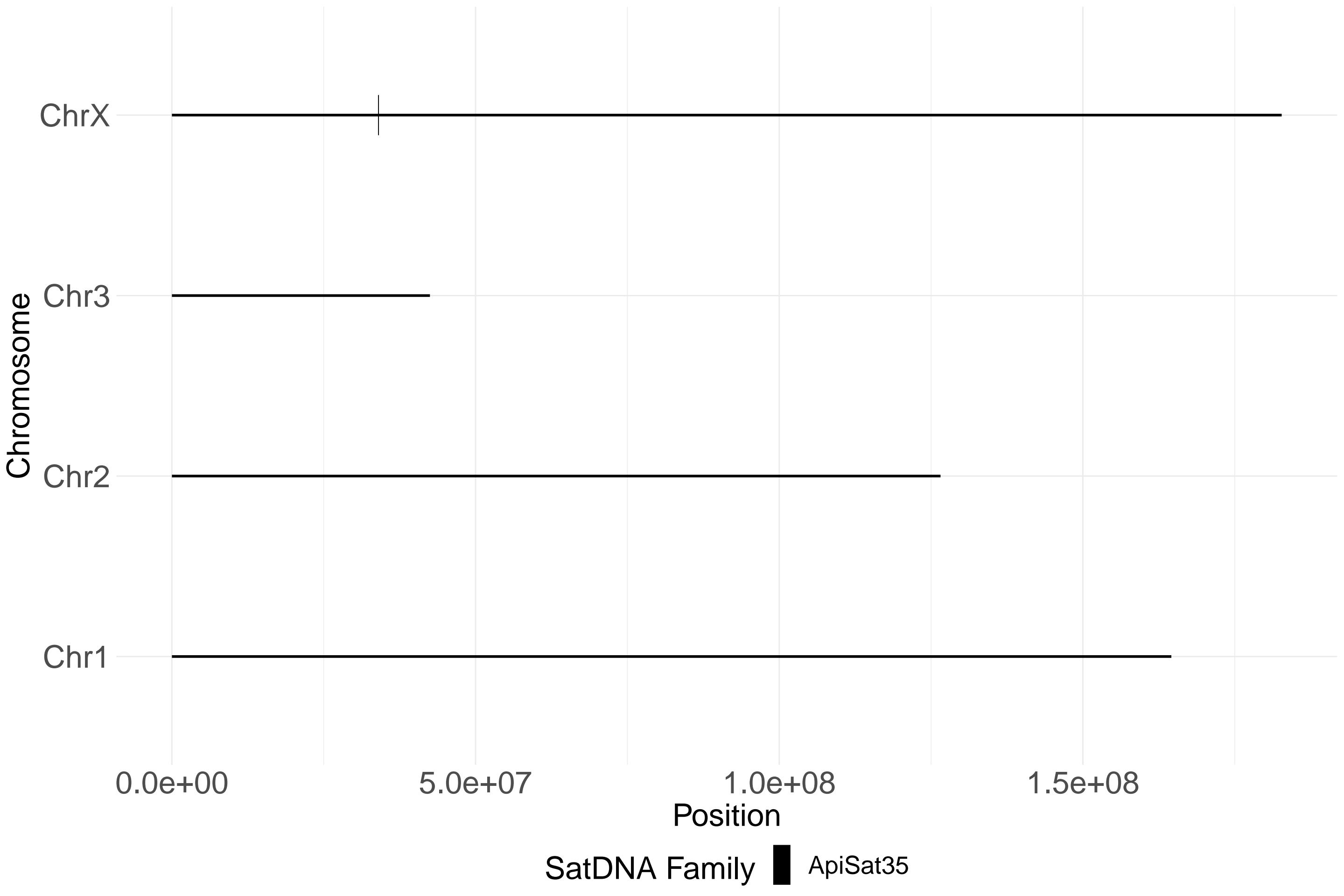

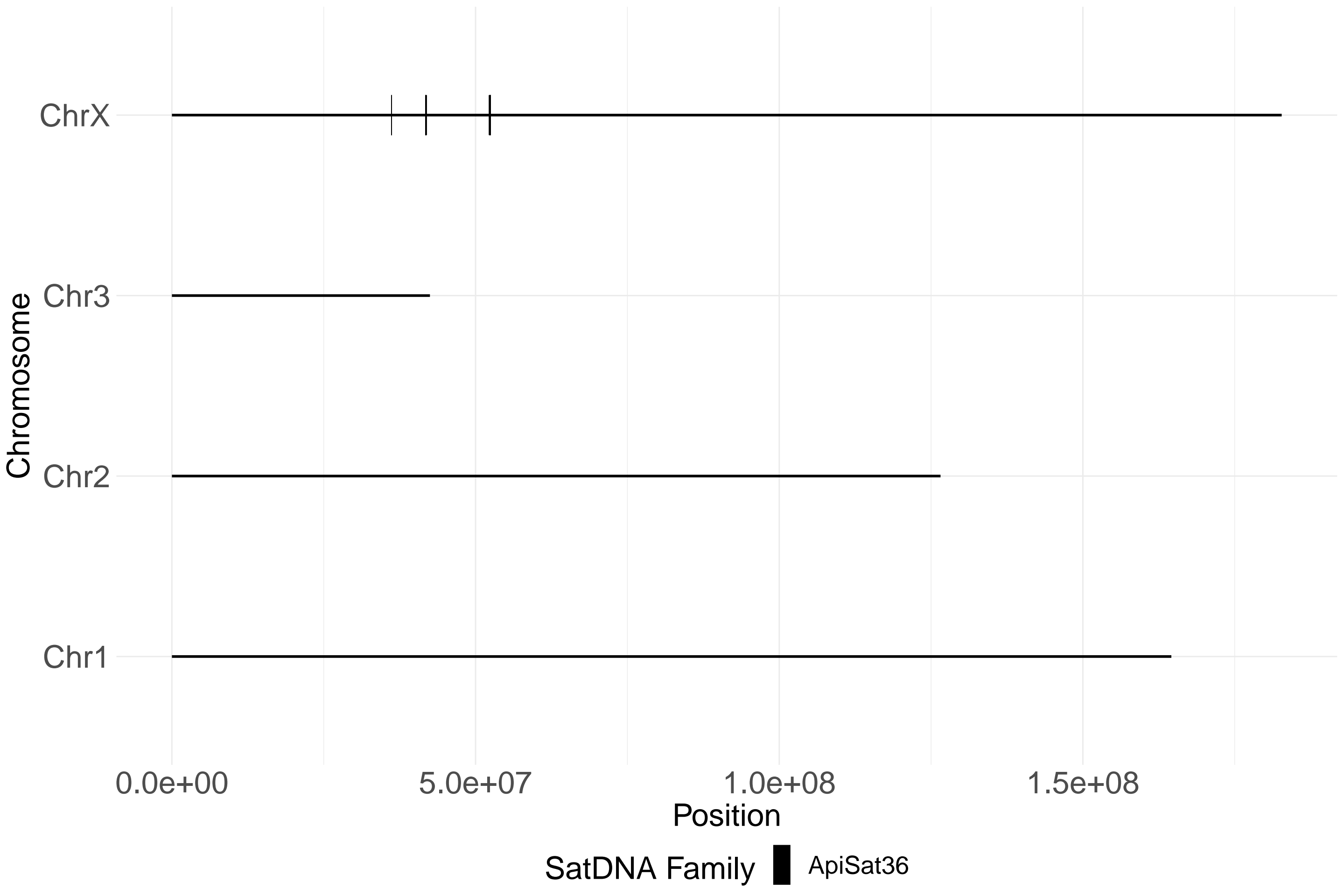

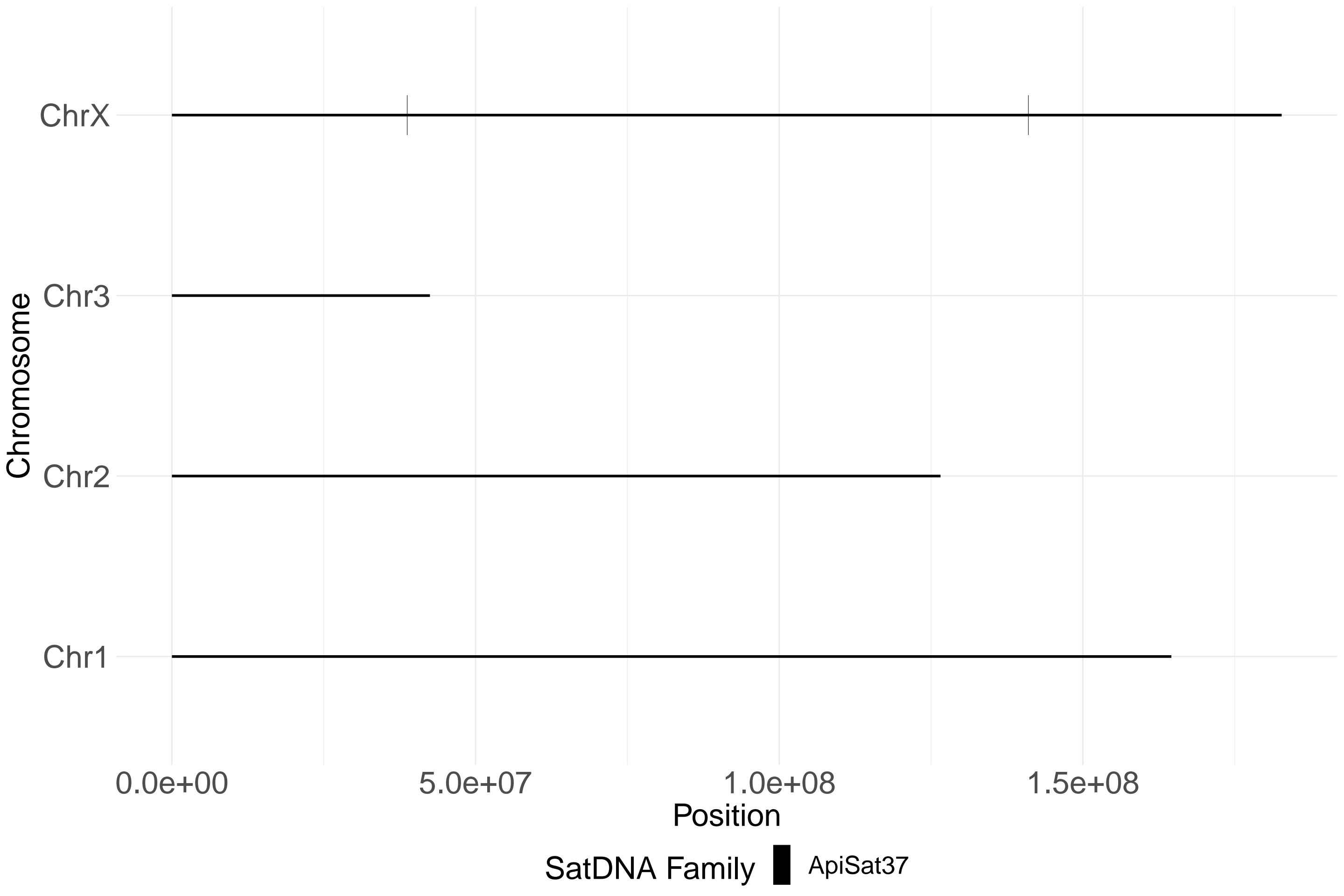

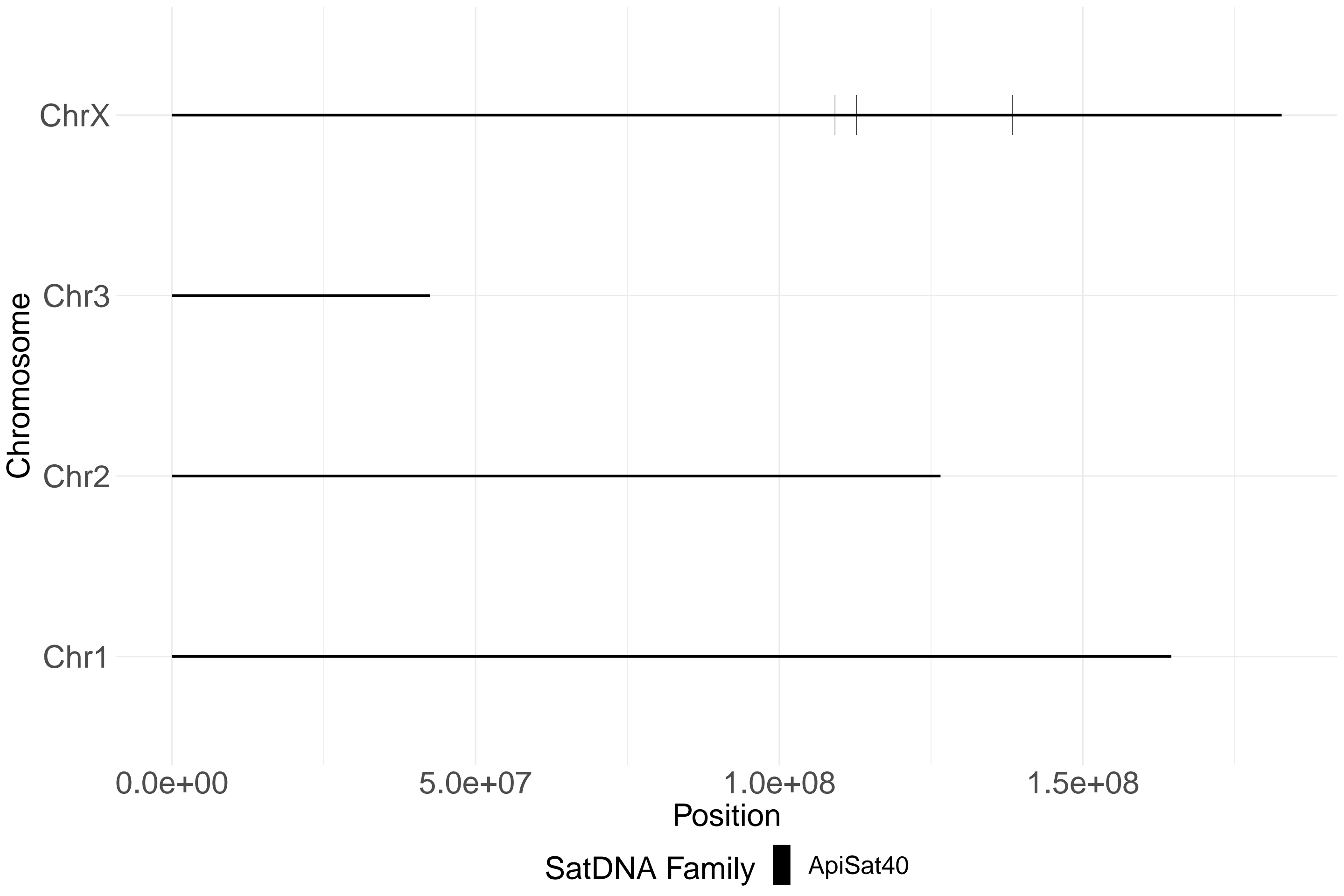

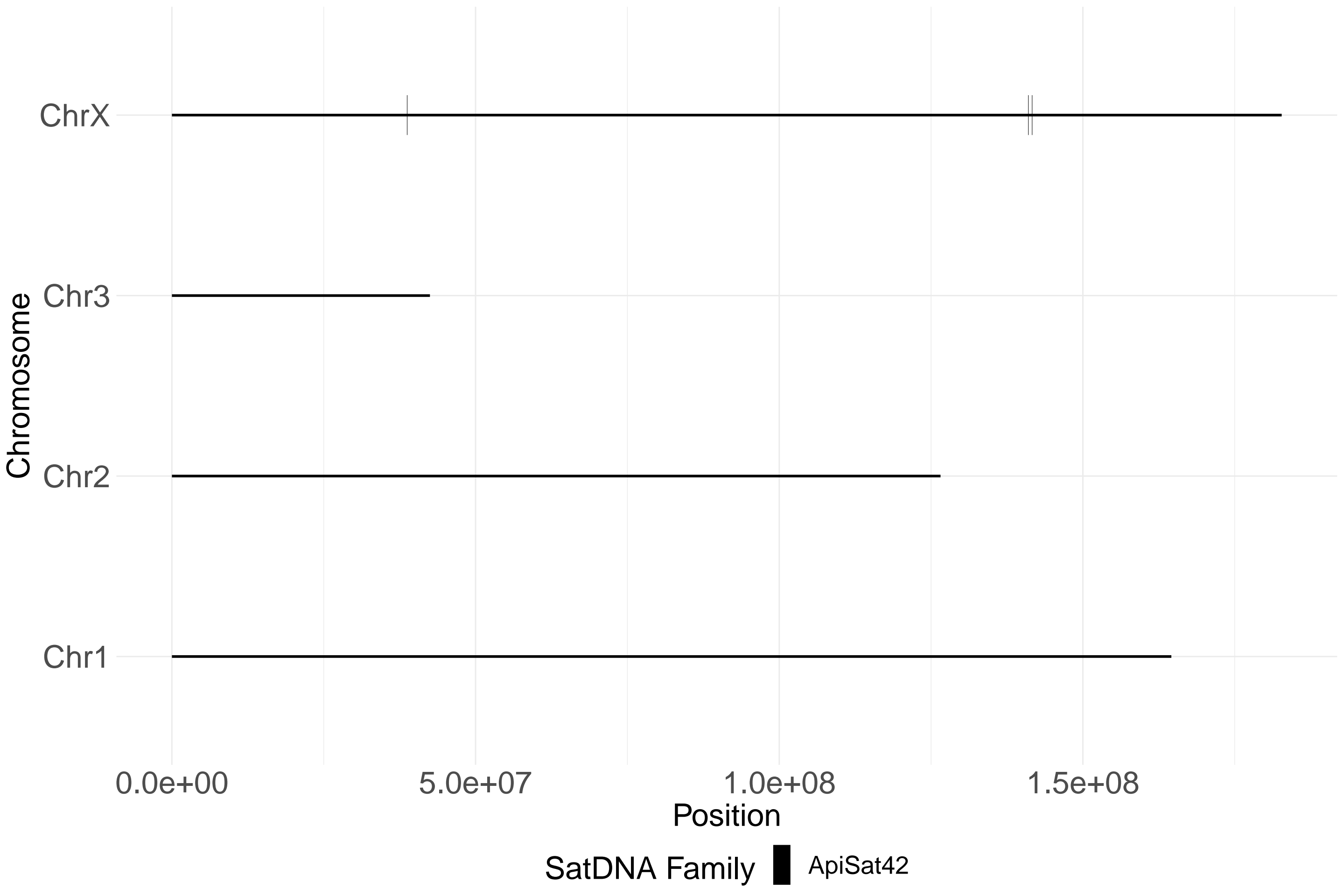

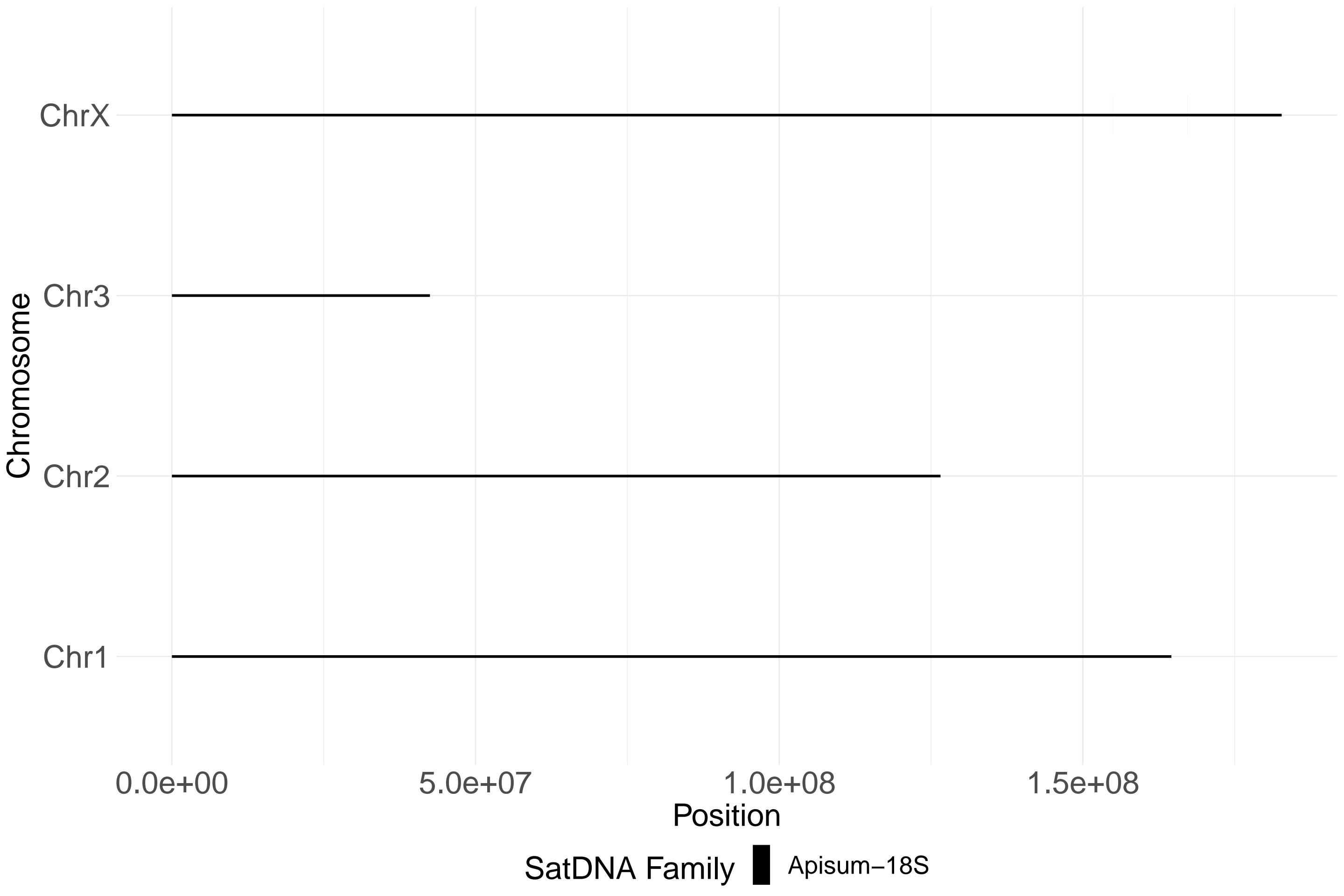

Supplement: evaf104_Supplementary_Data [file evaf104_supplementary_data.zip › R2_Supp_Figures/R2_Supp_Figure_3_chrismapp_individual.pdf]
